# xDecoder unlocks the potential of genomic foundation models for few-shot personal gene expression prediction

**DOI:** 10.1038/s44320-026-00238-1

**Published:** 2026-07-28

**Authors:** Shumin Li, Ruibang Luo, Yuanhua Huang

**Affiliations:** 1 School of Biomedical Sciences, University of Hong Kong, Hong Kong, China; 2 School of Computing and Data Science, University of Hong Kong, Hong Kong, China; 3 School of Artificial Intelligence, Shenzhen University, Shenzhen, China

**Keywords:** Computational Biology

## Abstract

Large-scale genomic language models (gLMs) hold promise for modeling gene regulation, yet their ability to capture personal gene expression variations remains unresolved. We developed xDecoder, a unified decoding framework that utilizes gLMs and sequence-to-function (S2F) embeddings to learn how personal genetic variation shapes gene expression from paired genome-transcriptome data. Compared to the pretrained genomic models, xDecoder with personalized DNA–RNA training makes cross-individual prediction tractable for seen genes in a few-shot setting. However, zero-shot prediction at unseen loci remains unreliable and gene-dependent, revealing a cross-locus transfer bottleneck of current sequence models. Experiments incorporating individual-level chromatin accessibility suggested that regulatory-state information important for unseen-locus prediction is not fully captured by current DNA-only models. Overall, these results highlight the potential utility of the few-shot setting, the limitations of DNA-only models, and point toward multi-omic, variant-aware frameworks as a promising direction for building personalized regulatory models.

## Synopsis

Few-shot personal gene expression prediction from DNA sequence was improved by xDecoder, while zero-shot analyses revealed a cross-locus transfer bottleneck linked to missing regulatory-state information.

- Cross-individual prediction for seen genes was improved by xDecoder trained on personal DNA-RNA pairs using gLM and S2F embeddings.
- Sequence-based modeling enabled potential discovery of candidate cis-regulatory variants at loci not applicable to gene-specific statistical models.
- Zero-shot transfer to unseen loci was generally unreliable, with a weak signal observed in more cis-predictable genes.
- Increasing the number of training individuals improved seen-gene performance, whereas zero-shot performance at unseen loci remained near zero.
- Matched ATAC-seq improved unseen-locus prediction, while ATAC-only controls indicated that measured chromatin accessibility largely explains the gain.

## Abstract (web summary)

Few-shot personal gene expression prediction from DNA sequence was improved by xDecoder, while zero-shot analyses revealed a cross-locus transfer bottleneck linked to missing regulatory-state information.

## Graphical abstract

**Figure:**
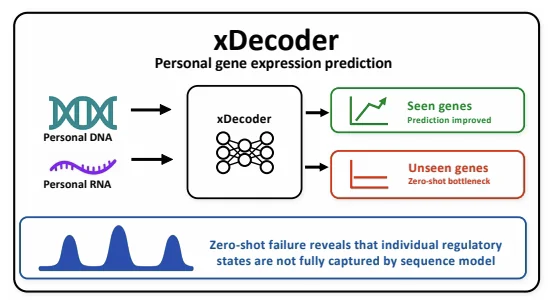

**Figure:**
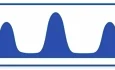

## Introduction

Building accurate genotype-to-phenotype models remains central to modern biology, often with genotype-to-expression as the junction. While expression quantitative trait loci (eQTL) studies have identified thousands of loci associated with gene expression, these approaches are primarily limited to common variants due to their reliance on population-scale data. As a result, rare and novel variants—key to understanding disease mechanisms and regulatory biology—remain difficult to interpret.

Sequence-to-function (S2F) models such as Enformer (Avsec et al, 2021) and Borzoi (Linder et al, 2025) address this gap by mapping genomic sequence to regulatory outputs using deep learning. Trained on reference genomes with multiomic labels across diverse cellular contexts, they accurately predict average gene expression across genes and tissues. However, their ability to capture transcriptomic variation across individuals, driven by personal genetic variants, remains limited (Huang et al, 2023; Sasse et al, 2023). Recent efforts have explored training or fine-tuning S2F models on personal genomes, which improved performance on seen genes but failed to generalize to unseen genes (Drusinsky et al, 2026; Rastogi et al, 2024; Spiro et al, 2026), although they have shown some promise in the related task of variant effect prediction (He et al, 2025). More recently, AlphaGenome was released to improve variant effect prediction; its ability to model gene expression from personal genomes also has not yet been evaluated (Avsec et al, 2026).

Genomic language models (gLMs) offer a promising approach. Pretrained via self-supervised learning on DNA sequences, they learn generalizable representations for diverse downstream tasks. While early results indicate that these models hold significant potential (Brixi et al, 2026; Dalla-Torre et al, 2025), their ability in regulatory genomics remains a challenge (Tang et al, 2025), and it is still unknown whether they can complement or extend S2F models in personal gene expression prediction.

Genomic foundation models, including both S2F models and gLMs, share structural elements such as self-attention encoders, yet they diverge in training strategy and supervision: S2F models exploit multiomic annotations across cellular contexts, whereas gLMs rely on DNA sequence-only pretraining. This contrast raises key open questions: (1) To what extent do genomic foundation models capture individual-level regulatory variation when fine-tuned with paired DNA–RNA data, and how far can they generalize to unseen individuals and genomic loci? (2) What additional biological information is required to overcome known challenges in zero-shot personalized expression prediction?

To address the questions, we developed xDecoder (cross-individual expression decoder), a unified decoding framework based on paired genome transcriptome data, to utilize the representation power of three gLMs-Evo2-7B (Brixi et al, 2026), Nucleotide Transformer v2 (NT) (Dalla-Torre et al, 2025), Caduceus (Schiff et al, 2024), and three S2F models-Enformer, Borzoi, and AlphaGenome on the task of personal gene expression prediction. We evaluated performance across individuals for both seen and unseen genes, revealing that gLMs and S2F models exhibit complementary strengths. We show that individual-level DNA–RNA fine-tuning improves cross-individual performance for seen genes, yet remains insufficient to address generalization to unseen genomic contexts. Incorporating personal epigenomic profiles, such as ATAC-seq tracks, moderately but significantly improved prediction for unseen genes—highlighting the potential of integrating multi-omics data and foundation models to bridge current gaps in personalized expression modeling.

## Results

### Overview of xDecoder

**Figure 1:**
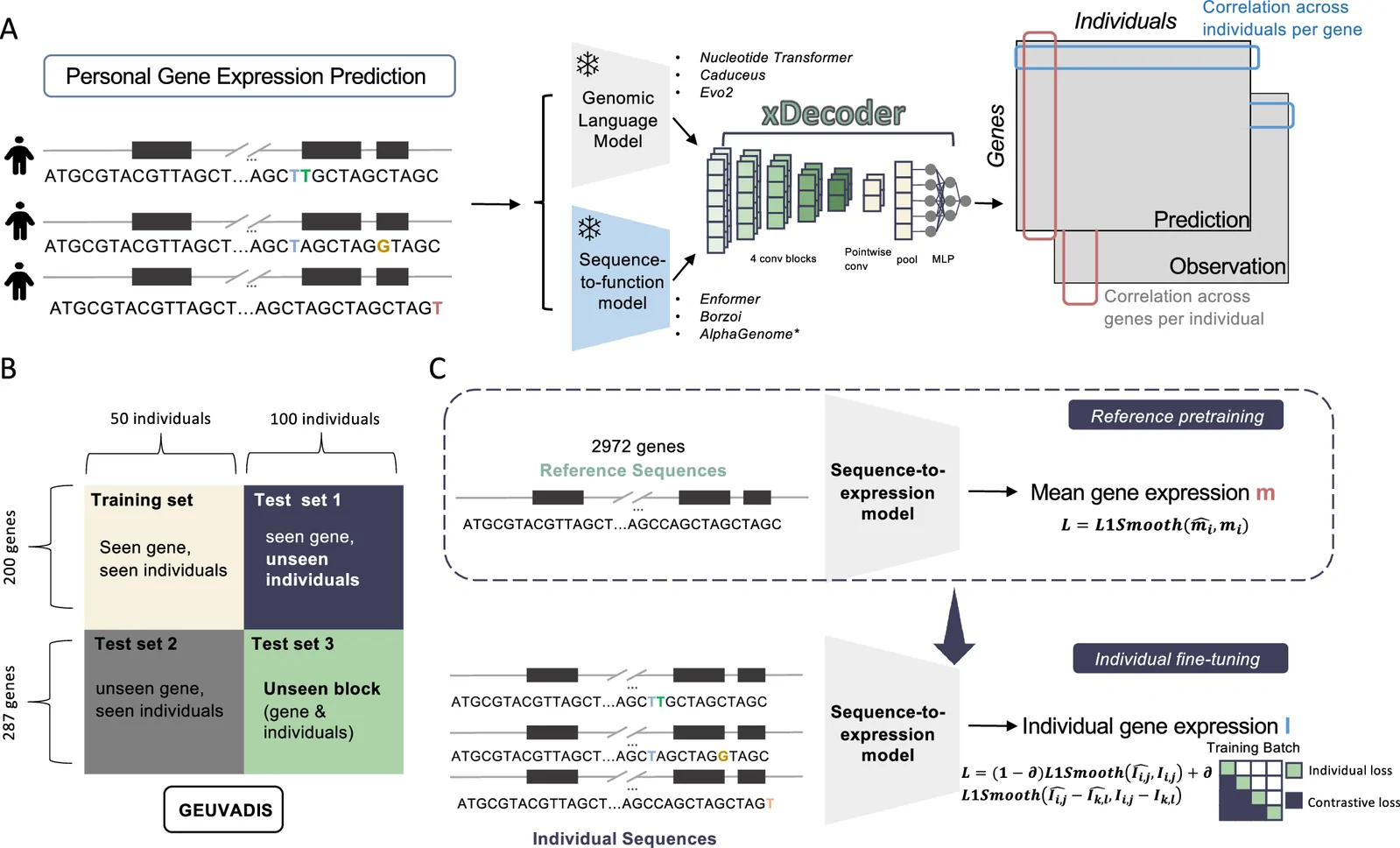
Extending gLMs and S2F models for personalized gene expression prediction. (**A**) Overview of the study. Using matched personal genome-transcriptome data, token-level embeddings from S2F models (Enformer, Borzoi) and genomic language models (Nucleotide Transformer, Caduceus, Evo2) were fed into a unified multi-layer CNN decoding tower for personal gene expression prediction. All models were used at their full receptive fields, except Evo2, which was limited to 64,000 bp for computational feasibility. AlphaGenome was evaluated using its original pretrained weights without fine-tuning. Performance was assessed using both across-individual and across-gene Spearman correlations. (**B**) Individual-related data partitions across genes and individuals for the dataset of the GEUVADIS consortium. (**C**) Training strategies. Models were trained using one of three approaches: reference-only, individual-only, or reference pretraining followed by individual fine-tuning. Strategies involving individual genomes were optimized with a combination of individual-level and contrastive loss. Source data are available online for this figure.

We utilized the representation power of the gLMs and S2F models on their ability to predict personalized gene expression using paired genome-transcriptome data from GEUVADIS (Lappalainen et al, 2013). These data include lymphoblastoid cell line (LCL) RNA-seq and high-quality genotypes from 1000 Genomes Project (1KG) individuals. We used log-TPM processed by Rastogi et al (2024) and reconstructed personalized DNA sequences by substituting SNPs from phased 1KG genotypes into the reference genome. From a total of 3259 genes with significant *cis*-eQTLs in European individuals, we selected 2972 genes for training with reference sequence and population-average expression, 200 randomly sampled genes for training with personal genomes, and 287 genes on chromosomes 5 and 10 for held-out evaluation. This chromosome-based split was designed to provide a stringent locus-disjoint evaluation, and the held-out genes showed a comparable *cis*-predictability pattern to genes on the remaining chromosomes (Appendix Fig. S1). The dataset included 50 individuals for training and 100 for testing. (Fig. 1A,B, “Methods”).

We developed xDecoder, a lightweight CNN-based prediction tower that consumes the full token-level embeddings exported by each model. We avoid the [CLS] token and global mean pooling because, for a given gene, only a tiny fraction of positions differ across individuals (~0.01%). Instead, we preserve positional resolution and aggregate neighborhoods with small stacked depthwise-separable convolutional layers; we then apply pooling and an MLP, averaging per-haplotype predictions (Fig. 1A). We implemented three training strategies (Fig. 1C): (1) reference-only models trained on population-average expression and the reference genome (“r-”); (2) individual-only models trained on personalized sequences and matched expression profiles (“p-”); and (3) combined models pretrained on the reference genome and fine-tuned with individual-level data (“rp-”). For individual-based models, we applied a hybrid loss function that combined a smooth L1 loss on individual predictions with a contrastive component to emphasize inter-individual and inter-gene variations within each batch. Specifically, AlphaGenome was evaluated using its original model without fine-tuning, as its pretrained weights were not publicly available at the time of our assessment.

For the gene-by-individual expression prediction, there are two levels of tasks (Fig. 1A): In the cross-gene variability (column vector), xDecoder with S2F embeddings performs significantly better than gLM embedding (Appendix Fig. S2), as S2F encoders are trained with supervised functional targets (including expression-related readouts) at these loci in similar cellular contexts, whereas gLMs are pretrained self-supervised on sequence only without functional labels. Notably, xDecoder using one-hot embeddings showed substantially weaker generalization, with Spearman correlation close to zero, than the gLM-based xDecoder. This suggests that the advantage of gLMs is driven less by the tokenization and more by the sequence representations learned through self-supervised pretraining (Appendix Table S1). In contrast, cross-individual variability (row vector) remains an unsolved challenge that we primarily focus on in this study using seen and unseen genes (Fig. 1A).

### Few-shot learning is feasible for seen genes, but zero-shot generalization remains fundamentally limited

We structured our assessment around two scenarios: few-shot prediction for seen genes and zero-shot prediction for unseen genes (Tests 1 and 3, Fig. 1B). We first asked whether models without individual-level DNA–RNA training could capture personal expression variation. Consistent with recent studies (Huang et al, 2023; Sasse et al, 2023), reference-only pretrained S2F models (Enformer, Borzoi, and AlphaGenome) and reference-trained xDecoder variants showed limited ability to predict cross-individual expression differences. Among these reference-only baselines, AlphaGenome achieved the strongest performance, but its correlations remained modest, indicating that pretrained S2F models alone are still insufficient for personal expression prediction.

**Figure 2:**
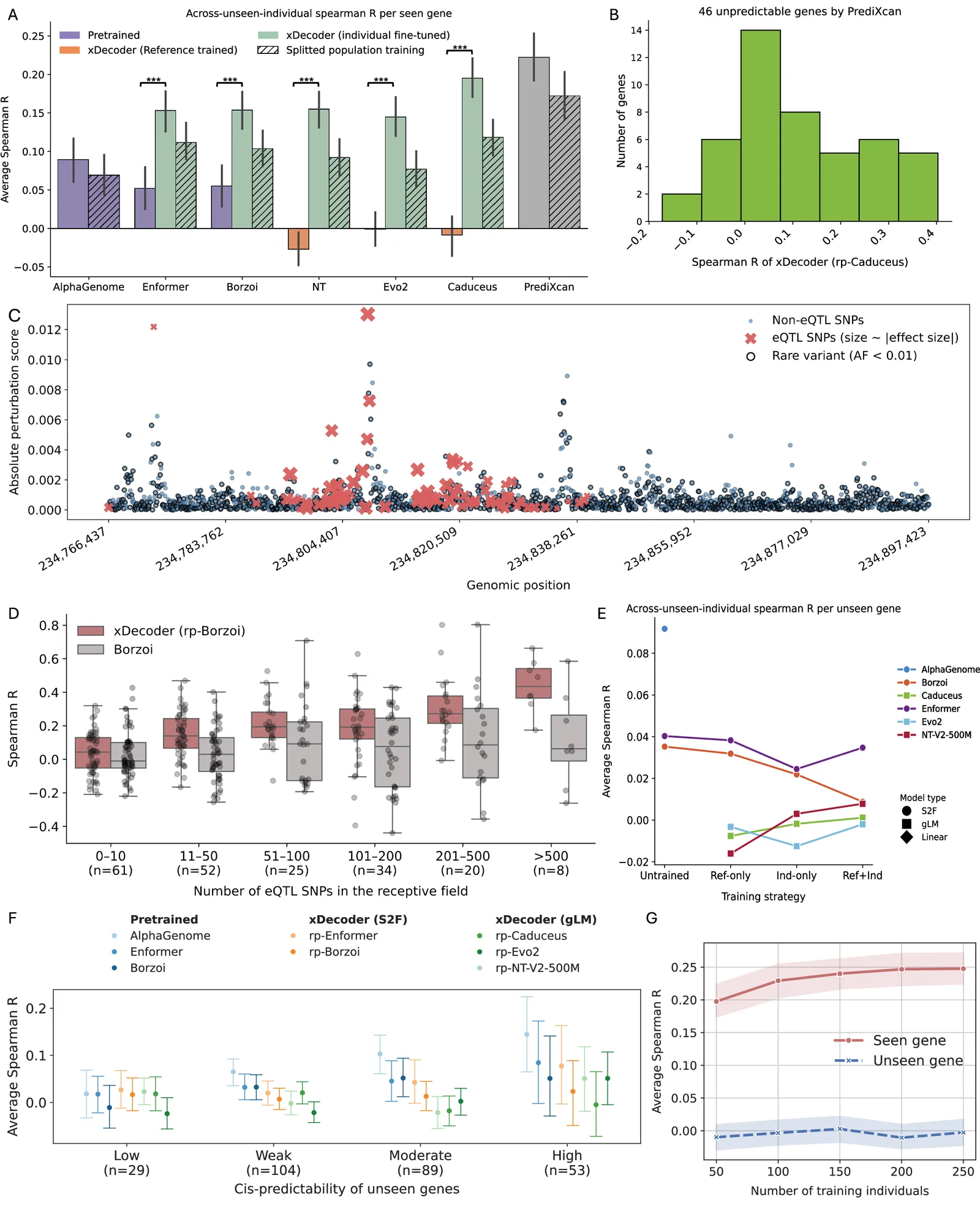
Comparison of personalized expression prediction across models and tasks. (**A**) Across-unseen-individual correlation per seen gene of pretrained models and xDecoder supplied by gLMs and S2F embeddings. The statistical model PrediXcan was trained per gene using genotypes of common variants (allele frequency ≥0.05). Two evaluation settings: (i) random-gene evaluation using 200 genes with 50 training and 100 evaluation individuals from mixed populations (Test set 1), (ii) population-split training by holding out individuals from the Yoruba in Ibadan (YRI) population on the same set of 200 random genes (“Methods”). ^***^ indicates *P* < 0.001 by two-sided paired *t* test; exact *P* values for marked comparisons, from left to right, are 1.44 × 10^−8^, 7.57 × 10^−9^, 5.58 × 10^−23^, 1.43 × 10^−15^, and 1.33 × 10^−24^. (**B**) Correlation performance of xDecoder (rp-Caduceus) on 46 genes for which PrediXcan yields identical predictions across individuals. (**C**) Variant-level perturbation analysis for a PrediXcan-uninformative gene AL160408.3 using xDecoder (rp-Caduceus). (**D**) Spearman correlations of Borzoi and xDecoder (rp-Borzoi) across unseen individuals for seen genes, binned by the number of eQTL SNPs in the receptive field. The center line indicates the median; box bounds indicate the 25th and 75th percentiles; whiskers extend to the lowest and highest values within 1.5 × the interquartile range. Overlaid points represent individual genes and show the full distribution, including values outside the whiskers. Exact *P* values for paired comparisons between Borzoi and xDecoder (rp-Borzoi) within each bin, from left to right, are 0.738, 2.40 × 10^−5^, 0.0374, 0.00408, 0.0234, and 0.0214 by two-sided paired *t* test. (**E**) Across-unseen-individual correlation per unseen gene (Test set 3). Specifically, PrediXcan (seen genes only) is shown as a reference: it is trained and evaluated on the same genes and cannot extrapolate to unseen genes. (**F**) Mean zero-shot cross-individual Spearman R for unseen genes stratified by PrediXcan-based *cis*-predictability using gene-specific ElasticNet models trained on common *cis* variants (“Methods”). Noted that unpredictable genes by the ElasticNet model were excluded. (**G**) Learning curves for seen- and unseen-gene prediction using rp-Caduceus with a 64-kb TSS-centered input. (**A**, **F**, **G**) Central values represent mean Spearman *R* across genes; error bars in (**A**, **F**) and shaded regions in (**G**) indicate 95% bootstrap confidence intervals for the mean estimated from 1000 gene-level bootstrap resamples. Source data are available online for this figure.

**Figure EV1:**
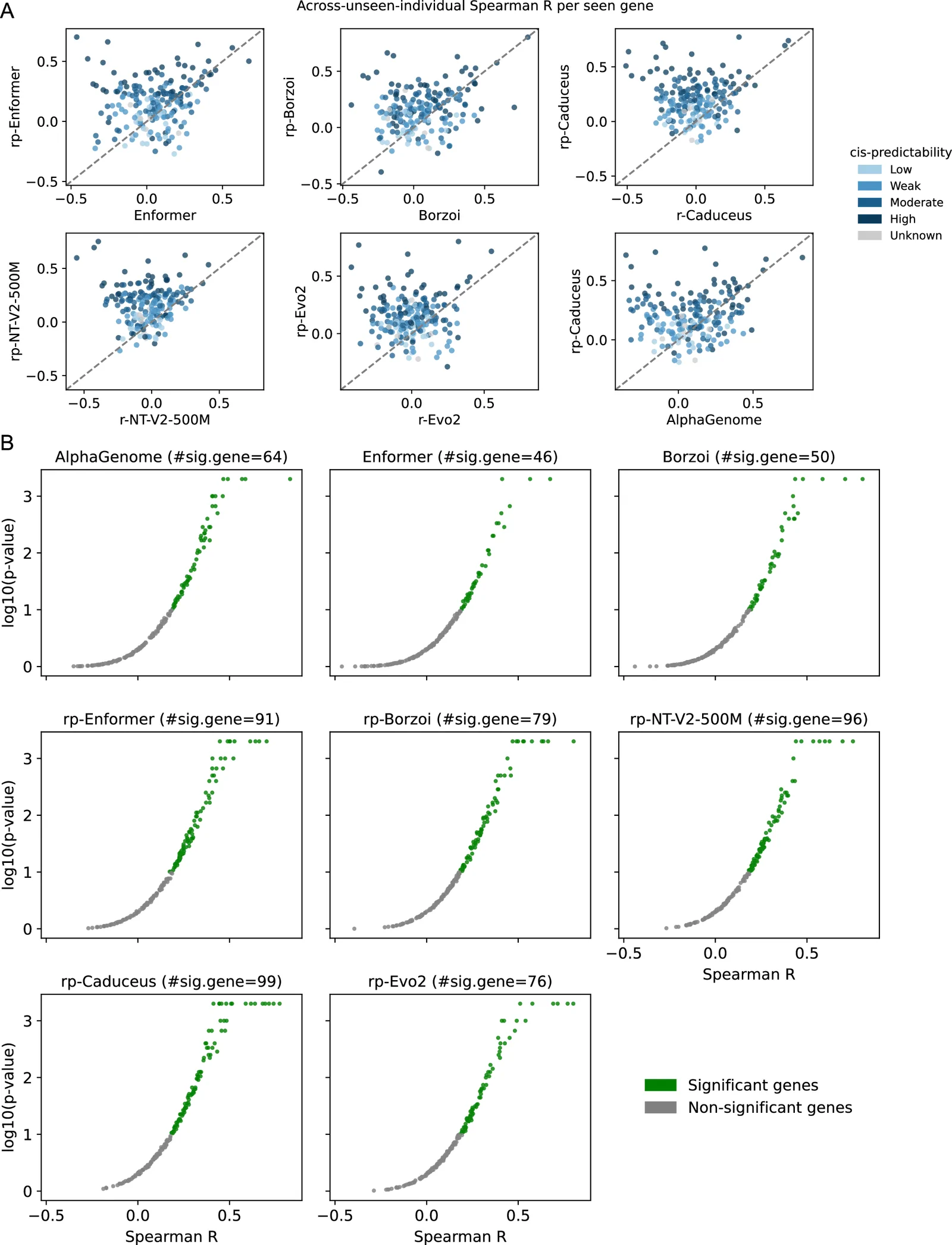
Individual fine-tuning improves few-shot prediction for seen genes. (**A**) Paired comparison of across-unseen-individual Spearman correlations between reference-trained and individually fine-tuned xDecoder models across seen genes. Each point represents one gene, and colors indicate PrediXcan-based *cis*-predictability groups. (**B**) Permutation-based significance analysis of cross-individual prediction performance. For each gene, observed expression values were permuted across testing individuals to generate a gene-specific null distribution (*n* = 2000), and genes with one-sided empirical *P* < 0.1 were defined as significant.

We next evaluated whether paired genome-transcriptome training could improve prediction for genes observed during training. In this few-shot seen-gene setting, xDecoder consistently improved cross-individual correlation, achieving  > threefold gains over pretrained Enformer and Borzoi, as well as over reference-trained xDecoder variants (from 0.089 to 0.194) (Fig. 2A; Appendix Figs. S3 and S4). Fine-tuning at the individual level also corrected the sign of correlation for a subset of genes (Fig. EV1A) and increased the number of genes showing significant cross-individual correlation in permutation tests (e.g., 99 for rp-Caduceus versus 64 for reference-pretrained AlphaGenome; Fig. EV1B). For example, for *HLA-DQA1*, the pretrained Enformer and AlphaGenome produced negative correlations of −0.257 and −0.153, respectively, while all individual fine-tuned xDecoder models achieved Spearman correlations above 0.3, with rp-Caduceus reaching 0.74 (Appendix Fig. S5). Similar improvements were observed for METTL18 (Appendix Fig. S6), where personal genome training increased Enformer’s correlation from −0.289 to 0.489. An exception was PPAN, where fine-tuning reduced performance but maintained the correct direction for rp-Caduceus and rp-Borzoi, which provide finer-resolution embeddings (Appendix Fig. S7). Overall, xDecoder supplied by different S2F and gLM embeddings was comparable in capturing individual variations for seen genes, with optimal gLM embeddings outperforming the best S2F embeddings (mean Spearman R: 0.194 for rp-Caduceus vs. 0.153 for rp-Borzoi). In addition, rp-Caduceus achieved performance comparable to the PrediXcan-style gene-specific linear model. Notably, rp-Caduceus successfully modeled 46 genes that PrediXcan failed to predict-i.e., where PrediXcan predicted identical values across individuals (Fig. 2B). This result identifies a subset of genes where fixed genotype weights are uninformative while sequence-based representations still contain predictive signal. As an exploratory follow-up, we performed variant-level perturbation analysis for one representative gene from this prediXcan unpredictable set and found that high-scoring xDecoder perturbations overlapped known eQTL SNPs with large observed effects (Fig. 2C), suggesting that xDecoder can generate variant-level hypotheses in such loci.

**Figure EV2:**
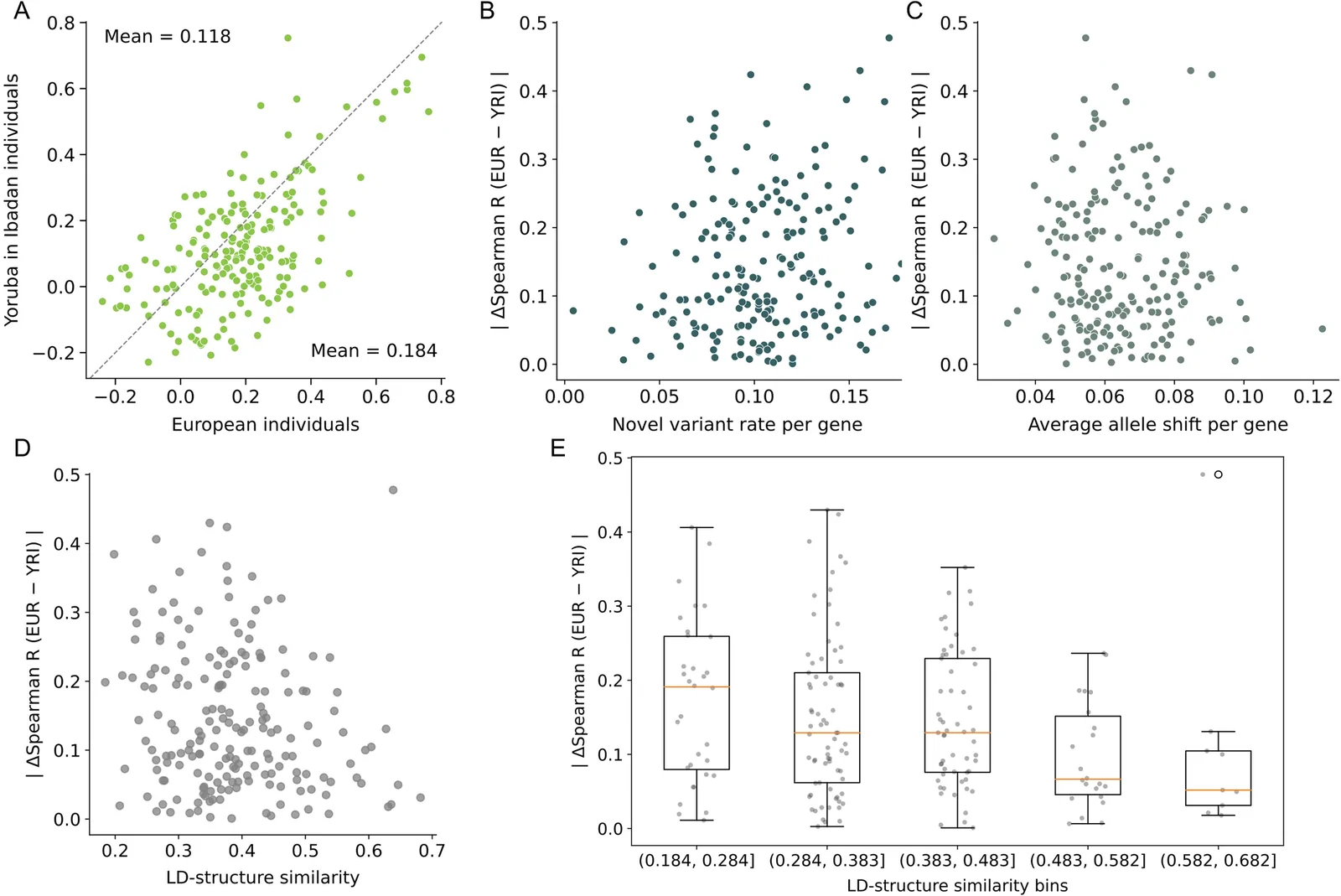
Analysis of cross-ancestry performance using the rp-Caduceus model. (**A**) Gene-level prediction performance of the same European-trained model evaluated on European and YRI test individuals. (**B**) Relationship between the absolute EUR-to-YRI performance gap and the novel variant rate per gene, defined as the fraction of common variants (MAF ≥0.05) present in one population but absent in the other within the 131,070 bp TSS-centered context. (**C**) Relationship between the absolute performance gap and mean allele-frequency shift per gene, defined as the average absolute allele-frequency difference between European and YRI individuals across variants in the same region. (**D**) Relationship between the absolute performance gap and LD-structure similarity. LD-structure similarity was measured as the Jaccard index between strong-LD variant-pair sets identified separately in European and YRI populations from the Ensembl 1000 Genomes panel, using *R*^2^ > 0.8. Higher values indicate a more similar local LD organization between populations. (**E**) Distribution of the absolute performance gap after grouping genes by LD-structure similarity.

We next evaluated the cross-population generalization using a population-split experiment, training on 50 European individuals and evaluating the same trained model on two held-out test sets: 89 Yoruba individuals from Ibadan (YRI) and 90 European individuals extracted from the original mixed test set after excluding YRI samples. In this setting, the benefit of individual training decreased (Figs. 2A and EV2A), reflecting the known challenge of cross-population transfer (Keys et al, 2020; Mogil et al, 2018; Okoro et al, 2021; Rastogi et al, 2024). To investigate potential factors associated with this performance gap, we examined three gene-level indicators within the 131,070 bp rp-Caduceus input context: novel variant rate, mean allele-frequency shift, and LD-structure similarity between European and YRI individuals (“Methods”). No single factor fully explained the cross-ancestry performance drop. However, genes with larger EUR-to-YRI performance gaps tended to have higher novel variant rates and lower LD-structure similarity, whereas average allele-frequency shift showed a weaker association (Fig. EV2B–E). These results suggest that ancestry-specific variation and local LD differences may be associated with reduced transferability, but they do not establish a single mechanistic explanation for the cross-ancestry gap.

To further dissect per-gene performance, we stratified genes by the number of eQTL SNPs within the model receptive field. Genes with higher eQTL density tended to show improved correlation, consistent with the expectation that input contexts containing more informative *cis*-regulatory variants are easier to predict (Fig. 2D; Appendix Fig. S8). In addition, xDecoder (rp-Borzoi) achieved higher accuracy than the pretrained Borzoi model across all bins. Consistently, genes with more significant eQTL SNPs (smaller *P* values) showed better performance (Appendix Fig. S9). Although we did not include INDELs in the personal sequence, we evaluated their potential impact and found that the number of INDELs is not related to the performance (Appendix Fig. S10).

We next assessed zero-shot prediction for unseen genes in unseen individuals (Fig. 2E). None of the models performed reliably in this setting, while some models exhibited a long-tailed distribution with a small subset of genes showing positive correlations (Appendix Fig. S11). For context, PrediXcan trained separately for each gene achieves a mean Spearman correlation of 0.216. We further stratified the unseen genes by PrediXcan *cis*-predictability (“Methods”; Fig. 2F). It shows zero-shot performance tended to increase for genes with stronger *cis* signal-most notably for AlphaGenome-yet remained substantially below the gene-specific supervised PrediXcan reference even among moderately/highly cis-predictable genes (Appendix Fig. S12A,B), highlighting the difficulty of transferring *cis*-regulatory rules across loci. These findings indicate that zero-shot generalization is gene-dependent and partially recoverable for more strongly *cis*-predictable genes, but remains inconsistent and substantially below gene-specific supervised models, leaving reliable per-gene prediction at unseen loci unresolved. We further examined whether performance is limited by training size by increasing the number of training individuals (*n* = 50, 100, 150, 200, 250) using xDecoder (rp-Caduceus). Seen-gene prediction across unseen individuals improved with more training individuals, with diminishing returns at higher *n* (Fig. 2G), indicating that few-shot prediction had not saturated at *n* = 50. In contrast, zero-shot prediction for unseen genes remained close to zero across all training sizes (Fig. 2G), suggesting that this limitation is not simply due to the number of training individuals but reflects a persistent cross-locus generalization bottleneck.

Full-parameter fine-tuning of S2F models and xDecoder under the same training strategies resulted in comparable performance in modeling individual-level variation. xDecoder showed a modest advantage when paired with Enformer embeddings (Appendix Fig. S13). Similar trends were observed when restricting the receptive field to 64 kb (Appendix Fig. S14).

In summary, taking each gene as an independent output variable, we may view Test 1 as few-shot learning and Test 3 as zero-shot learning. Despite the limited power in the zero-shot prediction, xDecoder with DNA-only inputs showed promise in few-shot learning (Fig. 2A). Because sequence-based models operate directly on DNA sequence, they can, in principle, evaluate sequence perturbations outside the fixed variant-weight space of linear predictors, suggesting potential utility in settings where rare or novel variant weights are unavailable. However, the inability of existing models to generalize across loci highlights a bottleneck of DNA–RNA fine-tuning alone.

### Chromatin accessibility helps explain the limitations of DNA-only zero-shot prediction

Based on the above results, fine-tuning on individual-level DNA–RNA pairs alone may be insufficient to capture inter-individual variation across unseen genes for both S2F and gLM representations. To investigate this limitation, we focused on two key factors that could help diagnose or mitigate this limitation: (1) single-base-resolution embeddings, which better capture the effects of individual SNPs, and (2) integration of individual-level multi-omic data.

As a proof of concept, we incorporated individual-level ATAC-seq tracks into Caduceus embeddings by adding a base-resolution ATAC channel and trained the models using the same strategy as described above, with 50 individuals for training and 44 for evaluation, and a sequence window centered on the TSS. (“Methods”, Table 1). We focused on the most challenging task—predicting expression across unseen individuals for unseen genes. The results demonstrated that reference pretraining followed by individual fine-tuning provided more consistent benefits than individual-only training, indicating the importance of diverse gene contexts in the training.

**Table 1:** Gene expression prediction performance (mean Spearman *R*) across different training strategies and input modalities for Caduceus and ATAC-only.

Training strategy	Input	Task 1	Task 2	Task 3
Ref-only	DNA+ATAC	0.066 [0.035, 0.097]	0.108 [0.082, 0.134]	0.343 [0.340, 0.347]
	DNA only	0.017 [−0.014, 0.047]	0.010 [−0.015, 0.035]	0.121 [0.118, 0.124]
Indiv-only	DNA+ATAC	0.222 [0.190, 0.254]	0.052 [0.030, 0.076]	0.242 [0.237, 0.246]
	DNA only	0.260 [0.231, 0.291]	-0.001 [-0.027, 0.025]	0.066 [0.062, 0.070]
Ref + Indiv	DNA+ATAC	0.248 [0.218, 0.279]	0.129 [0.103, 0.155]	0.365 [0.361, 0.370]
	DNA only	0.241 [0.209, 0.273]	−0.003 [−0.030, 0.023]	0.160 [0.156, 0.163]
Sum of ATAC	ATAC	0.123 [0.096, 0.151]	0.140 [0.115, 0.166]	NA

Values are reported as mean [95% CI], where confidence intervals were obtained by bootstrap resampling of genes (*B* = 10,000). Task 1: Across unseen individuals per seen gene (Test set 1 in Fig. 1B); Task 2: Across unseen individuals per unseen gene (Test set 3 in Fig. 1B); Task 3: Across unseen genes per unseen individual (Test set 3 in Fig. 1B).

For comparability, predictions that were identical across individuals were assigned a Spearman *R* of 0.

**Figure 3:**
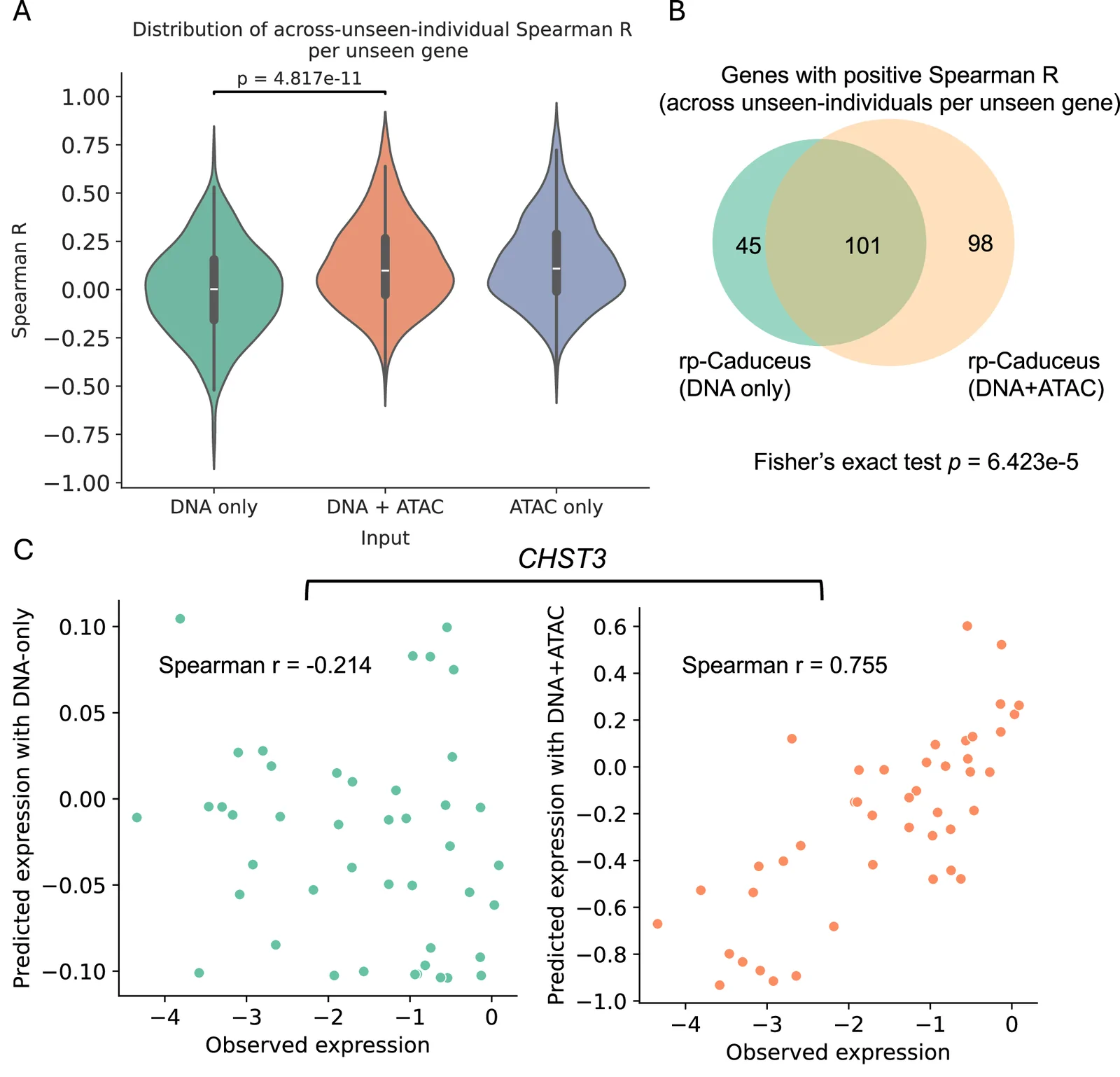
xDecoder (rp-Caduceus) cross-individual performance for unseen genes with and without ATAC integration. (**A**) Distribution of Spearman correlations across unseen individuals for unseen genes using rp-Caduceus with DNA-only input, rp-Caduceus with DNA+ATAC input, and a simple ATAC-only baseline computed by summing ATAC-seq peak signals within the same TSS-centered window. Genes with identical predictions across individuals were assigned a Spearman R of 0. Significance was assessed between DNA-only and DNA+ATAC using a two-sided paired *t* test. Violins show the distribution across genes; the inner box indicates the interquartile range, and the central line indicates the median. (**B**) Venn diagram of genes with positive Spearman correlations for the across-unseen-individual per unseen-gene task using rp-Caduceus. (**C**) Example gene *CHST3*, for which the DNA-only model predicted the opposite direction of inter-individual variation, whereas adding ATAC achieved strong concordance with observed expression. Source data are available online for this figure.

Importantly, incorporating ATAC peaks significantly improved cross-individual correlations for unseen genes (0.129 vs. −0.003; *P* < 5e-11 by two-sided paired *t* test; Fig. 3A; Appendix Fig. S15A). With ATAC integration, rp-Caduceus identified 199 of 287 genes with positive cross-individual correlations, compared with 146 using DNA alone, indicating that ATAC signals largely corrected correlation direction for most genes (Fig. 3B; *P* = 6.4e-5 Fisher’s exact test). For example, for one unseen gene of CHST3, the DNA-only model predicted the opposite direction of inter-individual variation, whereas adding the ATAC track achieved a Spearman correlation of 0.755 (Fig. 3C). Additional examples are shown in Appendix Fig. S16, while a subset of genes exhibited little or no improvement (Appendix Fig. S17).

**Figure EV3:**
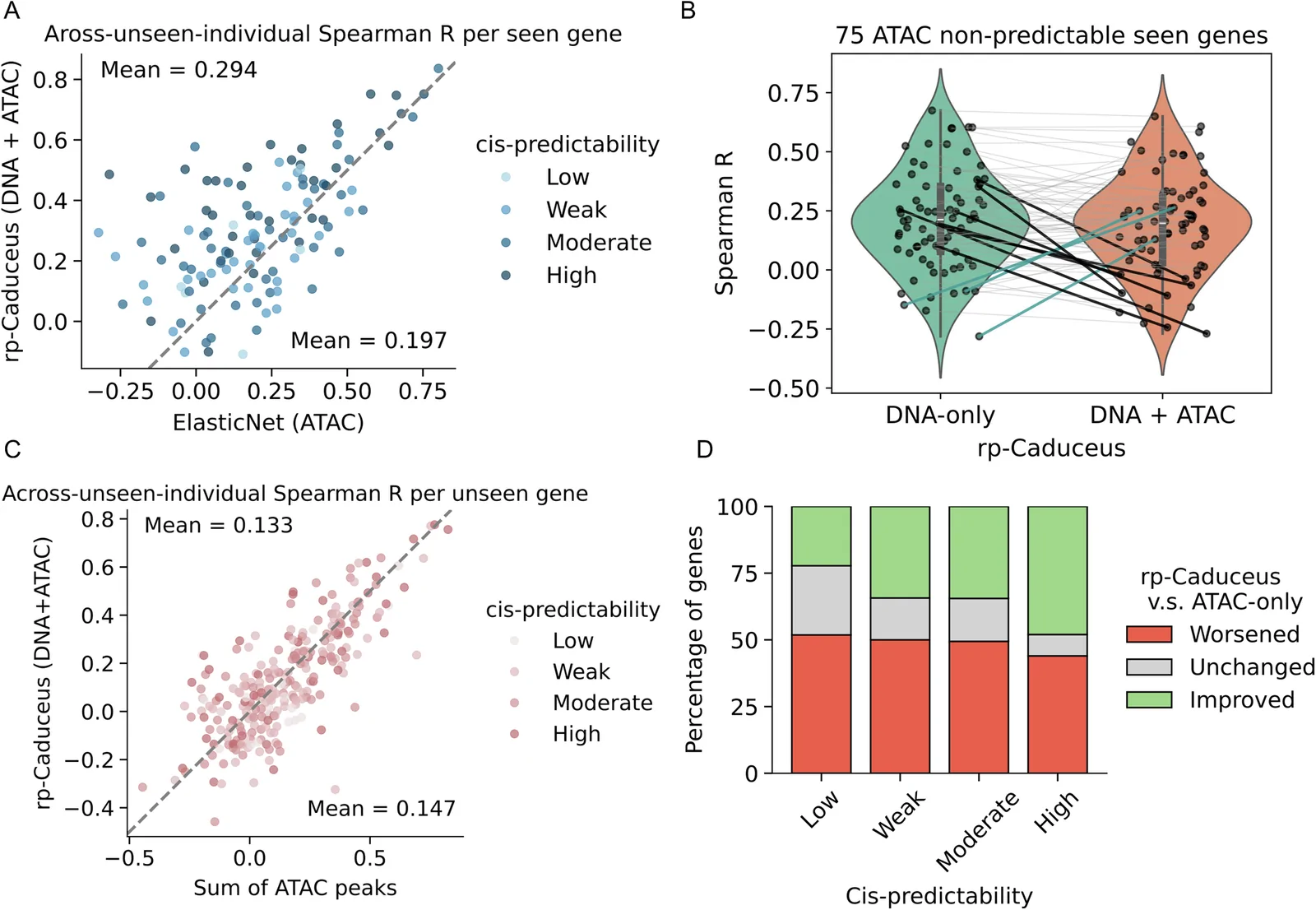
ATAC-only analyses in seen- and unseen-gene settings. (**A**) Comparison between a supervised ATAC-only ElasticNet baseline and rp-Caduceus (DNA+ATAC) in the seen-gene setting. For each seen gene, the ATAC-only ElasticNet model was trained using ATAC features summarized within the same 64 kb TSS-centered context window used by rp-Caduceus. Noted that 125 out of 200 seen genes are valid for the ATAC-only model. The center line indicates the median, box bounds indicate the 25th and 75th percentiles, and whiskers extend to the lowest and highest values within 1.5 × the interquartile range. (**B**) Performance of rp-Caduceus with DNA-only and DNA+ATAC inputs on 75 seen genes for which the ATAC-only ElasticNet baseline produced uninformative predictions. (**C**) Comparison between an unsupervised ATAC-only baseline and rp-Caduceus (DNA+ATAC) in the unseen-gene setting. The ATAC-only score was computed by summing the ATAC signal across the same 64 kb genomic context and correlating this score with observed expression across held-out individuals. Noted that 275 out of 287 unseen genes are valid for the ATAC-only model. (**D**) Stratification of unseen genes by *cis*-predictability. Genes were classified as improved, unchanged, or worsened according to the performance difference between rp-Caduceus (DNA+ATAC) and the ATAC-only baseline.

To distinguish whether the improvement from adding ATAC reflects model learning beyond a simple chromatin-accessibility proxy, we added ATAC-only analyses tailored to the two evaluation settings. In the seen-gene setting, RNA labels for the target genes are available during training, so we constructed a supervised ATAC-only baseline by training per-gene ElasticNet models using ATAC features summarized within the same 64 kb TSS-centered window used by rp-Caduceus. Specifically, ATAC peaks within the context window were aggregated into five regional features and used to predict expression across individuals under the same training and testing split. This baseline asks whether a simple gene-specific mapping from measured accessibility to expression is sufficient. In this setting, rp-Caduceus (DNA+ATAC) outperformed the ATAC-only ElasticNet baseline (0.294 vs 0.197 for 125 ATAC predictable genes, Fig. EV3A), indicating that the few-shot model is not merely reproducing a supervised accessibility–expression proxy. Rather, paired genome–transcriptome training appears to learn gene-specific individual-level variation beyond what is captured by this ATAC-only linear model. Specifically, for 75 ATAC-non-predictable genes, rp-Caduceus (DNA-only) outperforms rp-Caduceus (DNA+ATAC), indicating some seen-gene predictions are better captured by sequence-derived signal than by the simple ATAC proxy (Fig. EV3B).

In the unseen-gene setting, a supervised per-gene ATAC model is not applicable because RNA labels from the held-out genes are not available during training. We therefore used a simple unsupervised ATAC-only gene-activity baseline by summing ATAC signal across the same 64 kb genomic context and correlating this score with observed expression across held-out individuals. This summed-ATAC baseline was competitive with rp-Caduceus (DNA+ATAC), supporting that measured ATAC can serve as a strong proxy for expression (Figs. 3A and EV3C; Table 1). Stratification by *cis*-predictability further showed that the relative benefit of rp-Caduceus (DNA+ATAC) over ATAC-only was more evident for genes with stronger *cis*-genetic signal, whereas ATAC alone was more competitive for genes with weak *cis*-genetic signal (Fig. EV3D). Nevertheless, both ATAC-only and DNA+ATAC models remained below the per-gene trained PrediXcan reference (Appendix Fig. S15B). Together, these results suggest that individual-level chromatin accessibility is critical for predicting personal gene expression at unseen genomic contexts. The competitive performance of the ATAC-only baseline indicates that measured ATAC provides regulatory-state information that is highly informative for expression variation. At the same time, the poor performance of DNA-only models in this setting suggests that current sequence models do not yet reliably infer individual-specific chromatin states from DNA sequence alone. Consistently, AlphaGenome-predicted gene-level ATAC signals showed only modest concordance with observed ATAC signals across individuals (mean Spearman *R* = 0.168; Appendix Fig. S18A), which may partly explain the limited zero-shot generalization of DNA-only models across loci (Appendix Fig. S18B). Therefore, rather than treating DNA+ATAC as a practical replacement for RNA-seq, we interpret this experiment as revealing a key bottleneck of current DNA-only models. A promising future direction is to use individual-level multi-omic data, including ATAC-seq, as auxiliary supervision or intermediate regulatory-state targets during training, with the goal of improving DNA-based prediction at unseen loci.

## Discussion

In summary, xDecoder provides a unified decoding framework to unlock the potential of genomic foundation models by effective few-shot learning and for diagnosing current barriers to zero-shot generalization. Our results support three conclusions. First, incorporating personal genome-expression pairs improves prediction for seen genes and can complement existing linear models such as PrediXcan. In particular, xDecoder identified a subset of genes for which PrediXcan produced no informative individual-level predictions, and exploratory perturbation analysis showed that the model can nominate candidate *cis*-regulatory variants within such loci (Fig. 2C). This suggests a potential path toward interpreting sequence perturbations beyond fixed common-variant eQTL weights, although direct validation on rare-variant and somatic-variant datasets remains necessary. Second, the benefit of adding personal paired training does not generalize to unseen loci, highlighting the challenge of learning transferable *cis*-regulatory grammar, even if the number of training individuals is increased (Fig. 2G). Third, incorporating matched individual-level ATAC-seq improves prediction at unseen loci. However, ATAC-only and DNA+ATAC models perform similarly in this setting, suggesting that the improvement is primarily driven by measured chromatin accessibility. Consistently, AlphaGenome-predicted ATAC signals showed only modest agreement with observed ATAC signals (Appendix Fig. S18), suggesting that current DNA-only models may not fully recover individual chromatin-accessibility states that are informative for expression prediction at unseen loci. By contrast, in the seen-gene setting, xDecoder with DNA+ATAC outperforms ATAC-only linear baselines, indicating that sequence-derived information can provide additional signal when paired DNA–RNA training data are available. Together, these results outline a roadmap for personalized regulatory modeling: combining pretrained sequence encoders with variant-aware modules and using individual-level multi-omic profiles as auxiliary supervision or intermediate regulatory-state targets to improve DNA-based generalization to unseen loci. A similar spirit was recently demonstrated in a cross-gene prediction model, EPInformer, where epigenetic-aware elements were derived from a longer range to improve the model efficiency (Lin et al, 2026). Several limitations should also be considered. Although xDecoder improves few-shot personal expression prediction over existing sequence-based models, its absolute accuracy remains modest and should not yet be interpreted as sufficient for reliable gene-level prediction from personal genomes. Future work should establish application-specific benchmarks for downstream use. For variant prioritization, an important next step is to systematically evaluate whether model-derived perturbation scores can prioritize eQTL-supported regulatory variants over matched background variants, using eQTL-based AUPRC as a primary metric. Any use in clinical interpretation would require substantially stronger calibration and independent validation. Finally, the present evaluation is limited to lymphoblastoid cell lines, and extending this framework to larger cohorts, multi-tissue settings, and context-specific personalized regulation remains an important direction.

## Methods

**Table:** Reagents and tools table

Reagent/resource	Reference or source	Identifier or catalog number
**Experimental models**		
N/A	N/A	N/A
**Recombinant DNA**		
N/A	N/A	N/A
**Antibodies**		
N/A	N/A	N/A
**Oligonucleotides and other sequence-based reagents**		
N/A	N/A	N/A
**Chemicals, enzymes, and other reagents**		
N/A	N/A	N/A
**Software**		
Python 3.8.18		
Enformer-pytorch 0.8.8	https://github.com/lucidrains/enformer-pytorch	
Borzoi-pytorch 0.4.1	https://github.com/johahi/borzoi-pytorch	
Caduceus	https://github.com/kuleshov-group/caduceus	
Nucleotide Transformer 0.0.1	https://github.com/instadeepai/nucleotide-transformer	
Evo2	https://github.com/arcinstitute/evo2	
AlphaGenome	https://github.com/google-deepmind/alphagenome	
bcftools 1.15.1	https://samtools.github.io/bcftools/bcftools.html	
scikit-learn 1.3.2	https://anaconda.org/conda-forge/scikit-learn	
**Other**		

### Personal gene expression dataset

We used paired whole-genome sequencing (WGS) and gene expression data from the GEUVADIS consortium (Lappalainen et al, 2013; Data ref: BioStudies, 2013), comprising lymphoblastoid cell lines (LCLs) from individuals in the 1000 Genomes Project Consortium (2015). For the main mixed-population analysis, a total of 150 individuals were randomly selected and partitioned into 50 training individuals and 100 held-out testing individuals. This split was used for the primary DNA-only benchmarking experiments, including both seen-gene and unseen-gene evaluations. For the cross-ancestry transferability analysis, we used a separate population-split setting, in which models were trained on 50 European individuals and evaluated on two held-out test sets: 90 European individuals and 89 Yoruba individuals from Ibadan, Nigeria (YRI). For the ATAC-integrated experiments, we restricted the analysis to individuals with matched genotype, RNA-seq, and ATAC-seq data. Among the 94 overlapping individuals, 50 were used for training, and the remaining 44 were used for testing.

This study focused on 3259 genes identified in the original GEUVADIS study as having significant *cis*-eQTLs in European individuals. Of these, 200 genes—excluding those located on chromosomes 5 and 10—were randomly selected for training with individual sequences, while 2972 genes were used for training with reference sequences. The remaining 287 genes from chromosomes 5 and 10 were reserved for testing. We used log-transformed TPM gene expression values and annotated transcription start sites (TSS) processed by Rastogi et al (2024) from their GitHub repository (Data ref: Github, 2024). In addition, we constructed a reference genome-based dataset using hs37d5 paired with population-average gene expression levels computed from all 462 individuals. For each gene, a sequence window centered on the TSS was provided as input to all models. We used the maximum receptive field of each model, except for Evo2, where a 64,000-bp window was applied to avoid prohibitive computational cost. Sequences were reverse-complemented for genes located on the negative strand.

Individualized input sequences were constructed using phased genotypes from the 1000 Genomes Project. We generated personalized DNA sequences using *bcftools consensus* (Li, 2011), including only single-nucleotide variants (SNVs) with the options -H 2pIu[1pIu] and -i “type=’snp’".

### Gene expression prediction using gLM and S2F embeddings

To evaluate the gLM and S2F model embeddings, we selected the output layer according to either the authors’ recommendations or the conventional choice of the final embedding layer. Specifically, we used the output of layer blocks.28.mlp.l3 from Evo2-7B as it is recommended from the official repository, the final embeddings from Enformer, Borzoi, Caduceus-ph, and NT. We constructed a multi-layer CNN to process the embedding inputs. The input to the model consists of a sequence of embedding vectors with shape (sequence length × embedding dimension). For Evo2-7B, we limited the sequence length to 64,000 due to its computational cost, while for other models, we used their full receptive fields: 12,288 for NT, 131,070 for Caduceus-ph, 196,608 for Enformer, and 524,288 for Borzoi. The corresponding embedding dimensions are 4096 (Evo2-7B), 256 (Caduceus-ph), 1024 (NT), 3072 (Enformer), and 1920 (Borzoi).

The model begins with a stack of four blocks, each implemented as a depthwise separable convolutional module. Each block consists of:

- A depthwise convolution followed by batch normalization and ReLU activation,
- A pointwise (1 × 1) convolution followed by batch normalization and ReLU activation
- An optional attention pooling.

The block parameters (kernel size, stride, padding, and pooling size) are:

- Block 1: (16, 8, 7, 2),
- Block 2: (8, 4, 3, 2),
- Block 3: (4, 2, 1, 2),
- Block 4: (3, 2, 1, 1; no pooling).

Following the convolutional blocks, a pointwise convolution projects the embedding dimension to 512. The tensor is then processed by a global average pooling layer (AdaptiveAvgPool1d(1)), reducing it to a fixed-length vector of 512 features per sample.

The final regression head consists of two fully connected layers with ReLU activation and dropout (0.5), producing a scalar corresponding to predicted gene expression. And the model processes both haplotypes independently and averages their predictions to produce a final output.

For the reference-only and individual-only training strategies, models were optimized using the Adam optimizer (Kingma and Ba, 2014) with an initial learning rate of 1e-4. A linear warm-up schedule was applied over the first 10% of training epochs, with a start factor of 0.01 and an end factor of 1.0, followed by a cosine scheduler for the remaining epochs. A total of 40 training epochs were used; no validation set was held out, and models from the final epoch were used for evaluation. For the combined reference and individual training strategy, models were first trained on reference-only data and then fine-tuned with individual genome-expression pairs for 40 epochs with the same configuration. As an additional sanity check, we performed a validation-based early-stopping experiment for rp-Caduceus using an independent validation split of 50 individuals for HLA-DQA1 (tolerance = 8 epochs), which yielded slightly lower but overall similar performance to the final-epoch checkpoint (Appendix Fig. S19). Experiments were conducted on NVIDIA H800 or L40S GPUs using a batch size of 4 or 8 and a random sampling strategy.

For reference-only training, we used Smooth L1 loss to optimize the models, where for individual-based training (including *p-* and *rp-* models), we defined a hybrid loss combining individual prediction accuracy with inter-sample consistency. The total loss ${{{\mathcal{L}}}}$ is computed as:

1 $${{{\mathcal{L}}}}=(1-\alpha )\cdot {{{{\mathcal{L}}}}}_{{{{\rm{ind}}}}}+\alpha \cdot {{{{\mathcal{L}}}}}_{{{{\rm{pair}}}}}$$

where:

- ${{{{\mathcal{L}}}}}_{{{{\rm{ind}}}}}$ is the smooth L1 loss between predicted and true gene expression values for each individual,
- ${{{{\mathcal{L}}}}}_{{{{\rm{pair}}}}}$ is a contrastive component that encourages the predicted pairwise differences to match the ground-truth differences across individuals and across genes,
- *α* ∈ [0, 1] is a weighting factor (set to 0.3 in this study).

Let ${\hat{y}}_{i}$ and *y*_*i*_ be the predicted and true expression values for individual *i*, respectively. The individual loss is defined as:

2 $${{{{\mathcal{L}}}}}_{{{{\rm{ind}}}}}=\frac{1}{N}{\sum}_{i=1}^{N}{{{\rm{Smooth}}}} {{{\rm{L1}}}}\,({\hat{y}}_{i},{y}_{i})$$

The pairwise contrastive loss is:

3 $${{{{\mathcal{L}}}}}_{{{{\rm{pair}}}}}=\frac{2}{N(N-1)}{\sum}_{i=1}^{N}{\sum}_{j=i+1}^{N}{{{\rm{Smooth}}}} {{{\rm{L1}}}}\,\left(\left\vert {\hat{y}}_{i}-{\hat{y}}_{j}\right\vert ,\left\vert {y}_{i}-{y}_{j}\right\vert \right)$$

where *N* is the batch size and Smooth L1( ⋅ , ⋅ ) denotes the Smooth L1 loss between two scalars.

### Predicting gene expression with S2F models

#### Predicting gene expression with the original Enformer and Borzoi

We used the pretrained Enformer via enformer-pytorch implementation (see Reagents and Tools Table) and Borzoi replicate-0 checkpoints from Hingerl et al (Hingerl et al, 2025), respectively. For each input sequence, we extracted the predicted track corresponding to CAGE:B lymphoblastoid cell line: GM12878 ENCODE, biol_. For Enformer, predictions were averaged across the central 10 output bins, as recommended in Huang et al (Huang et al, 2023), while for Borzoi, we averaged across the central 100 bins, which yielded optimal performance for this dataset. Both haplotypes of each individual were input separately, and their mean was used as the final gene expression prediction.

#### Predicting gene expression with AlphaGenome

We evaluated AlphaGenome via the publicly available Google API. For the gene expression prediction task, we performed an analysis by using an input length of 131,072 bp and tested two ontologies related to our benchmarking dataset: EFO:0000572 (lymphoblast tissue) and EFO:002784 (GM12878 lymphoblastoid cell line), together with five available output tracks. For each setting, predictions were averaged across the 1280 positions around the TSS, following the center-bin strategy used for Enformer. Both haplotypes for each individual were processed separately, and the final gene expression prediction was taken as their mean.

Performance across the different ontology-track combinations was comparable (see Appendix Table S2). We therefore selected EFO:002784 with the CAGE+ track for the main analysis, which has the optimal performance. Unless otherwise specified, results reported in the main text used this configuration with the maximum input length of 1,048,576 bp, in order to use the full receptive field of AlphaGenome.

For the chromatin accessibility analysis, we used AlphaGenome with an input length of 1,048,576 bp and selected the ATAC output track under EFO:002784, corresponding to the GM12878 lymphoblastoid cell line. Both haplotypes of each individual were processed separately, and their predicted ATAC signals were averaged. To obtain a gene-level predicted ATAC score, we summed the haplotype-averaged AlphaGenome ATAC signal across the annotated gene body. Observed ATAC signals were aggregated over the same gene-body regions using the matched ATAC-seq peak log-FPKM values. We then evaluated the concordance between AlphaGenome-predicted and observed chromatin accessibility by calculating Spearman’s *R* across individuals for each gene.

#### Fine-tuning S2F models

We compared full-parameter fine-tuning with using frozen embeddings of S2F models. Specifically, we fine-tuned the full Enformer and Borzoi models by replacing their original task-specific heads with a regression head. Backbone parameters were initialized using pretrained weights from enformer-pytorch implementation (see Reagents and Tools Table) and (Avsec et al, 2021; Hingerl et al, 2025; Linder et al, 2025). The new head included attention pooling applied to the central 10 output bins, yielding fixed-size embeddings of 3072 dimensions for Enformer and 1920 dimensions for Borzoi. These embeddings were passed through a fully connected linear layer to generate scalar predictions. Final gene expression values were obtained by averaging predictions across both haplotypes of each individual.

For the training strategies of full-parameter fine-tuning S2F models, we took the same training settings and the losses as described in *Predicting gene expression with gLM and S2F model embeddings*, except we set the initial learning rate to 1e-6.

### Predicting gene expression with PrediXcan

We implemented a PrediXcan-style baseline using the ElasticNetCV model from scikit-learn, with tenfold cross-validation, a fixed random state of 0, and all other parameters set to default. Separate models were trained for each gene using the same training and testing individuals as the sequence-based models, covering both 200 randomly selected training genes (results in Fig. 2A) and 287 held-out test genes. For each gene, we tested multiple window sizes and observed comparable results across them (Appendix Table S3). We therefore selected a 64 kb window centered on the TSS and extracted individual genotypes within this region using a minor allele frequency (MAF) threshold of 0.05.

We further used the PrediXcan-style model to estimate the *cis*-predictability of each gene. For this analysis, gene-specific ElasticNetCV models were trained using all available individuals, excluding the 100 held-out test individuals used for sequence-model evaluation. Model performance was then evaluated on the same 100 held-out individuals by calculating the Spearman *R* between predicted and observed gene expression. Genes were stratified according to their test Spearman R into four *cis*-predictability groups: low (*R* <0.1), weak (0.1≤*R* < 0.25), moderate (0.25≤*R* < 0.4), and high (*R*≥0.4).

### Variant-level perturbation analysis

For the exploratory variant-level analysis, we selected one representative gene, *ENSG00000230628*, from the set of genes for which the fitted linear model produced invariant predictions across individuals. For this gene, xDecoder-rp-Caduceus achieved a Spearman correlation of 0.385 between predicted and observed expression.

We evaluated all observed variants within the xDecoder genomic context window (*n* = 1893) for this gene. For each variant, we generated a matched perturbation sequence by reverting the variant allele to the reference allele while keeping the remaining sequence unchanged. The xDecoder perturbation score for variant *v* was defined as the absolute change in predicted expression after this reversion:

4 $${s}_{v}=\left\vert \Delta {\hat{y}}_{v}\right\vert =\left\vert f({S}_{v})-f({S}_{v\to {{{\rm{ref}}}}})\right\vert ,$$

where *S*_*v*_ denotes the sequence containing the observed variant and *S*_*v*→ref_ denotes the matched sequence in which variant *v* was reverted to the reference allele. Known eQTL SNPs were annotated using the eQTL results from the original study.

### Cross-ancestry performance analysis

To investigate factors associated with reduced cross-ancestry transferability, we compared the performance of rp-Caduceus models trained on European individuals and evaluated on held-out European and YRI individuals. For each gene, the cross-ancestry performance gap was defined as the difference in across-individual Spearman correlation between the European test set and the YRI test set:

5 $$\Delta R={R}_{{{{\rm{EUR}}}}}-{R}_{{{{\rm{YRI}}}}}.$$

A larger positive value therefore indicates a stronger reduction in predictive performance when transferring from European to YRI individuals.

All three indicators were calculated within the 131,070 bp rp-Caduceus input context centered on the TSS of each gene. First, we calculated the novel variant rate to quantify ancestry-specific variants within each gene region. Novel variants were defined as variants that were common in one population (MAF ≥ 0.05) but absent from the other population. The novel variant rate was defined as:

6 $${{{\rm{Novel}}}}\,{{{\rm{variant}}}}\,{{{\rm{rate}}}}=\frac{| {V}_{{{{\rm{EUR}}}}}\setminus {V}_{{{{\rm{YRI}}}}}| +| {V}_{{{{\rm{YRI}}}}}\setminus {V}_{{{{\rm{EUR}}}}}| }{| {V}_{{{{\rm{EUR}}}}}\cup {V}_{{{{\rm{YRI}}}}}| },$$

where *V*_EUR_ and *V*_YRI_ denote the sets of common variants observed in European and YRI individuals, respectively. Here, *V*_EUR_⧹*V*_YRI_ represents variants common in European individuals but absent from YRI individuals, whereas *V*_YRI_⧹*V*_EUR_ represents variants common in YRI individuals but absent from European individuals.

Second, we calculated the mean allele-frequency shift to measure population differences in variant frequency within the same region. For each retained variant, allele frequencies were calculated separately in European and YRI individuals. The gene-level allele-frequency shift was then defined as the mean absolute allele-frequency difference across all retained variants:

7 $${{{\rm{AF}}}}\,{{{\rm{shift}}}}=\frac{1}{| V| }{\sum}_{v\in V}\left\vert {{{{\rm{AF}}}}}_{v,{{{\rm{EUR}}}}}-{{{{\rm{AF}}}}}_{v,{{{\rm{YRI}}}}}\right\vert ,$$

where *V* = *V*_EUR_ ∪ *V*_YRI_.

Third, we estimated LD-structure similarity between European and YRI individuals. LD information was obtained using the Ensembl REST API linkage disequilibrium endpoint based on 1000 Genomes Project population data. For each gene region, we identified pairs of variants in strong linkage disequilibrium separately within each population, using an *R*^2^ > 0.8 threshold. The similarity of LD structure was quantified using the Jaccard index between the two sets of strong-LD variant pairs:

8 $${{{\rm{LD}}}}\,{{{\rm{similarity}}}}=\frac{| {L}_{{{{\rm{EUR}}}}}\cap {L}_{{{{\rm{YRI}}}}}| }{| {L}_{{{{\rm{EUR}}}}}\cup {L}_{{{{\rm{YRI}}}}}| },$$

where *L*_EUR_ and *L*_YRI_ represent the sets of variant pairs with *R*^2^ > 0.8 in European and YRI individuals, respectively. A higher Jaccard index indicates more similar local LD organization between populations, whereas a lower value indicates more population-specific LD structure.

### Predicting gene expression with ATAC inputs using xDecoder

To improve cross-individual gene expression prediction for unseen genes, we incorporated an additional channel of ATAC-seq peaks. Specifically, we obtained ATAC log-FPKM values for each peak from Kumasaka et al (2019) (Data ref: (Zenodo, 2019)). Among these, 94 samples overlapped with the Geuvadis consortium; 50 were used for training, and the remaining 44 for evaluation.

For DNA inputs, we used Caduceus as the embedding encoder with a sequence length of 64,000 bp centered on the TSS. For ATAC inputs, we converted peak-level log-FPKM values into base-resolution signals. The ATAC signal at base *i* was defined as:

9 $$s(i)=\left\{\begin{array}{ll}\sum\limits_{p\in {{{\mathcal{P}}}}(i)}\,{{\rm{log-FPKM}}}\,(p),\quad &\,{{\rm{if}}}\,| {{{\mathcal{P}}}}(i)| > 0\hfill\\ 0,\quad &\,{{\rm{if}}}\,| {{{\mathcal{P}}}}(i)| =0\end{array}\right.$$

where ${{{\mathcal{P}}}}(i)$ is the set of all peaks *p* that cover base *i* and *s*(*i*) is the ATAC signal at base *i*.

The ATAC peak channel was concatenated with the DNA embeddings, resulting in inputs of shape (sequence length × 257) (256 from DNA embeddings plus one ATAC channel). The downstream model architecture and training parameters were identical to those described in *Predicting gene expression with gLM and S2F model embeddings*.

Training strategies included reference-only, individual-only, and reference+individual settings. In the reference-only setup, ATAC signals were averaged across all 94 individuals to construct the reference channel.

### Predicting gene expression using ATAC-only inputs

We performed an ATAC-only control analysis to evaluate how much gene-expression variation could be explained by chromatin accessibility alone. This analysis was conducted under two settings: supervised prediction for the 200 seen genes used during individual-level training and unsupervised scoring for the 287 unseen genes used for zero-shot evaluation.

For the seen-gene setting, we formulated ATAC-only prediction as a supervised regression problem. For each gene, we extracted ATAC-seq peaks within a 64 kb window centered on the TSS, matching the input context used by the rp-Caduceus (DNA+ATAC) model. Peaks within this window were aggregated into five meta-peak features by summing log-FPKM ATAC values across meta-peaks. These features were then used to train a gene-specific PrediXcan-style linear model using ElasticNetCV from scikit-learn, with tenfold cross-validation, a fixed random state of 0, and all other parameters set to their default values.

For the unseen-gene setting, where no gene-specific supervised model was trained, we used a simple ATAC aggregation score as an unsupervised proxy for gene expression. Specifically, for each gene and individual, we extracted ATAC-seq peaks within the same 64 kb TSS-centered window and summed the log-FPKM peak values across the window. We then evaluated this ATAC-only score by calculating the Spearman R between the aggregated ATAC signal and the observed gene expression across individuals for each gene.

## Supplementary information

Appendix

Peer Review File

## Source data

Source data Fig. 1

Source data Fig. 2

Source data Fig. 3

Expanded View Figures

## Data Availability

The code produced in this study is available at https://github.com/StatBiomed/xDecoder. The source data of this paper are collected in the following database record: biostudies:S-SCDT-10_1038-S44320-026-00238-1.
