# Supplementary material for: xDecoder unlocks the potential of genomic foundation models for few-shot personal gene expression prediction
Source: Mol Syst Biol. 2026 Jul 28;22(11):1672–87. doi: 10.1038/s44320-026-00238-1 (PMC13728230; doi:10.1038/s44320-026-00238-1)
Supplement: Supplementary file 1 [file 44320_2026_238_MOESM1_ESM.pdf]

Appendix for xDecoder unlocks the potential of genomic foundation models for few-shot personal gene expression prediction

Table of Contents

Appendix Figure S1 Held-out chromosomes show similar cis-regulatory characteristics to the remaining chromosomes. 2
Appendix Figure S2 Across-unseen-gene correlation per unseen individual (Test 3 in Main.Fig. 1B). 3
Appendix Figure S3 Across-unseen-individual correlation per seen gene by different training strategies (Test 1 in Main.Fig. 1B 4
Appendix Figure S4 Distribution of Spearman R for across unseen individuals per seen gene. . . 5
Appendix Figure S5 Model performance for HLA-DQA1. 6
Appendix Figure S6 Model performance for METTL18. 7
Appendix Figure S7 Model performance for PPAN. 8
Appendix Figure S8 Spearman correlations of r-Caduceus and rp-Caduceus across unseen individuals for seen genes. 9
Appendix Figure S9 Association between across-unseen-individual performance per seen gene and p-values of eQTL SNP. 10
Appendix Figure S10 Impact of INDEL burden on model performance. 11
Appendix Figure S11 Distribution of Spearman R for across unseen individuals per unseen gene (task 3). 12
Appendix Figure S12 Cis-predictability stratifies zero-shot performance on unseen genes. 13
Appendix Figure S13 Fine-tuning vs. frozen-representation performance of S2F models across three evaluation tasks. 14
Appendix Figure S14 Comparison of model performance when restricting the receptive field to 64 kb versus using the full context size. 15
Appendix Figure S15 Incorporating ATAC-seq signals improves across-unseen-individual prediction for unseen genes. 16
Appendix Figure S16 Model performance for the unseen gene of SLC12A7. 17
Appendix Figure S17 Model performance for the unseen gene of KIAA1217. 18
Appendix Figure S18 Comparison of AlphaGenome performance on gene expression and gene-body ATAC for unseen genes. 19
Appendix Figure S19 Comparison between checkpoints from early-stopped and last epoch. . . . 20

Appendix Table S1 Gene expression prediction performance (mean Spearman R) across different training strategies for xDecoder with one-hot embeddings (context length: 131,070 bp). 21
Appendix Table S2 Performance comparison of AlphaGenome across ontology and track settings. 22
Appendix Table S3 Performance of PrediXcan under different receptive field sizes for the across-unseen-individual, per seen gene prediction task. 23

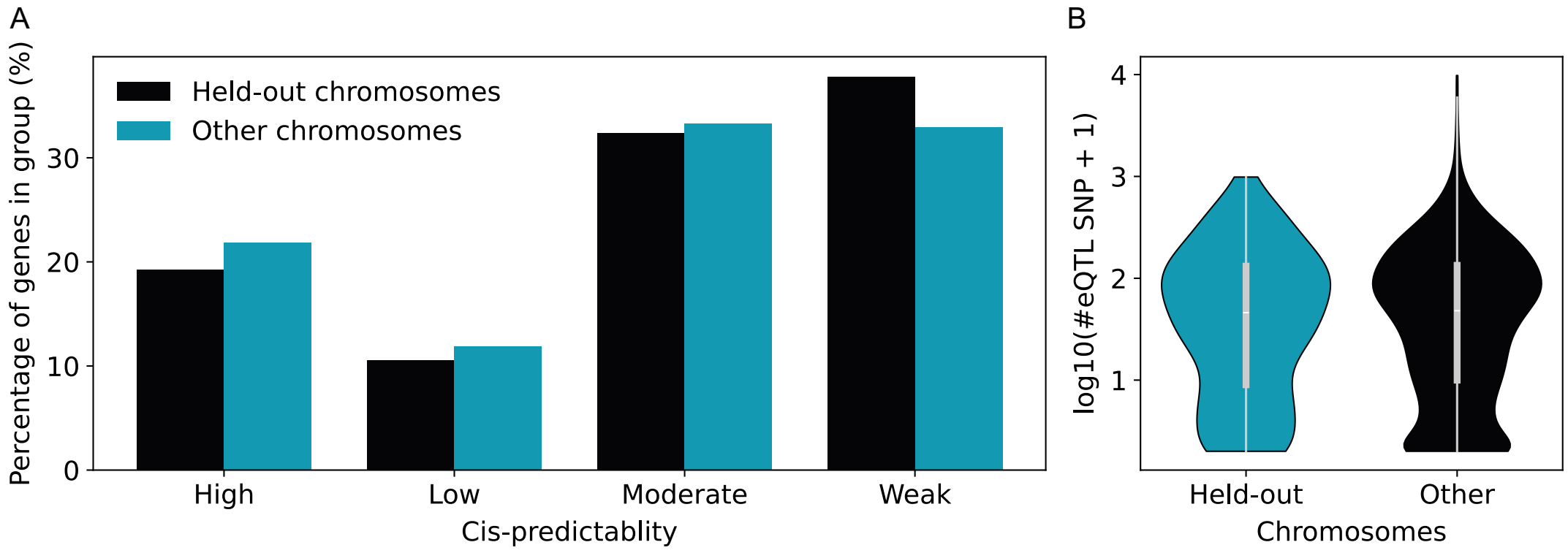

Appendix Figure S1 Held-out chromosomes show similar cis-regulatory characteristics to the remaining chromosomes. A, Percentage of genes in each cis-predictability category (low, weak, moderate, and high) for genes on the held-out chromosomes (chromosomes 5 and 10) and the remaining chromosomes. Cis-predictability was estimated using a PrediXcan-based linear model. B, Distribution of the number of significant cis-eQTL SNPs per gene, shown as log10(eQTL SNP + 1), for genes on the held-out chromosomes and the remaining chromosomes.

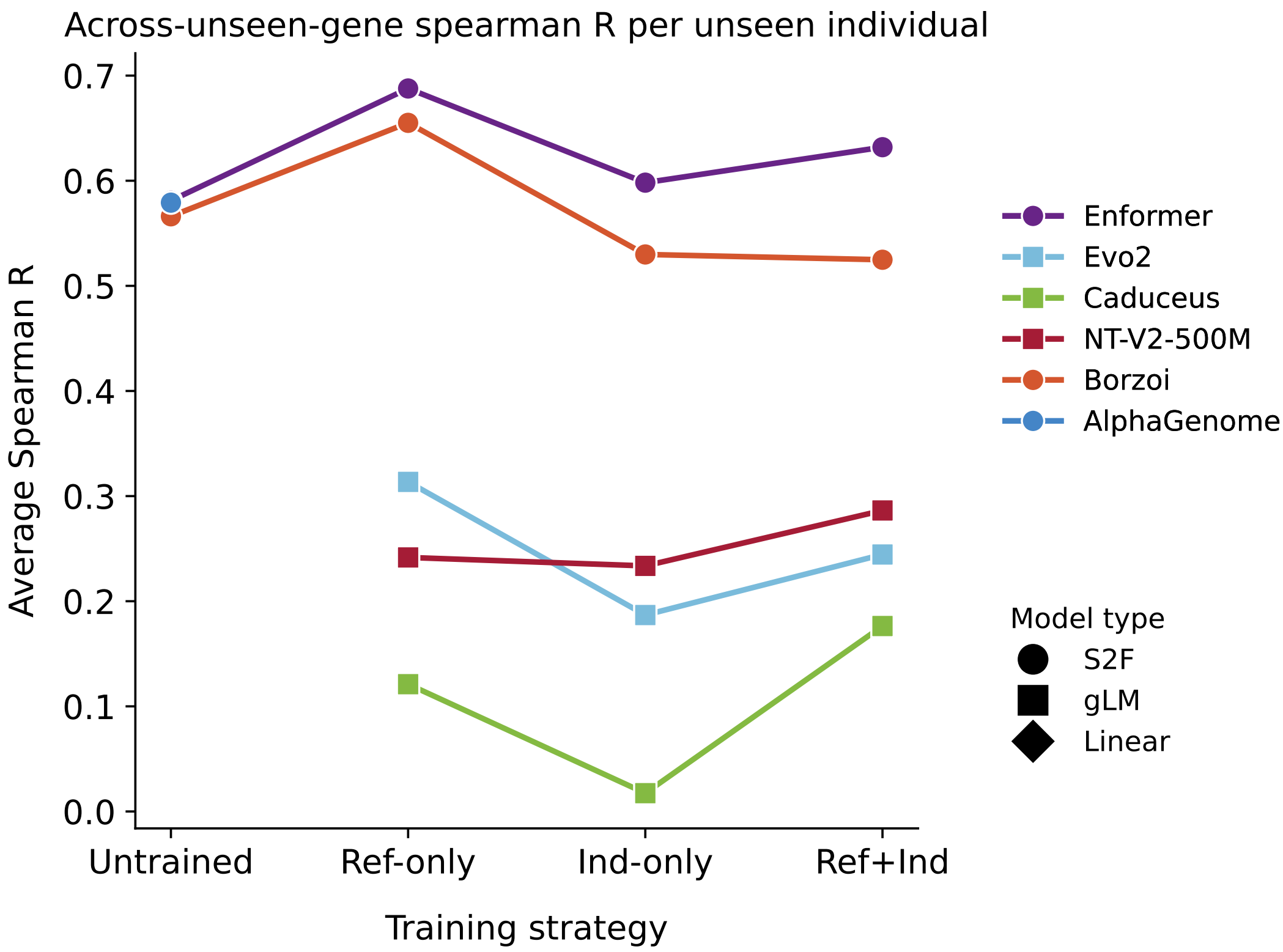

Appendix Figure S2 Across-unseen-gene correlation per unseen individual (Test 3 in Main.Fig. 1B).

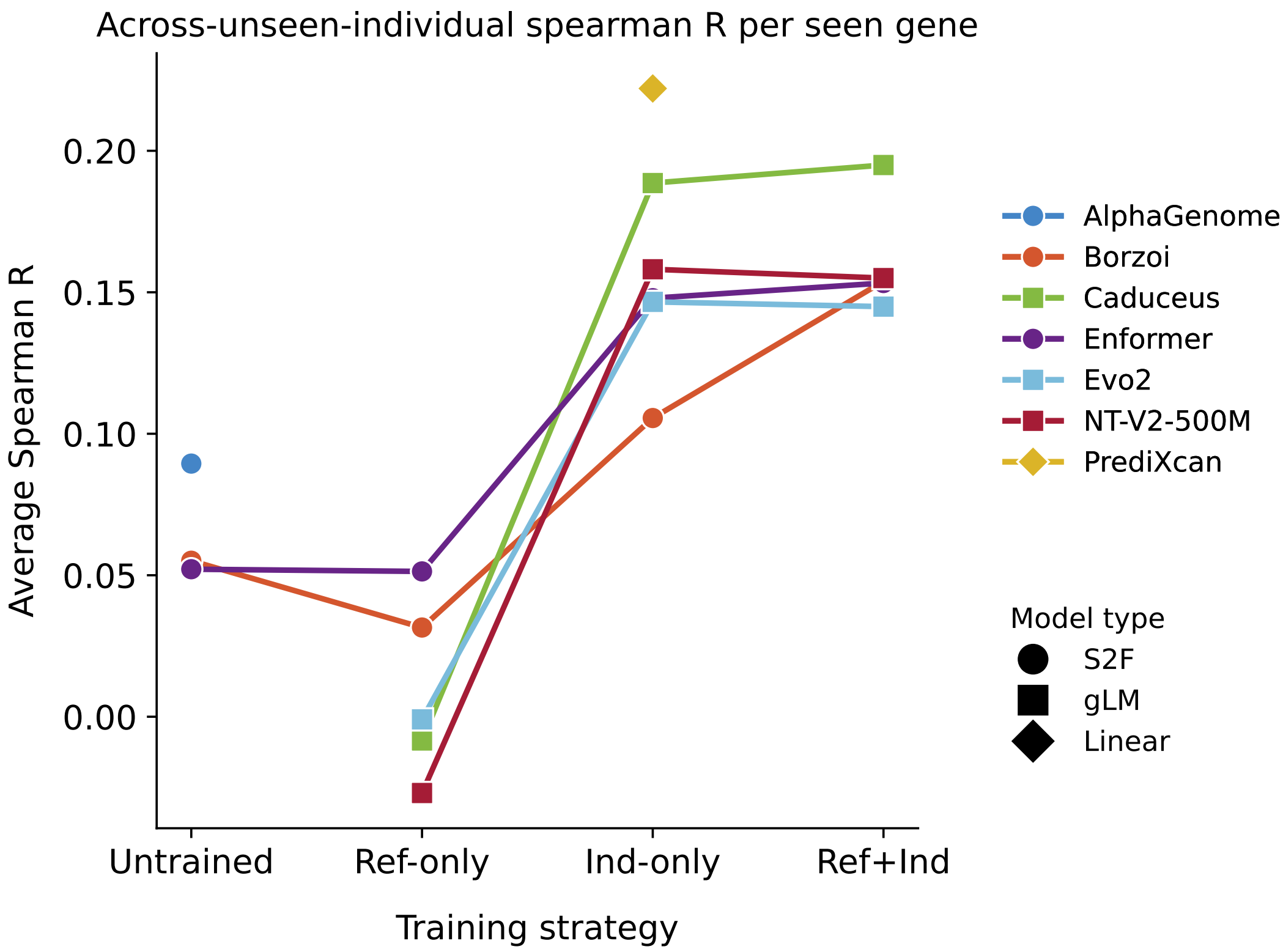

Appendix Figure S3 Across-unseen-individual correlation per seen gene by different training strategies (Test 1 in Main.Fig. 1B).

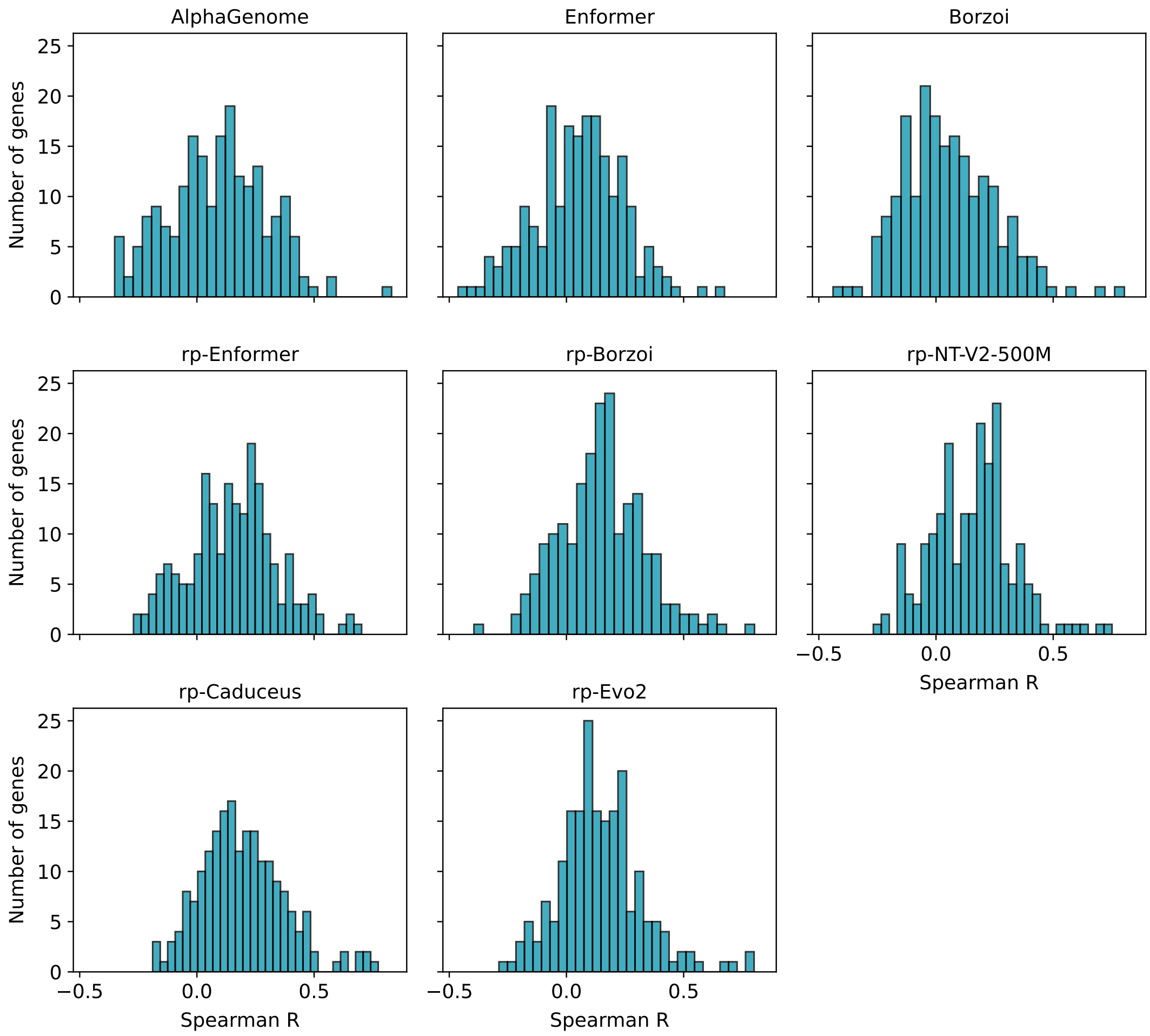

Appendix Figure S4 Distribution of Spearman R for across unseen individuals per seen gene

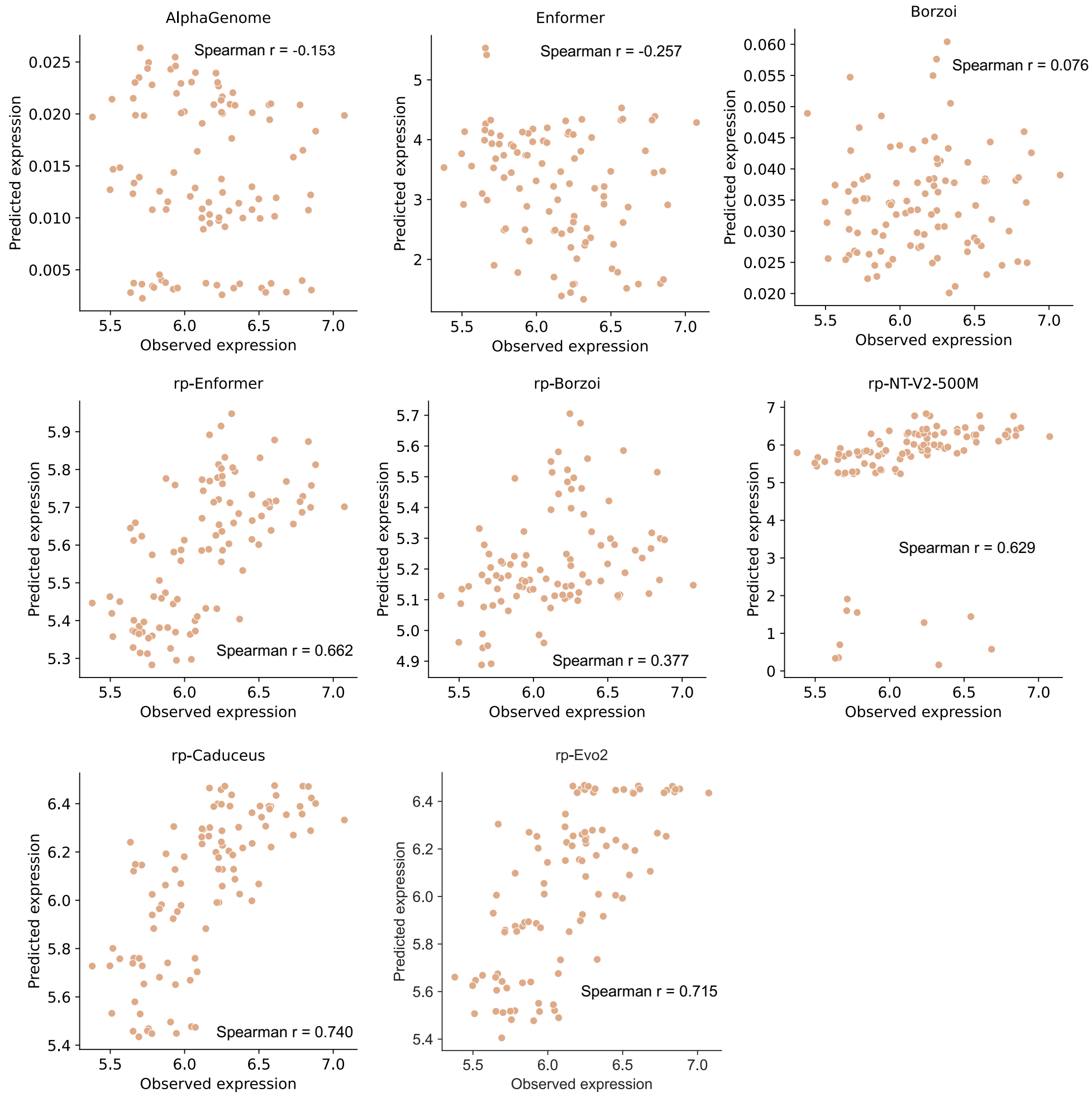

Appendix Figure S5 Model performance for HLA-DQA1. The prefix "rp-" indicates models that were pretrained on 2,972 genes from the reference genome and subsequently fine-tuned using individual-level data from 50 individuals, focusing on 200 random genes.

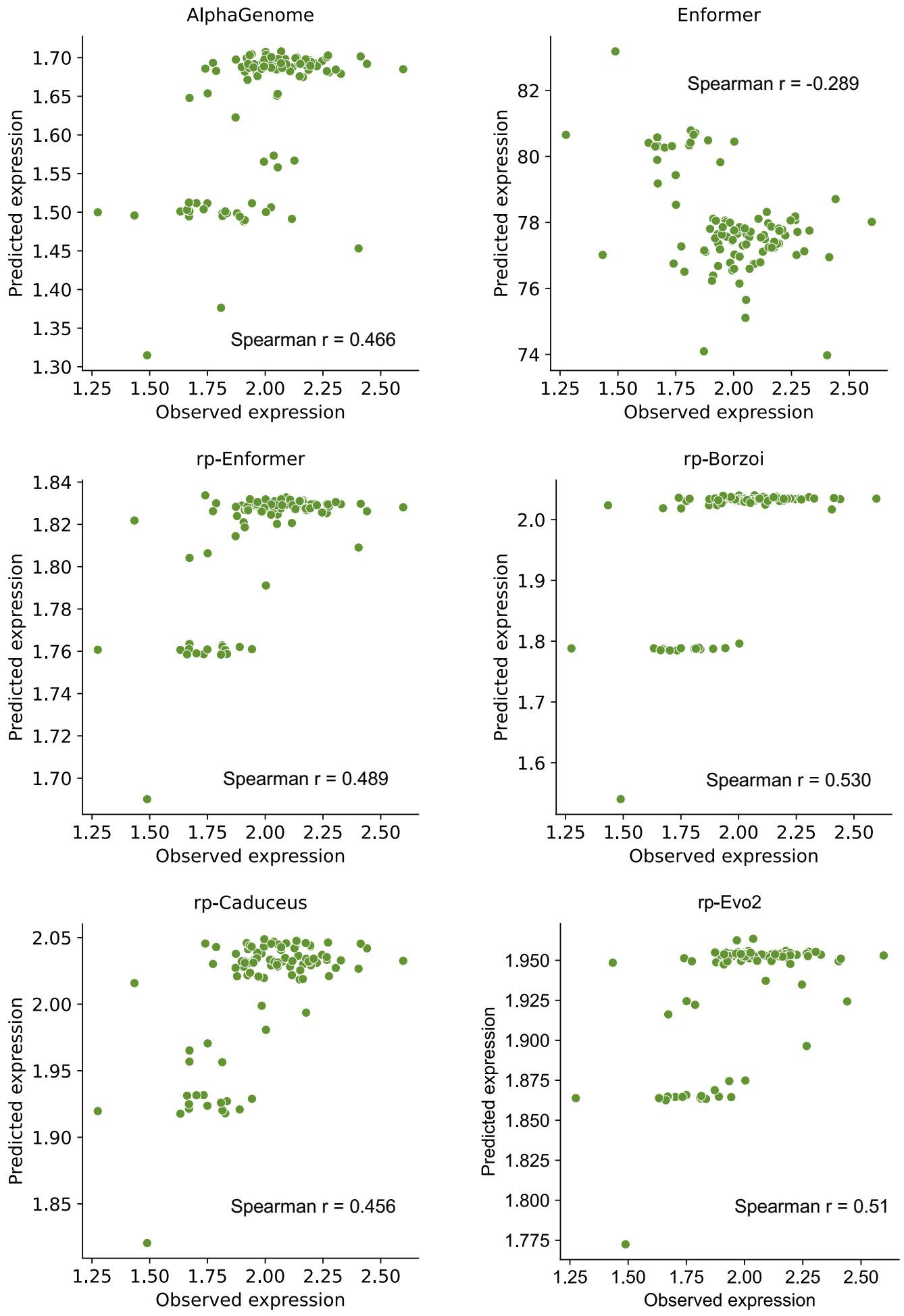

Appendix Figure S6 Model performance for METTL18. The prefix "rp-" indicates models that were pretrained on 2,972 genes from the reference genome and subsequently fine-tuned using individual-level data from 50 individuals, focusing on 200 random genes.

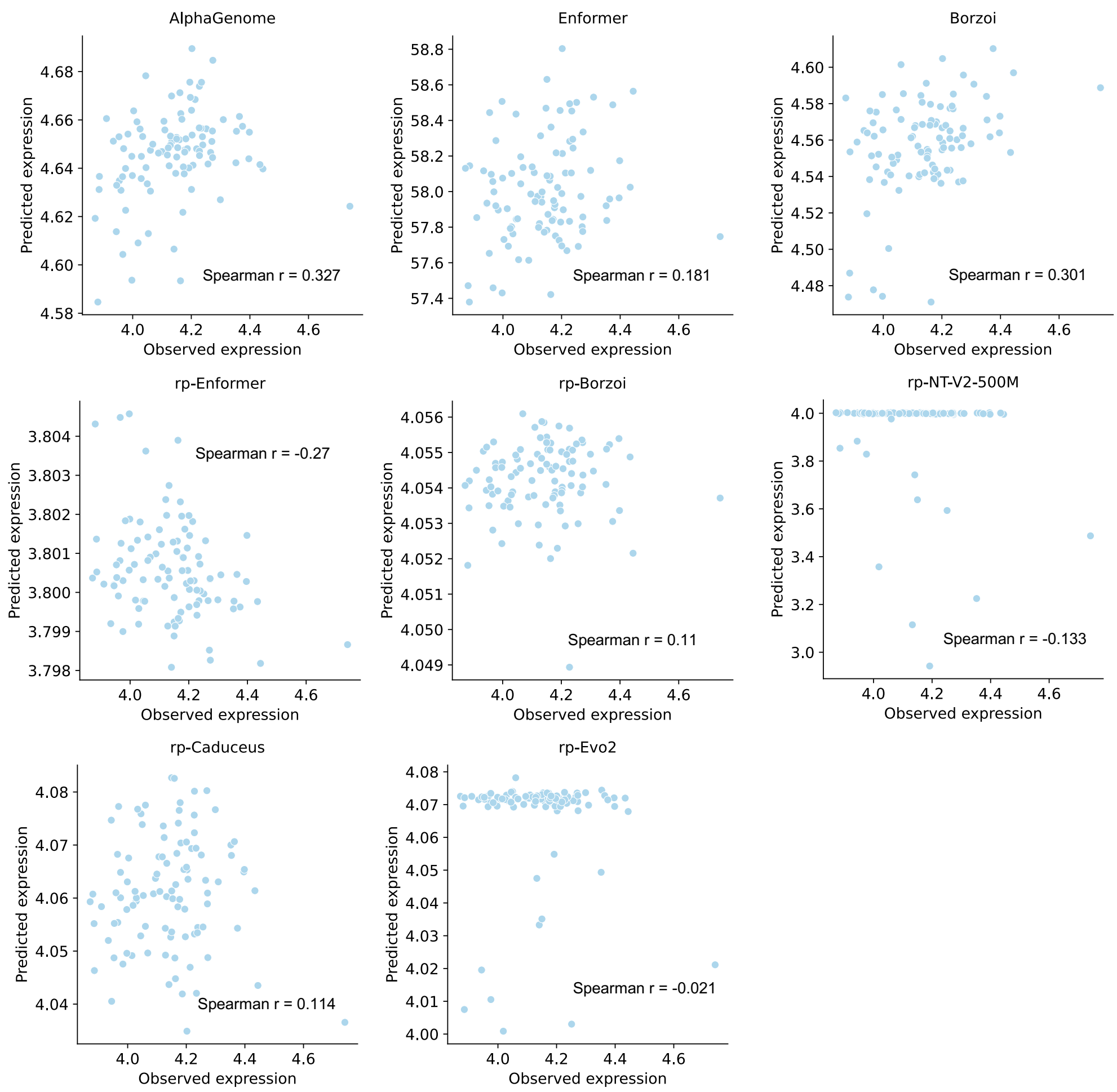

Appendix Figure S7 Model performance for PPAN. The prefix "rp-" indicates models that were pretrained on 2,972 genes from the reference genome and subsequently fine-tuned using individual-level data from 50 individuals, focusing on 200 random genes.

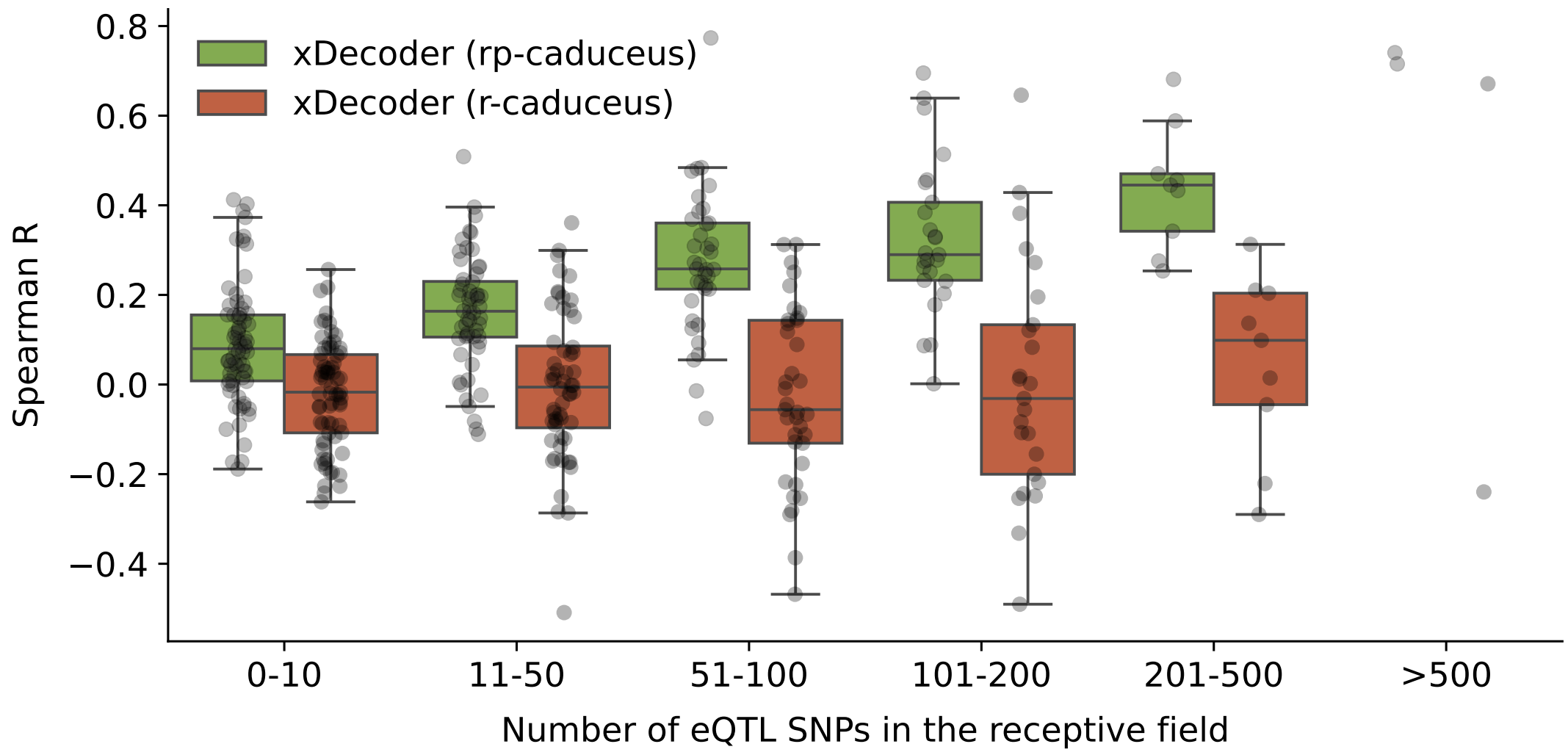

Appendix Figure S8 Spearman correlations of r-Caduceus and rp-Caduceus across unseen individuals for seen genes. Genes were binned by the number of eQTL SNPs in the receptive field.

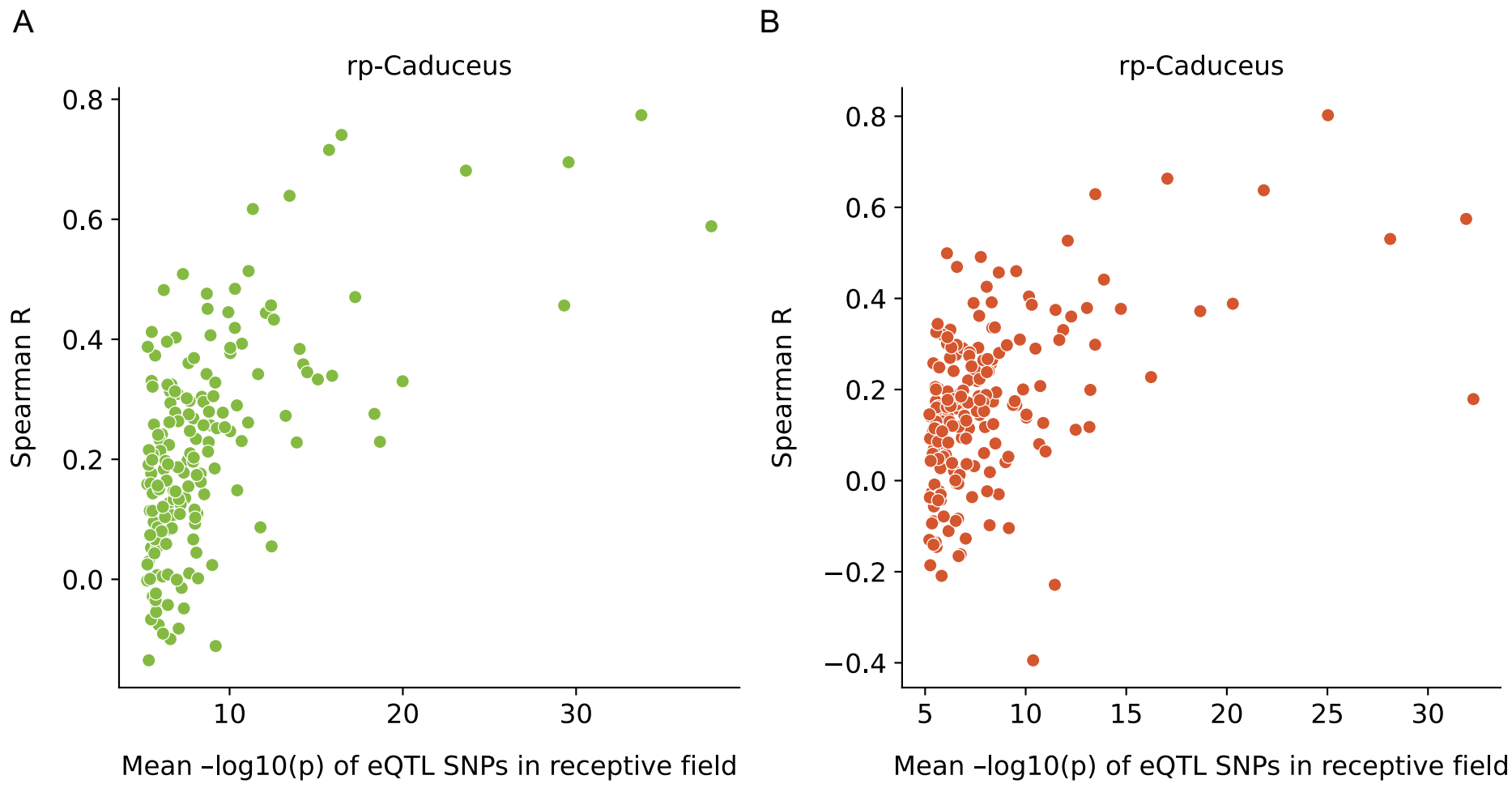

Appendix Figure S9 Association between across-unseen-individual performance per seen gene and p-values of eQTL SNP. A, Mean –log10(p) of eQTL SNPs in the receptive field versus Spearman R for rp-Caduceus (175 genes with at least one eQTL SNP within the receptive field were included). B, Mean –log10(p) of eQTL SNPs in the receptive field versus Spearman R for rp-Borzoi (190 genes with at least one eQTL SNP within the receptive field were included).

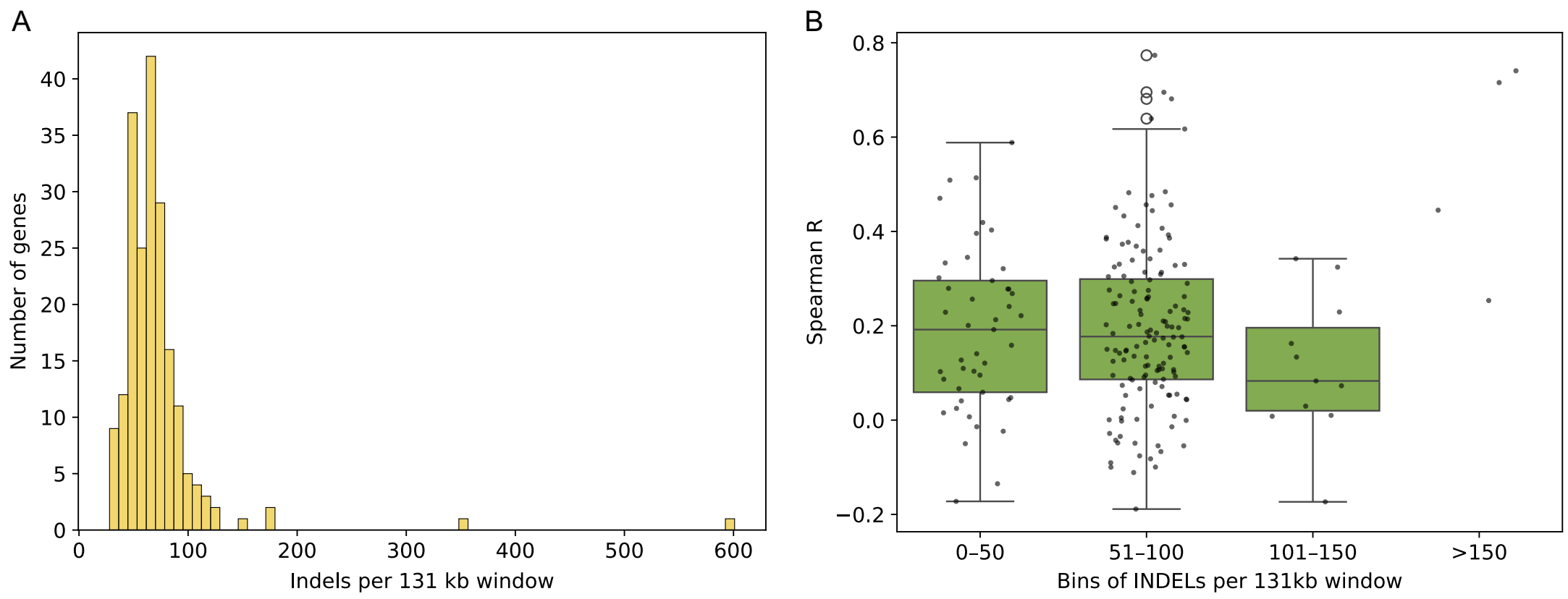

Appendix Figure S10 Impact of INDEL burden on model performance. (A) Distribution of the number of indels within 131,070 bp windows around the TSS for the 200 seen genes (Test 1, main Fig. 1B). (B) Performance of xDecoder (rp-Caduceus) binned by the number of indels in the 131,070 bp context around the TSS.

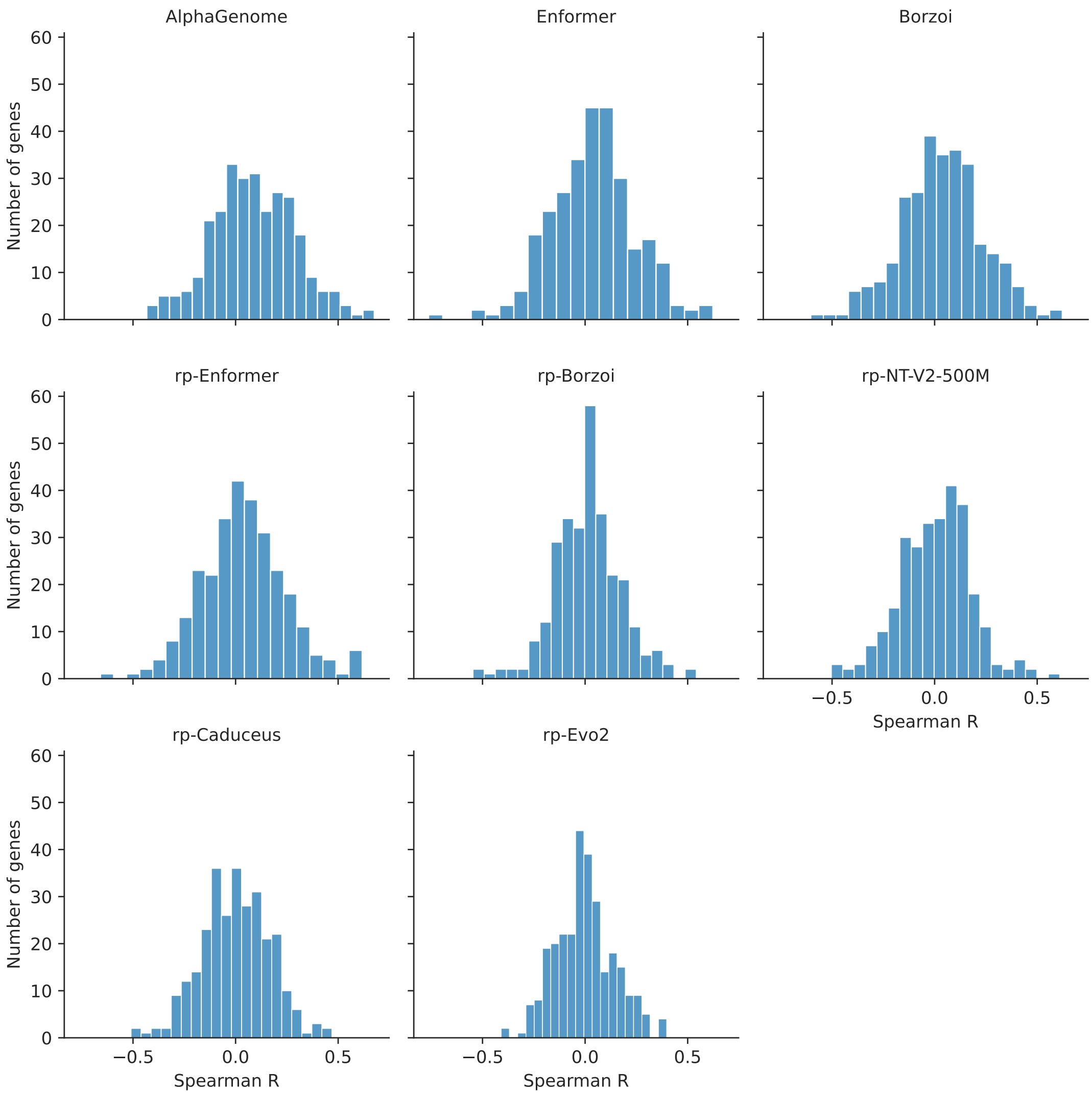

Appendix Figure S11 Distribution of Spearman R for across unseen individuals per unseen gene (task 3).

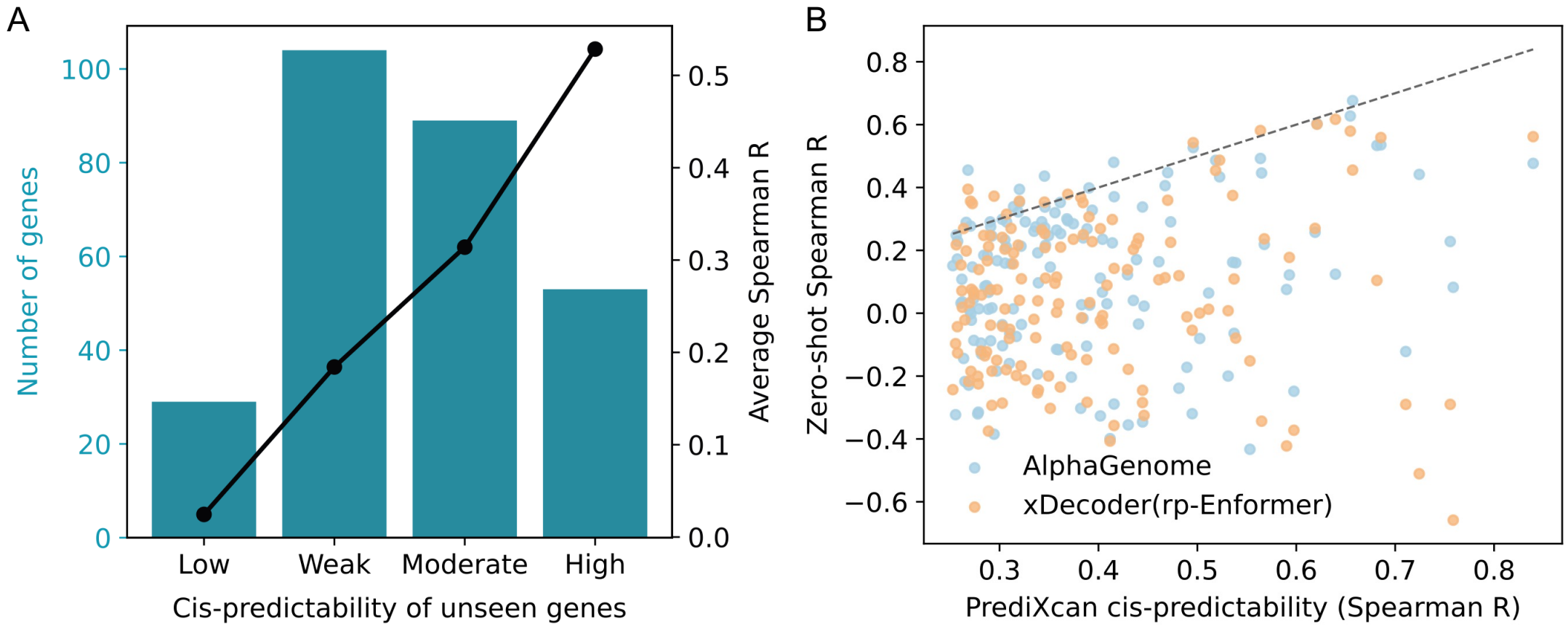

Appendix Figure S12 Cis-predictability stratifies zero-shot performance on unseen genes. A, Distribution of unseen genes across PrediXcan cis-predictability groups and the mean PrediXcan performance within each group. PrediXcan cis-predictability was estimated using a gene-specific ElasticNet model trained on common cis variants (MAF ≥ 0.05) within a 131 kb window centered on the TSS, using all available training individuals ($n = 362$), and evaluated on 100 testing individuals. Groups are defined as low ($R < 0.10$), weak ($0.10 \leq R < 0.25$), moderate ($0.25 \leq R < 0.40$), and high ($R \geq 0.40$). B, Gene-level relationship between PrediXcan cis-predictability and zero-shot performance of AlphaGenome and xDecoder(rp-Enformer), measured by Spearman R, for unseen genes with moderate or high cis-predictability.

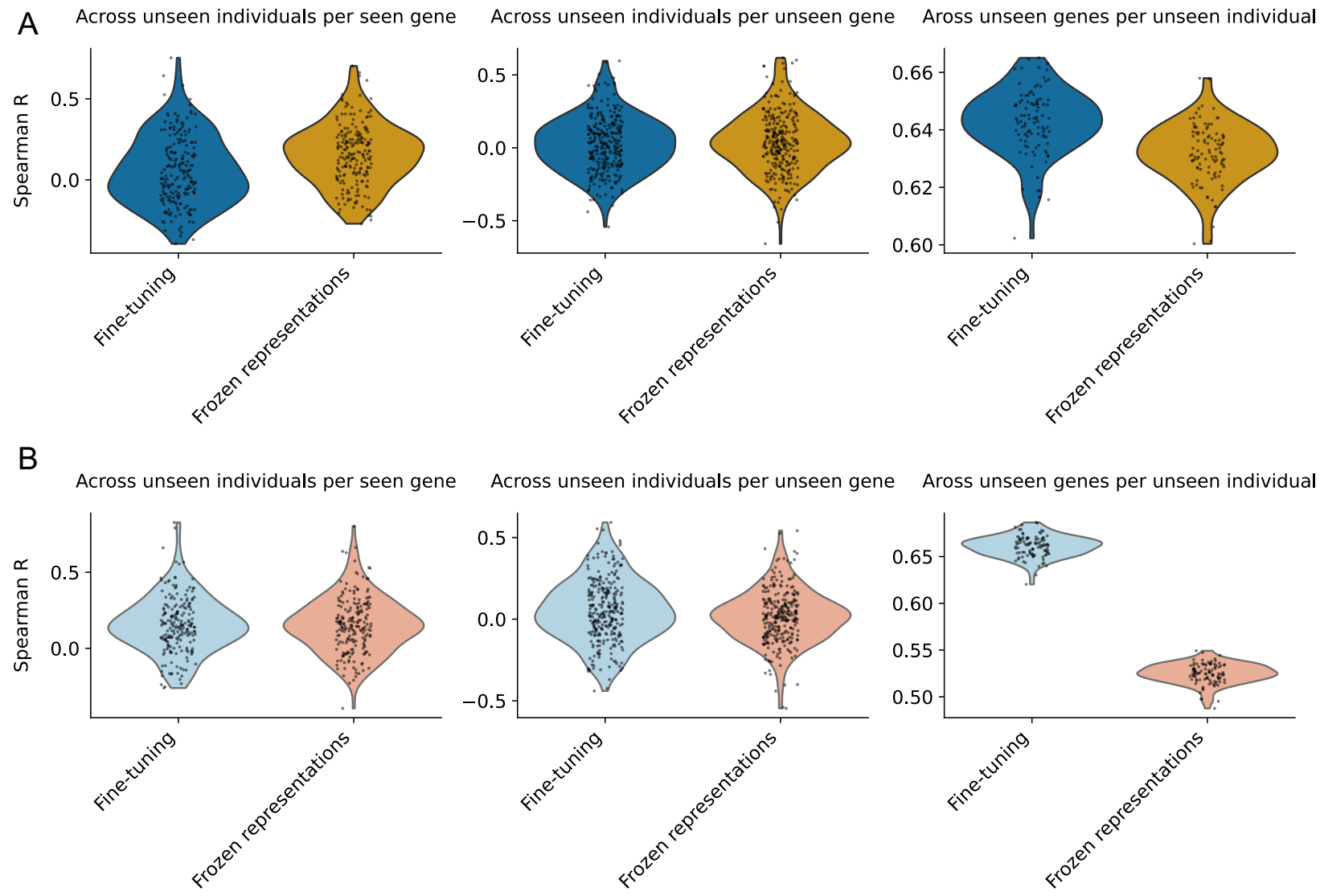

Appendix Figure S13 Fine-tuning vs. frozen-representation performance of S2F models across three evaluation tasks. All models were pretrained on the reference genome using 2,972 genes, then trained with individual-level data from 50 individuals on 200 randomly selected genes. Evaluation used 100 held-out individuals with 200 seen genes and 287 unseen genes.(A) Enformer (full receptive field: 196 kb). (B) Borzoi (full receptive field: 524 kb). Columns, left to right: across unseen individuals for seen genes; across unseen individuals for unseen genes; across unseen genes for unseen individuals.

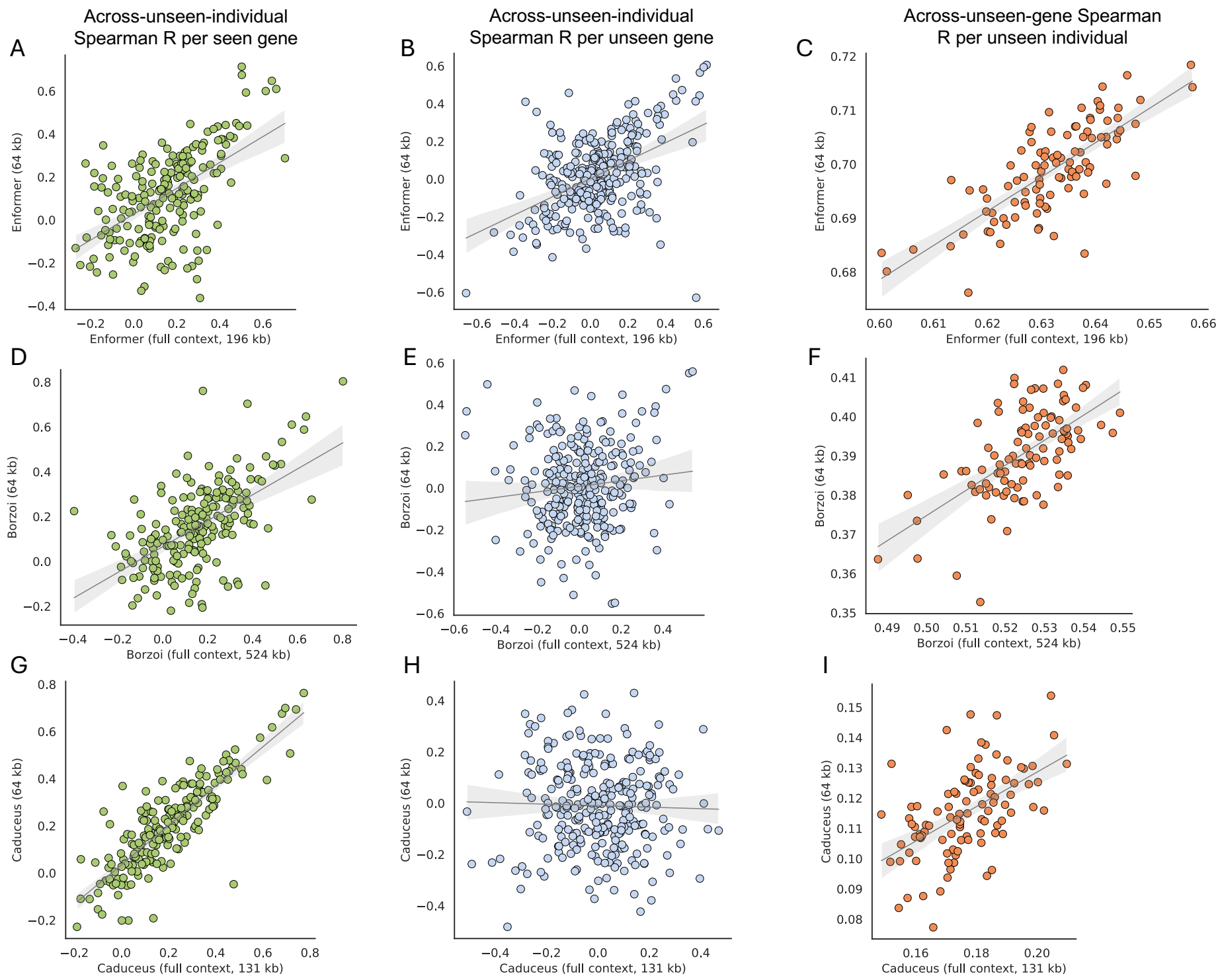

Appendix Figure S14 Comparison of model performance when restricting the receptive field to 64 kb versus using the full context size. A–C, Enformer (full context 196 kb vs. 64 kb) across tasks: (A) across-unseen-individual correlation per seen gene, (B) across-unseen-individual correlation per unseen gene, and (C) across-unseen-gene correlation per unseen individual. D–F, Borzoi (full context 524 kb vs. 64 kb) across the same tasks. G–I, Caduceus (full context 131 kb vs. 64 kb) across the same tasks. Points represent genes.

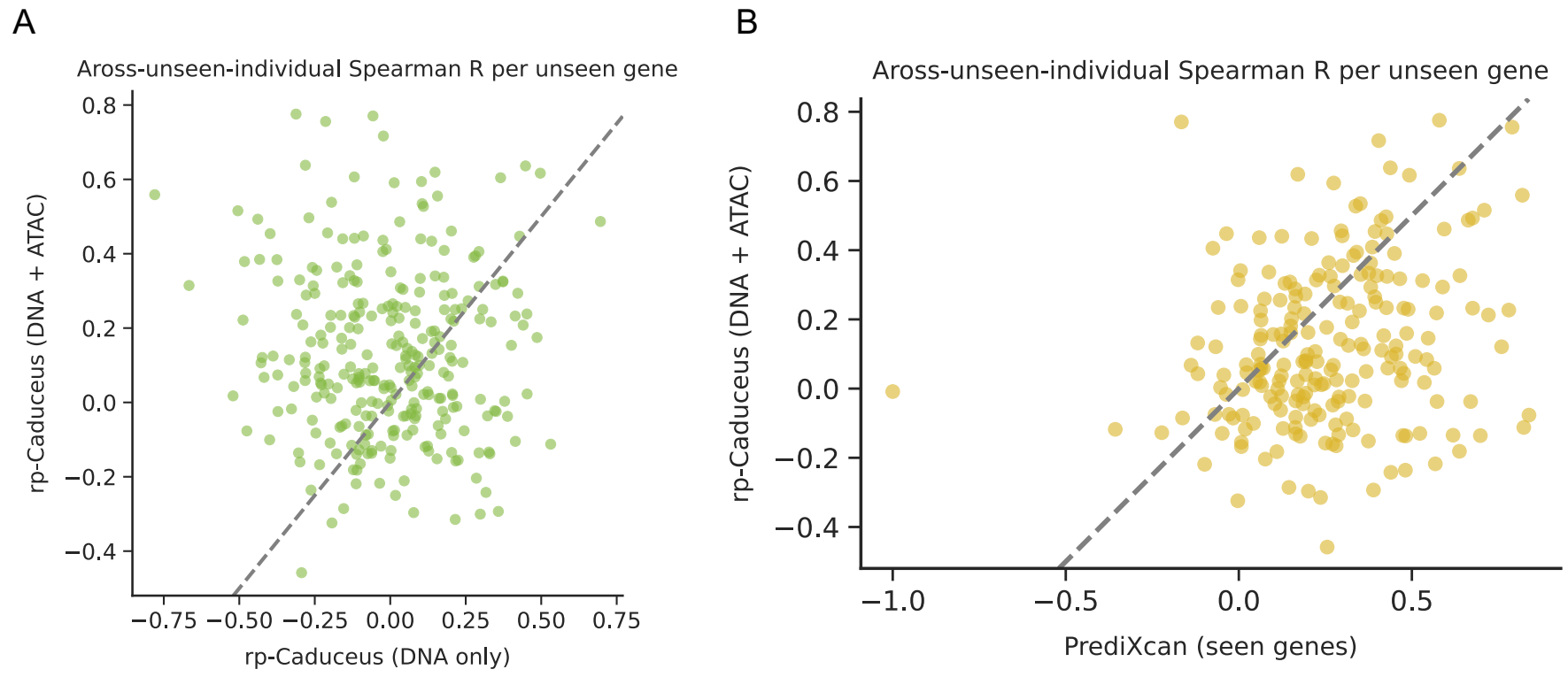

Appendix Figure S15 Incorporating ATAC-seq signals improves across-unseen-individual prediction for unseen genes. (A) Gene-wise comparison of across-unseen-individual Spearman correlations between DNA-only and DNA+ATAC rp-Caduceus models. Many genes show improved correlation with ATAC inputs. (B) Gene-wise comparison of DNA+ATAC predictions with PrediXcan predictions (seen genes).

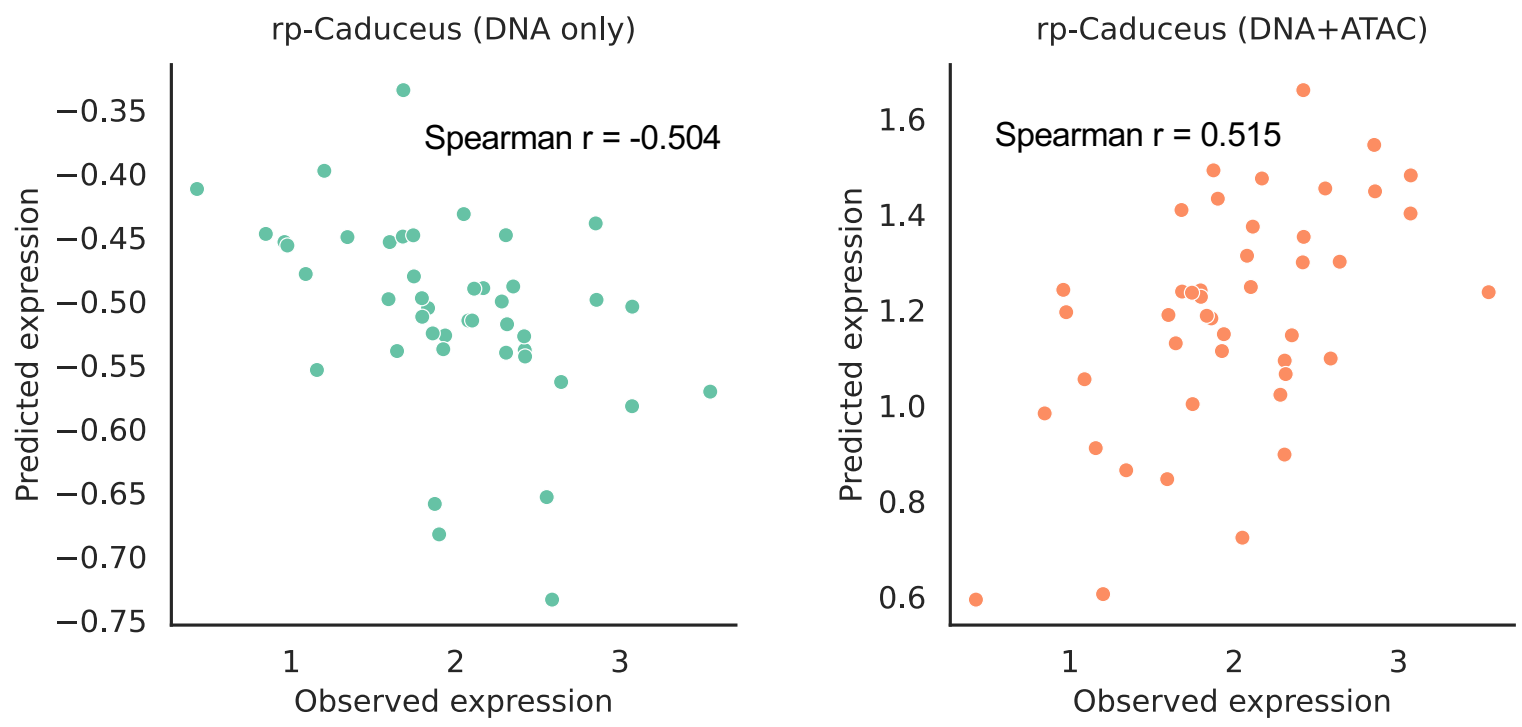

Appendix Figure S16 Model performance for the unseen gene of SLC12A7. The prefix "rp-" indicates models that were pretrained on 2,972 genes from the reference genome and subsequently fine-tuned using individual-level data from 50 individuals, focusing on 200 random genes.

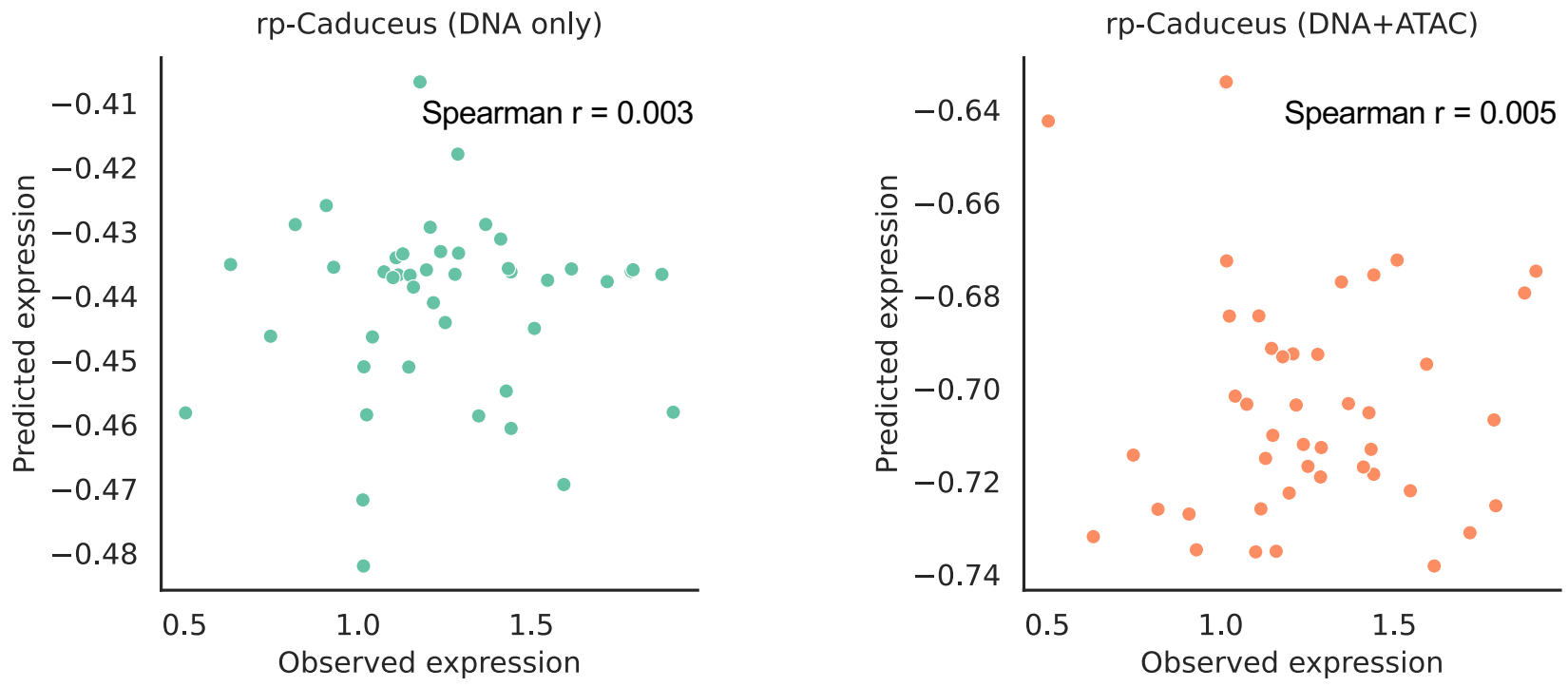

Appendix Figure S17 Model performance for the unseen gene of KIAA1217. The prefix "rp-" indicates models that were pretrained on 2,972 genes from the reference genome and subsequently fine-tuned using individual-level data from 50 individuals, focusing on 200 random genes.

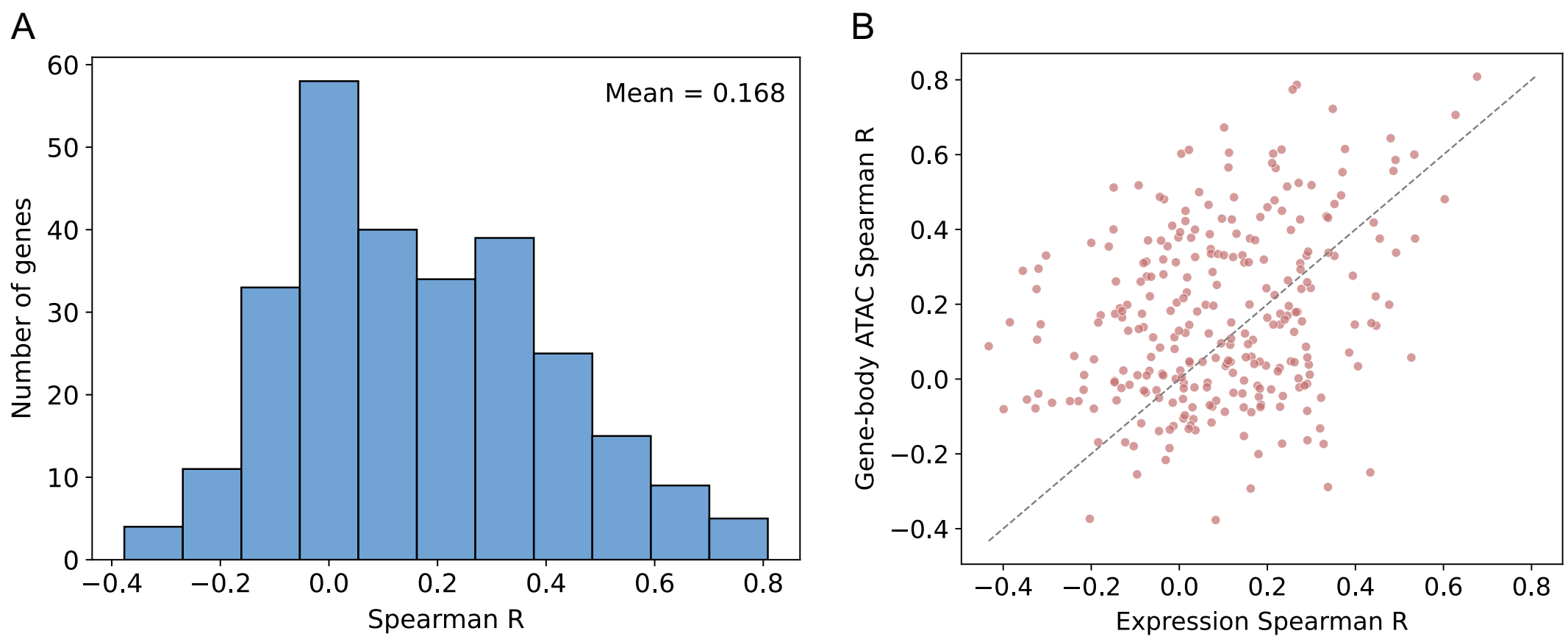

Appendix Figure S18 Comparison of AlphaGenome performance on gene expression and gene-body ATAC for unseen genes. AlphaGenome was evaluated on held-out unseen genes in a zero-shot setting (Test 3 in Main Fig. 1B). For each gene, predicted and observed gene-body ATAC signals were obtained by summing ATAC signal within the gene body for each individual, through the Spearman correlation across individuals. Gene-wise Spearman correlations across individuals were also obtained for predicted versus observed gene expression. (A) Distribution of gene-wise gene-body ATAC correlations. (B) Scatter plot comparing gene-wise expression correlation and gene-wise gene-body ATAC correlation; each dot represents one gene.

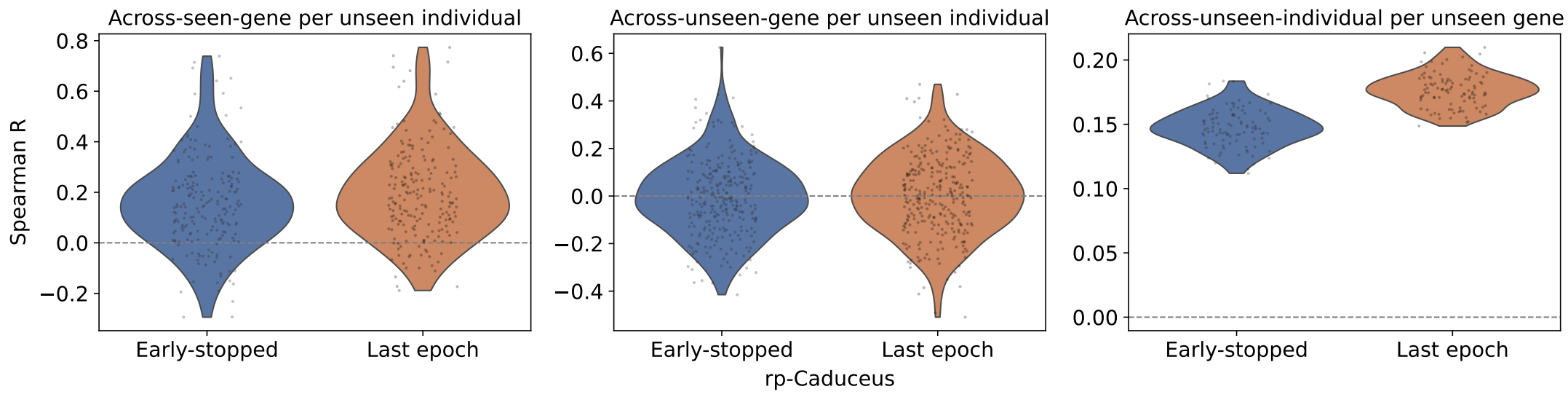

Appendix Figure S19 Comparison between checkpoints from early-stopped and last epoch. Held-out test performance of xDecoder rp-Caduceus under two checkpoint-selection strategies: final-epoch checkpoint (40 epochs) and validation-based early stopping (tolerance = 8 epochs). Early stopping was determined using an independent validation split of 50 individuals for HLA-DQA1.

Supplementary Tables

Appendix Table S1 Gene expression prediction performance (mean Spearman R) across different training strategies for xDecoder with one-hot embeddings (context length: 131,070 bp). Task 1: across unseen individuals per seen gene (Test set 1 in Fig. 1B); Task 2: across unseen individuals per unseen gene (Test set 3 in Fig. 1B); Task 3: across unseen genes per unseen individual (Test set 3 in Fig. 1B).

**Training strategy**	**Task 1**	**Task 2**	**Task 3**
Ref-only	-0.016	0.0005	-0.015
Indiv-only	0.023	0.006	-0.047
Ref + indiv	0.003	0.006	0.007

Appendix Table S2 Performance comparison of AlphaGenome across ontology and track settings. Task1: Across unseen individuals per seen gene (Test set 1 in Main Fig. 1B); Task2: Across unseen individuals per unseen gene (Test set 3 in Main Fig. 1B); Task3: Across unseen genes per unseen individual (Test set 3 in Main Fig. 1B).

		**Mean Spearman R**		
**Ontology**	**Track**	**Task1**	**Task2**	**Task3**
EFO:0000572	RNA-seq plus PolyA	0.073	0.086	0.563
EFO:002784	CAGE +	0.083	0.079	0.608
	RNA-seq plus PolyA .	0.073	0.061	0.512
	RNA-seq plus PolyA +	0.070	0.080	0.578
	Total RNA-seq +	0.073	0.075	0.575

Appendix Table S3 Performance of PrediXcan under different receptive field sizes for the across-unseen-individual, per seen gene prediction task. Non-predictable genes are defined as genes with identical predicted expression levels across individuals.

Receptive field (bp)	Mean Spearman R	Number of non-predictable genes
12,288	0.220	51
64,000	0.222	46
131,071	0.228	53
196,608	0.220	55
524,288	0.164	54
1,048,576	0.166	68
