# Supplementary material for: xDecoder unlocks the potential of genomic foundation models for few-shot personal gene expression prediction
Source: Mol Syst Biol. 2026 Jul 28;22(11):1672–87. doi: 10.1038/s44320-026-00238-1 (PMC13728230; doi:10.1038/s44320-026-00238-1)
Supplement: Supplementary file 2 [file 44320_2026_238_MOESM2_ESM.pdf]

molecular systems biology

xDecoder unlocks the potential of genomic foundation models for few-shot personal gene expression prediction

Shumin Li, Ruibang Luo, and Yuanhua Huang

Corresponding author(s): Yuanhua Huang (yuanhua@hku.hk) , Ruibang Luo (rbluo@cs.hku.hk)

Review Timeline:

Submission Date:	23rd Dec 25
Editorial Decision:	26th Feb 26
Revision Received:	8th May 26
Editorial Decision:	24th Jun 26
Revision Received:	9th Jul 26
Accepted:	14th Jul 26

Editor: Poonam Bheda

Transaction Report:

(Note: With the exception of the correction of typographical or spelling errors that could be a source of ambiguity, letters and reports are not edited. Depending on transfer agreements, referee reports obtained elsewhere may or may not be included in this compilation. Referee reports are anonymous unless the Referee chooses to sign their reports.)

26th Feb 2026

Manuscript Number: MSB-2025-13544
Title: xDecoder unlocks the potential of genomic foundation models for few-shot personal gene expression prediction

Dear Dr. Huang,

Thank you again for submitting your work to Molecular Systems Biology. We have now heard back from the three reviewers who agreed to evaluate your study. As you will see below, the reviewers appreciate that the proposed approach addresses a timely topic. However, they raise a series of concerns, which we would ask you to address in a major revision.

Without repeating all the comments listed below, some of the more fundamental issues raised are the following:
- ATAC improvements to the model are not sufficiently investigated. Additional evaluation on whether there is indeed an interaction of this information with DNA sequence alone or rather that ATAC data are inherently correlated with gene expression should be performed.
- Unclear whether the improvements showcased are for specific genes or more generalizable. This should be further investigated and/or presented more clearly.

All other issues raised would need to be satisfactorily addressed. Please let me know in case you would like to discuss in further detail any of the comments, I would be happy to schedule a call.

When submitting your revised manuscript, please carefully review the instructions that follow below. We perform an initial quality control of all revised manuscripts before re-review; failure to include requested items will delay the evaluation of your revision.

We require:

1) A .docx formatted version of the manuscript text (including legends for main figures, EV figures and tables). Please make sure that the changes are highlighted to be clearly visible. Alternatively you may choose to submit your manuscript as a LaTeX file.

2) Individual production quality figure files as .eps, .tif, .jpg (one file per figure). For guidance, download the 'Figure Guide PDF' (https://media.springernature.com/original/springer-cms/rest/v1/content/27825798/data/v1).

3) At EMBO Press we ask authors to provide source data for the main figures. Our source data coordinator will contact you to discuss which figure panels we would need source data for and will also provide you with helpful tips on how to upload and organize the files.

4) A .docx formatted letter INCLUDING the reviewers' reports and your detailed point-by-point responses to their comments. As part of the EMBO Press transparent editorial process, the point-by-point response is part of the Peer Review File (PRF), which will be published alongside your paper.

5) A complete author checklist, which you can download from our author guidelines (https://link.springer.com/journal/44320/submission-guidelines#cms). Please insert information in the checklist that is also reflected in the manuscript. The completed author checklist will also be part of the PRF.

6) Please note that all corresponding authors are required to supply an ORCID ID for their name upon submission of a revised manuscript.

7) It is mandatory to include a 'Data Availability' section after the Materials and Methods. Before submitting your revision, primary datasets produced in this study need to be deposited in an appropriate public database, and the accession numbers and database listed under 'Data Availability'. Please remember to provide a reviewer password if the datasets are not yet public (see https://link.springer.com/partners/embo-press/editorial-policies#Data%20availability%20statement).

In case you have no data that requires deposition in a public database, please state so in this section as follows: "This study includes no data deposited in external repositories". Note that the Data Availability Section is restricted to new primary data that are part of this study.

8) All Materials and Methods need to be described in the main text using our 'Structured Methods' format, which is required for all research articles. According to this format, the Methods section includes a Reagents and Tools Table (listing key reagents, experimental models, software and relevant equipment and including their sources and relevant identifiers) followed by a Methods and Protocols section describing the methods using a step-by-step protocol format. The aim is to facilitate adoption of the methodologies across labs. Please upload the Reagents and Tools table as a separate document when submitting your revised manuscript. More information on how to adhere to this format as well as a downloadable template (.docx) for the

Reagents and Tools Table can be found in our author guidelines: https://link.springer.com/journal/44320/submission-guidelines#cms

9) For data quantification: please specify the name of the statistical test used to generate error bars and p-values, the number (n) of independent experiments (specify technical or biological replicates) underlying each data point and the test used to calculate p-values in each figure legend. The figure legends should contain a basic description of n, p-values and the test applied. Graphs must include a description of the bars and the error bars (s.d., s.e.m.). Please provide exact p-values (in either the figure or figure legend).

10) Our journal encourages inclusion of *data citations in the reference list* to directly cite datasets that were re-used and obtained from public databases. Data citations in the article text are distinct from normal bibliographical citations and should directly link to the database records from which the data can be accessed. In the main text, data citations are formatted as follows: "Data ref: Smith et al, 2001" or "Data ref: NCBI Sequence Read Archive PRJNA342805, 2017". In the Reference list, data citations must be labeled with "[DATASET]". A data reference must provide the database name, accession number/identifiers and a resolvable link to the landing page from which the data can be accessed at the end of the reference. Further instructions are available at .

11) We replaced Supplementary Information with Expanded View (EV) Figures and Tables that are collapsible/expandable online. EV Figures should be cited as 'Figure EV1, Figure EV2" etc... in the text and their respective legends should be included in the main text after the legends of regular figures.
- For the figures that you do NOT wish to display as Expanded View figures, they should be bundled together with their legends in a single PDF file called *Appendix*, which should start with a short Table of Content. Appendix figures should be referred to in the main text as: "Appendix Figure S1, Appendix Figure S2" etc.
- Additional Tables/Datasets should be labeled and referred to as Table EV1, Dataset EV1, etc. Legends should be provided in a separate tab in case of .xls files. Alternatively, the legend can be supplied as a separate text file (README) and zipped together with the Table/Dataset file.
See detailed instructions here:
https://link.springer.com/journal/44320/submission-guidelines#cms

12) Author contributions: CRediT has replaced the traditional author contributions section because it offers a systematic machine-readable author contributions format that allows for more effective research assessment. Please remove the Authors Contributions from the manuscript and use the free text boxes beneath each contributing author's name in our system to add specific details on the author's contribution. More information is available in our guide to authors.

13) Disclosure statement and competing interests: We updated our journal's competing interests policy in January 2022 and request authors to consider both actual and perceived competing interests. Please review the policy https://link.springer.com/partners/embo-press/editorial-policies#Competing%20interest%20disclosures and update your competing interests if necessary.

14) Every published paper now includes a 'Synopsis' to further enhance discoverability. Synopses are displayed on the journal webpage and are freely accessible to all readers. They include a short stand first (maximum of 300 characters, including space) as well as 2-5 one-sentences bullet points that summarizes the paper. Please write the bullet points to summarize the key NEW findings. They should be designed to be complementary to the abstract - i.e. not repeat the same text. We encourage inclusion of key acronyms and quantitative information (maximum of 30 words / bullet point). Please use the passive voice. Please attach these in a separate file or send them by email, we will incorporate them accordingly.

Please also suggest a striking image or visual abstract to illustrate your article as a PNG file 550 px wide x 300-600 px high.

Please note that these would be the final versions and changes during proofing are usually not allowed.

15) As part of the EMBO Publications transparent editorial process initiative (see our policy here: https://link.springer.com/partners/embo-press/editorial-policies#Peer%20review), Molecular Systems Biology will publish online a Peer Review File (PRF) to accompany accepted manuscripts.
In the event of acceptance, this file will be published in conjunction with your paper and will include the anonymous referee reports, your point-by-point response and all pertinent correspondence relating to the manuscript. Let us know whether you agree with the publication of the PRF and as here, if you want to remove or not any figures from it prior to publication.
Please note that the Author checklist will be published at the end of the PRF.

Molecular Systems Biology has a "scooping protection" policy, whereby similar findings that are published by others during review or revision are not a criterion for rejection. Should you decide to submit a revised version, I do ask that you get in touch after three months if you have not completed it, to update us on the status.

Yours sincerely,

Poonam Bheda, PhD
Scientific Editor
Molecular Systems Biology

Reviewer #1:

The manuscript addresses an important limitation of current genomic foundation models, namely their inability to explain inter-individual expression variation using DNA sequence alone. The proposed lightweight CNN decoding tower (xDecoder), which preserves token-level embeddings and avoids global pooling, is technically sound and shows clear improvements under the reference-pretraining plus individual fine-tuning (rp-) strategy for seen genes.

However, several issues merit further consideration.

First, the overall performance gains of xDecoder require more rigorous evaluation. While the authors report improvements over pretrained models for certain genes (e.g., HLA-DQA1, where rp-Caduceus achieves a Spearman correlation of 0.74 compared to negative correlations from Enformer and AlphaGenome), it remains unclear whether these improvements are statistically robust and generalizable, or whether they reflect gene-specific effects. Given the relatively small training set (50 individuals) and the per-gene evaluation framework, performance estimates may be sensitive to sampling variability. The manuscript would benefit from reporting confidence intervals, permutation-based significance testing, or cross-validation to assess whether the observed improvements are consistently above noise levels. Additionally, summarizing performance distributions across all genes-rather than highlighting selected examples-would help clarify whether the gains are systematic or driven by a subset of highly heritable loci.

Second,the training strategy does not include a validation set and uses the final epoch model for evaluation. Given the relatively small training size (50 individuals and 200 genes), this raises concerns about potential overfitting and the robustness of reported improvements. Incorporating cross-validation or model selection based on held-out validation data would strengthen confidence in the conclusions.

Third, while the authors correctly report the failure of zero-shot prediction on unseen genes, the biological interpretation remains somewhat limited. The manuscript primarily attributes this to limitations in sequence-based modeling, yet alternative explanations rooted in systems biology should be considered. For example, gene expression variation is influenced not only by cis-regulatory variation but also by trans-acting factors, three-dimensional chromatin organization, and nonlinear multi-gene regulatory networks. If a substantial proportion of variance for the 287 unseen genes arises from trans or environmental effects, DNA-only models would be fundamentally constrained.

In this regard, estimating the cis-heritability (h^2_cis) of the unseen genes would provide an important upper bound for achievable R^2. If the cis component explains only a modest fraction of expression variance (e.g., h^2_cis ≈ 0.1), then low predictive performance would reflect biological signal limitations rather than model generalization failure. This analysis would substantially sharpen the claim of a "fundamental limitation."
Fourth, while the integration of ATAC-seq improves zero-shot performance, it changes the problem formulation from purely genotype-based prediction to multi-omic state inference. From a translational perspective, reliance on individual-level ATAC data may limit applicability in settings where only genotype data are available.

Fifth, the definition of the training and testing datasets requires further clarification. The authors state that they focused on 3,259 genes with significant cis-eQTLs in European individuals from the GEUVADIS study. Of these, 200 genes (excluding chromosomes 5 and 10) were randomly selected for individual-level training, 2,972 genes were used for reference-based training, and the remaining 287 genes located on chromosomes 5 and 10 were reserved for testing. It is unclear why chromosomes 5 and 10 were chosen as the held-out set. Was this selection arbitrary, or was there a specific rationale (e.g., chromosome size, gene density, or computational convenience)? A chromosome-based split may reduce sequence similarity between training and testing loci, but it could also introduce biases if those chromosomes differ in cis-heritability, regulatory architecture, or eQTL density. The robustness of the conclusions would be strengthened by demonstrating that results are consistent under alternative gene partitions (e.g., random gene-level splits repeated multiple times, or leave-one-chromosome-out cross-validation). Without such analysis, it is difficult to assess whether the observed zero-shot limitation reflects a generalizable phenomenon or is partially dependent on the specific gene split used in this study.

Finally, the comparison with AlphaGenome may not be entirely balanced, as AlphaGenome is evaluated without fine-tuning due to API constraints, whereas other models undergo individual-level fine-tuning. Clarifying this limitation and tempering direct performance comparisons would improve fairness.

Reviewer #2:

The manuscript introduces xDecoder, a unified decoding framework that leverages embeddings from large-scale Genomic Language Models and Sequence-to-Function models to predict personalized gene expression from paired genome-transcriptome data. The authors provide side-by-side benchmarking of several approaches including using Evo2, Caduceus, and Borzoi as the sequencing embeddings. The paper confirms previous findings that sequence based models perform poorly on predicting personal gene expression. The fine-tuned model xDecoder performs well on seen genes but does not generalize to unseen genes. However, with personal ATAC-seq data the model can generalize at zero-shot settings to unseen genes. While the case studies presented in the paper are interesting, I find the novelty of the paper is still limited. The model needs ATAC to generalize to unseen genes and also suggest that it is still learning from correlative signals.

Major:

A primary concern is whether xDecoder is learning complex regulatory logic or simply acting as a proxy for measured epigenetic states.Chromatin accessibility is both a cause and a consequence of transcription. Simple methods (e.g., summing ATAC signals across a gene body as implemented for example in SnapATAC2 (snapatac2.pp.make_gene_matrix)) often serve as a strong proxy for RNA-seq without requiring deep learning. xDecoder should be compared to such methods to clarify how much the model learns versus simply proxying the RNA with ATAC.

The performance of an ATAC-only version of xDecoder should be presented, to better understand what benefit - if any - there is to incorporating the DNA once we have personalized epigenetic measurements.

ATAC-seq is often more expensive and difficult to collect than RNA-seq. If individual-level multi-omic data is already available, the need for a predictive model is diminished, as the target measurements (expression) are likely already accessible. What would need to be demonstrated is that the DNA + ATAC model reveals some insight about the underlying variants that other methods - such as PrediXcan - would not.

The authors write that: "Despite the limited power in a zero-shot prediction, xDecoder with DNA-only inputs showed promise in few-shot learning (Fig.2A), suggesting potential utility for applications involving rare or somatic variants where only a small number of fine-tuning individuals may be available" However, based on the results presented, few-shot learning from RNA seems to have the same problem as zero-shot learning with ATAC. Why not use PrediXcan instead? The authors mention that genes exist which PrediXcan gets wrong, but to justify a practical utility would require some additional analysis. Can we extract some insights about rare or somatic variants from these genes xDecoder gets right?

Fig 2A shows that DNA-LMs have essentially zero performance after reference training. Moreover, the performance of all three DNA-LM across unseen genes from seen individuals is very poor (Fig S1). However, the DNA-LMs then perform better than S2F models after individual fine-tuning. It is difficult to make sense of these results. Do these DNA-LMs actually add anything to xDecoder besides providing a tokenization and (implicitly) a positional encoding? How does xDecoder perform if trained directly on one-hot sequence or another tokenization scheme (e.g. wordpiece or similar)?

The study relies almost exclusively on Lymphoblastoid Cell Lines (LCLs) from the GEUVADIS dataset. Tissue-Specific Blindness: Most regulatory variation is context-dependent, with distinct enhancers driving expression in different tissues (e.g., heart vs. brain). A model benchmarked solely on immortalized LCLs avoids the core challenge of regulatory genomics: tissue-specific regulation.

Borzoi and AlphaGenome both predict RNA-Seq expression directly; they are likely to perform better than CAGE for expression prediction. AlphaGenome also performs better with 1M basepair as input, you can crop for the target sequence length after getting the prediction.

Minor:

The methods state that individual genomes were constructed by inserting SNPs in the reference genomes. Were individuals with indels in the intervals excluded or were those indels simply ignored? If so, is this not likely to depress performance if an indel is present in the individual but not seen by the model(s)? It would be good to know the frequency of such events in the GEUVADIS data and whether it impacts the presented metrics.

"Genes with higher eQTL density tended to show improved correlation, and xDecoder (rp-Borzoi) achieved higher accuracy than the pretrained Borzoi model across all bins. These observations indicate longer receptive fields increase the probability of incorporating informative regulatory variants". This point is unclear. The methods indicate that Borzoi and rp-Borzoi have identical receptive fields, as the latter takes the embeddings of the former, so how does the performance increase of rp-Borzoi over Borzoi demonstrate anything about the receptive field?

The placement of PrediXcan in figure 2D is confusing. I understand the intent, but since it does not correspond to either the x (where it is in between two categories) or y-axis (the scale goes to ~0.1, whereas the stated value is 0.216), it makes the figure hard to read.

There are four borzoi models. Could the authors please clarify if they used a particular of one of these, or averaged across all four.

Table 1: why is the number for ref-only, DNA+ATAC, task 2 in bold? It does not seem to be the highest in either the column or

the row.
Fig S6: the caption mentions r-Caduceus and rp-Borzoi, but the legend has r-Caduceus and rp-Caduceus
Flashzoi is cited twice, once the published version and once the bioRxiv. Is this intentional?

Reviewer #3:

The paper introduces xDecoder, a framework that uses existing DNA foundation models to predict individual-level gene expression differences. Using paired genome and RNA data from GEUVADIS (lymphoblastoid cell lines from 1000 Genomes individuals), the authors compare six pretrained models under three training setups: using the reference genome only, using individual genomes only, and pretraining followed by fine-tuning on individuals. The main findings are: (1) Fine-tuning enables prediction of expression differences for genes seen during training, with more than a threefold improvement over pretrained models; (2) Predicting expression for completely unseen genes fails when using DNA alone; (3) Adding individual-level ATAC-seq data improves zero-shot performance. The main claim of the paper is that integrating additional molecular data may be necessary for robust personalized prediction, and that the bottleneck may lie less in model architecture than in the information content of DNA alone for cross-individual generalization.

The work has several strengths, including a sensible modeling strategy, a useful benchmarking framework applied consistently across six genomic foundation models, a clear demonstration of few-shot improvements, and careful validation analyses including cross-population evaluation and gene-level stratification. However, I have the following major concerns:

1) The improvement observed when integrating individual-level ATAC-seq is interesting, but it is unclear how much of this gain reflects true DNA×ATAC interaction learning versus simply conditioning on a measured regulatory phenotype that is already strongly correlated with expression. Because chromatin accessibility lies close to transcription in the regulatory cascade, the improvement could reflect access to an intermediate phenotype rather than enhanced genotype-aware modeling. To clarify attribution, the manuscript would benefit from including ATAC-only and reference-DNA+ATAC baselines in the unseen-gene setting. This would help disentangle whether the improvement stems primarily from chromatin accessibility itself or from genotype-aware modeling beyond accessibility.

2) The manuscript clearly demonstrates that DNA-only models fail in the unseen-gene setting, but it does not examine whether this failure is uniform or gene-dependent. It would be valuable to understand whether zero-shot prediction breaks down systematically, or whether certain classes of genes (e.g., high cis-heritability, dense eQTL architecture, promoter-proximal regulation) show partial transfer. A simple stratified analysis by gene-level properties (e.g., eQTL density, regulatory complexity, or heritability proxies) could provide insight into when zero-shot prediction partially succeeds and what biological features limit generalization. This would move the work beyond documenting failure toward identifying mechanistic constraints on sequence-based generalization.

3) The reduced performance when training on European individuals and evaluating on Yoruba individuals suggests possible reliance on population-specific LD structure rather than learning ancestry-robust regulatory principles. This is an important result that should be analyzed more explicitly, particularly given the implications for portability. Even a limited diagnostic analysis-such as relating performance drop to allele frequency shifts, LD decay, or variant novelty rates, or clarifying whether mixed-ancestry training alters the outcome-would significantly strengthen interpretation.

4) Fine-tuning relies on 50 training individuals, but it is unclear whether performance has saturated at this scale. A simple learning curve analysis (e.g., training with 10, 20, 40, and 50 individuals) would help determine whether improvements plateau or remain data-limited. Even if additional data are unavailable, a brief discussion of expected scaling behavior would help contextualize the current results and clarify whether the limitation is architectural or statistical.

Minor points:
- The manuscript relies exclusively on lymphoblastoid cell lines (LCLs). While reasonable given data availability, the discussion should more explicitly acknowledge that conclusions may be cell-type specific and outline expectations for other tissues.
- The ATAC experiment uses 94 overlapping samples. The sample size should be clearly stated in the main text, and uncertainty estimates (e.g., confidence intervals) would help contextualize robustness.
- The paper "Flashzoi: an enhanced borzoi for accelerated genomic analysis" appears to be cited twice; the redundant citation should be removed.

Fig. 1A contains a typo ("Caudcues" instead of "Caduceus").

Responses to
Comments of reviewers

xDecoder unlocks the potential of genomic foundation models for few-shot personal gene expression prediction

Shumin Li, Ruibang Luo, Yuanhua Huang

Manuscript Number: MSB-2025-13544

Dear Dr. Huang,

Thank you again for submitting your work to Molecular Systems Biology. We have now heard back from the three reviewers who agreed to evaluate your study. As you will see below, the reviewers appreciate that the proposed approach addresses a timely topic. However, they raise a series of concerns, which we would ask you to address in a major revision.

Without repeating all the comments listed below, some of the more fundamental issues raised are the following:

- ATAC improvements to the model are not sufficiently investigated. Additional evaluation on whether there is indeed an interaction of this information with DNA sequence alone or rather that ATAC data are inherently correlated with gene expression should be performed.

- Unclear whether the improvements showcased are for specific genes or more generalizable. This should be further investigated and/or presented more clearly.

All other issues raised would need to be satisfactorily addressed. Please let me know in case you would like to discuss in further detail any of the comments, I would be happy to schedule a call.

Yours sincerely,

Poonam Bheda, PhD
Scientific Editor
Molecular Systems Biology

Dear Dr. Bheda,

We thank you and the reviewers for the constructive and detailed assessment of our manuscript. In this revision, we focused especially on the two fundamental issues highlighted in the editorial decision: first, whether the improvement from ATAC-seq reflects meaningful genotype-aware modeling or simply access to a regulatory phenotype correlated with expression; and second, whether the reported xDecoder improvements are systematic or driven by specific genes. We have substantially expanded the analyses, added new controls, and revised the interpretation accordingly.

First, to clarify the role of ATAC-seq, we added ATAC-only control analyses in both seen-gene and unseen-gene settings. These analyses led us to refine the interpretation of the ATAC experiment. In the seen-gene setting, where RNA labels for the target genes are available during training, rp-Caduceus with DNA+ATAC substantially outperformed a supervised ATAC-only linear baseline constructed from the same 64 kb TSS-centered window. Among ATAC-predictable seen genes, rp-Caduceus DNA+ATAC achieved a mean Spearman correlation of 0.294, compared with 0.197 for the ATAC-only baseline. This indicates that measured accessibility alone is not sufficient, and that sequence-derived information and paired genome–transcriptome training remain important for gene-specific individual-level prediction. In addition, for genes where the ATAC-only baseline was uninformative, DNA-only xDecoder performed better than DNA+ATAC, further showing that the contribution of ATAC is gene-dependent and that ATAC is not a substitute for sequence modeling.

In the unseen-gene setting, the result is different and biologically informative. A summed ATAC-only baseline was competitive with rp-Caduceus DNA+ATAC, indicating that measured chromatin accessibility is a strong regulatory-state proxy for expression in this difficult zero-shot setting. We therefore revised the manuscript to interpret this experiment as identifying a key bottleneck of current DNA-only models: they do not yet reliably infer individual-specific chromatin states from DNA sequence alone. This interpretation is further supported by the modest concordance between AlphaGenome-predicted and observed personal ATAC signals. Thus, the key implication of the ATAC experiment is not that ATAC-seq should be required as a prediction-time replacement for RNA-seq, but that individual-level multi-omic data can provide important auxiliary supervision or intermediate regulatory-state targets for training more effective DNA-based personalized regulatory models. These revisions are described in detail in our responses to Reviewer 2 Major Comments #1–2, Reviewer 1 Comment #4, and Reviewer 3 Comment #1.

Second, to determine whether xDecoder improvements are systematic or gene-specific, we added distribution-level performance summaries, paired comparisons between reference-trained and individually fine-tuned models, permutation-based significance testing, etc. These analyses show that individual-level DNA–RNA fine-tuning improves prediction for seen genes across the gene distribution, rather than only for selected examples. For example, rp-Caduceus increased the number of genes with significant cross-individual correlation in permutation testing and achieved more than threefold improvement over pretrained baselines in the few-shot seen-gene setting. We also added several analyses to clarify when generalization succeeds or fails. These include stratification by PrediXcan-based cis-predictability, learning-curve analyses with increasing training sample size, cross-population portability diagnostics, etc. Together, these results support a more nuanced conclusion:

xDecoder enables few-shot personalized expression prediction for genes observed during training, but DNA-only zero-shot transfer to unseen genomic contexts remains limited, even with larger training sets and even among genes with stronger cis-predictable signal. These analyses are described in our responses to Reviewer 1 Comments #1 and #3, and Reviewer 3 Comments #2–4.

Overall, we believe the whole manuscript has been substantially improved. It shows that paired genome–transcriptome fine-tuning can unlock few-shot personalized expression prediction from genomic foundation-model embeddings, while the ATAC and zero-shot analyses identify a key remaining bottleneck: current DNA-only sequence models still fail to infer individual-specific regulatory states well enough to generalize across loci. This motivates future personalized regulatory models that combine pretrained sequence encoders with variant-aware learning and individual-level multi-omic supervision.

Point-by-point responses to the comments of Reviewer #1:

The manuscript addresses an important limitation of current genomic foundation models, namely their inability to explain inter-individual expression variation using DNA sequence alone. The proposed lightweight CNN decoding tower (xDecoder), which preserves token-level embeddings and avoids global pooling, is technically sound and shows clear improvements under the reference-pretraining plus individual fine-tuning (rp-) strategy for seen genes.

However, several issues merit further consideration.

Response: We thank the reviewer for the positive feedback and constructive comments.

#1 First, the overall performance gains of xDecoder require more rigorous evaluation. While the authors report improvements over pretrained models for certain genes (e.g., HLA-DQA1, where rp-Caduceus achieves a Spearman correlation of 0.74 compared to negative correlations from Enformer and AlphaGenome), it remains unclear whether these improvements are statistically robust and generalizable, or whether they reflect gene-specific effects. Given the relatively small training set (50 individuals) and the per-gene evaluation framework, performance estimates may be sensitive to sampling variability. The manuscript would benefit from reporting confidence intervals, permutation-based significance testing, or cross-validation to assess whether the observed improvements are consistently above noise levels. Additionally, summarizing performance distributions across all genes-rather than highlighting selected examples-would help clarify whether the gains are systematic or driven by a subset of highly heritable loci.

Response: We thank the reviewer for pointing this out. In response, we added three analyses. First, we now present the distribution of per-gene Spearman correlations across all genes for both pretrained models and xDecoder-based models. It shows that the individually fine-tuned xDecoder models are shifted toward higher correlations relative to the pretrained baselines.

Second, we performed paired comparisons between reference-trained (r-) and reference-pretrained plus individual-fine-tuned (rp-) models using the same DNA sequence embeddings. These analyses show that individual-level fine-tuning improves across-unseen-individual performance for a substantial proportion of genes, with especially clear gains among genes

with higher cis-predictability; notably, many genes with negative or near-zero correlations in the reference-trained models become positively correlated after fine-tuning.

Third, as suggested, we added per-gene permutation-based significance testing. The number of genes with significant cross-individual correlation is consistently higher in the fine-tuned models than in the corresponding baselines.

We have updated the main text as:

(P3, line 15-line 20): "In this few-shot seen-gene setting, *xDecoder consistently improved cross-individual correlation, achieving >3 folds gains over pretrained Enformer and Borzoi, as well as over reference-trained xDecoder variants (from 0.089 to 0.194) (Fig. 2A,* ***Fig. S3, S4****).* ***Fine-tuning at the individual level also corrected the sign of correlation for a subset of genes (Fig. S5) and increased the number of genes showing significant cross-individual correlation in permutation tests (e.g., 99 for rp- Caduceus versus 64 for AlphaGenome; Fig. S6)"***

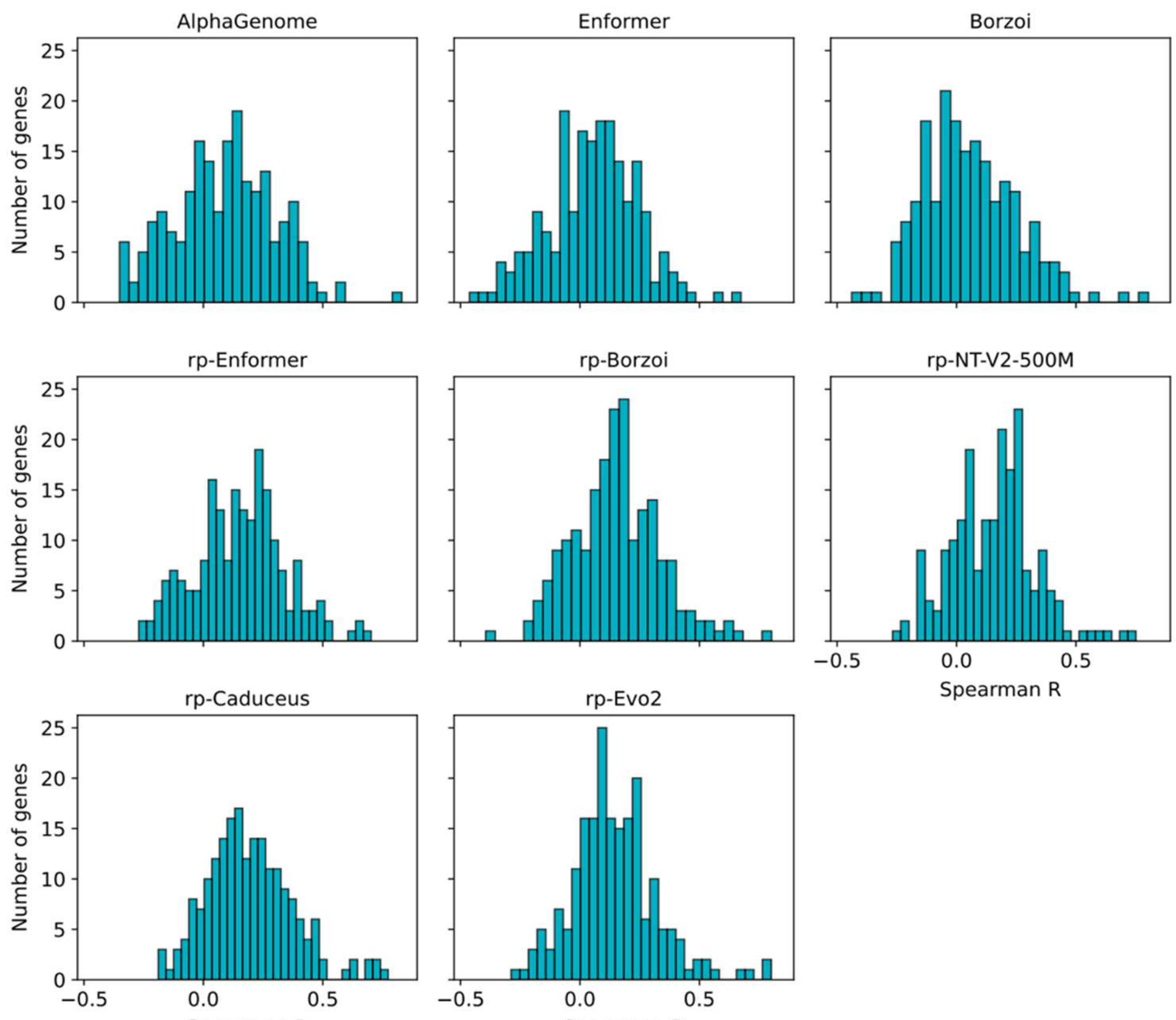

Figure 1 (added as Fig. S4). Distribution of Spearman R for across unseen individuals per seen gene.

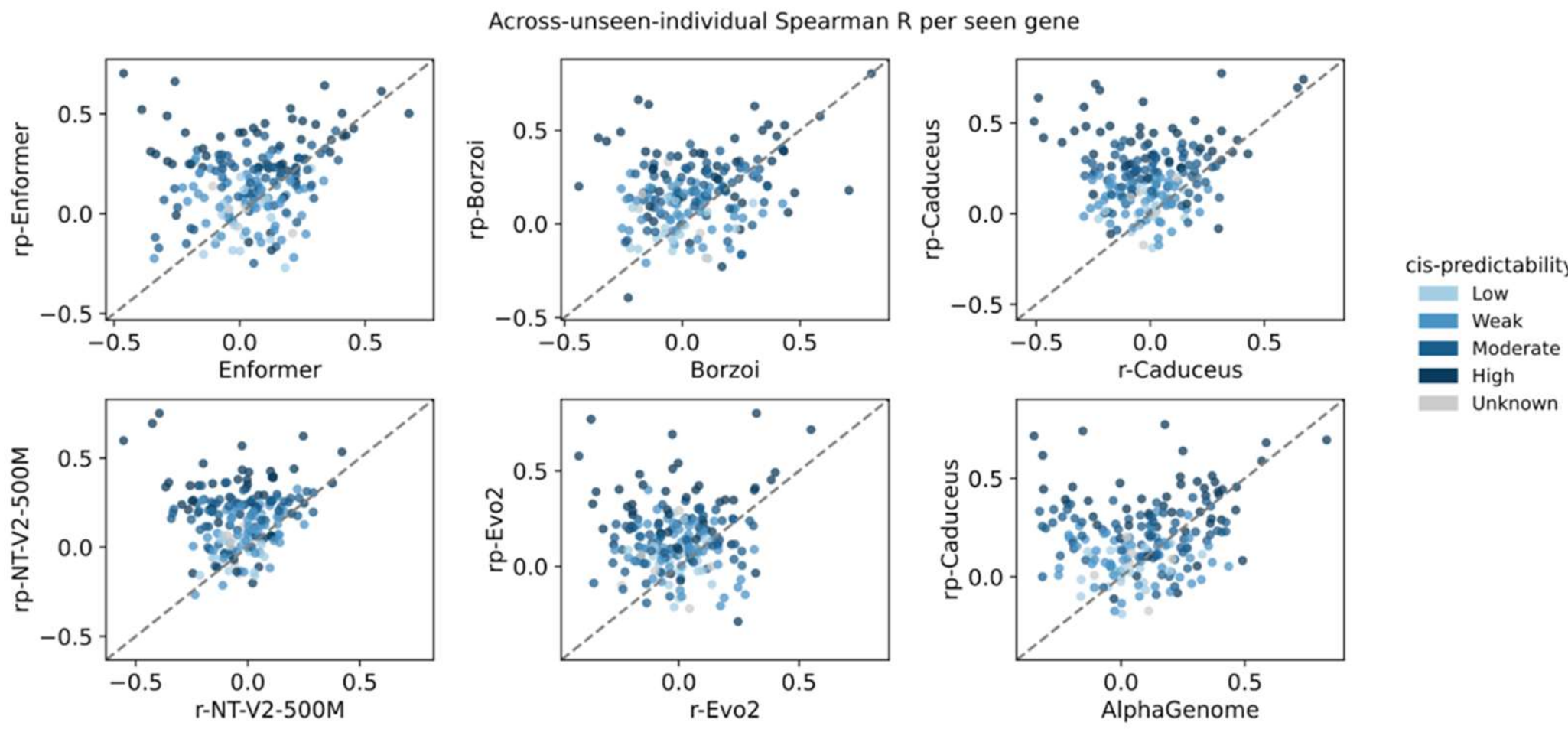

Figure 2 (added as Fig. S5). Paired across-unseen-individual Spearman R comparison of reference-trained and individually fine-tuned models across 200 seen genes. Each point represents one gene. Gene-wise cis-predictability was defined using PrediXcan performance trained from 362 individuals: low (R < 0.10), weak (0.10 ≤ R < 0.25), moderate (0.25 ≤ R < 0.40), and high (R ≥ 0.40). "Unknown" indicates genes that could not be predicted by PrediXcan.

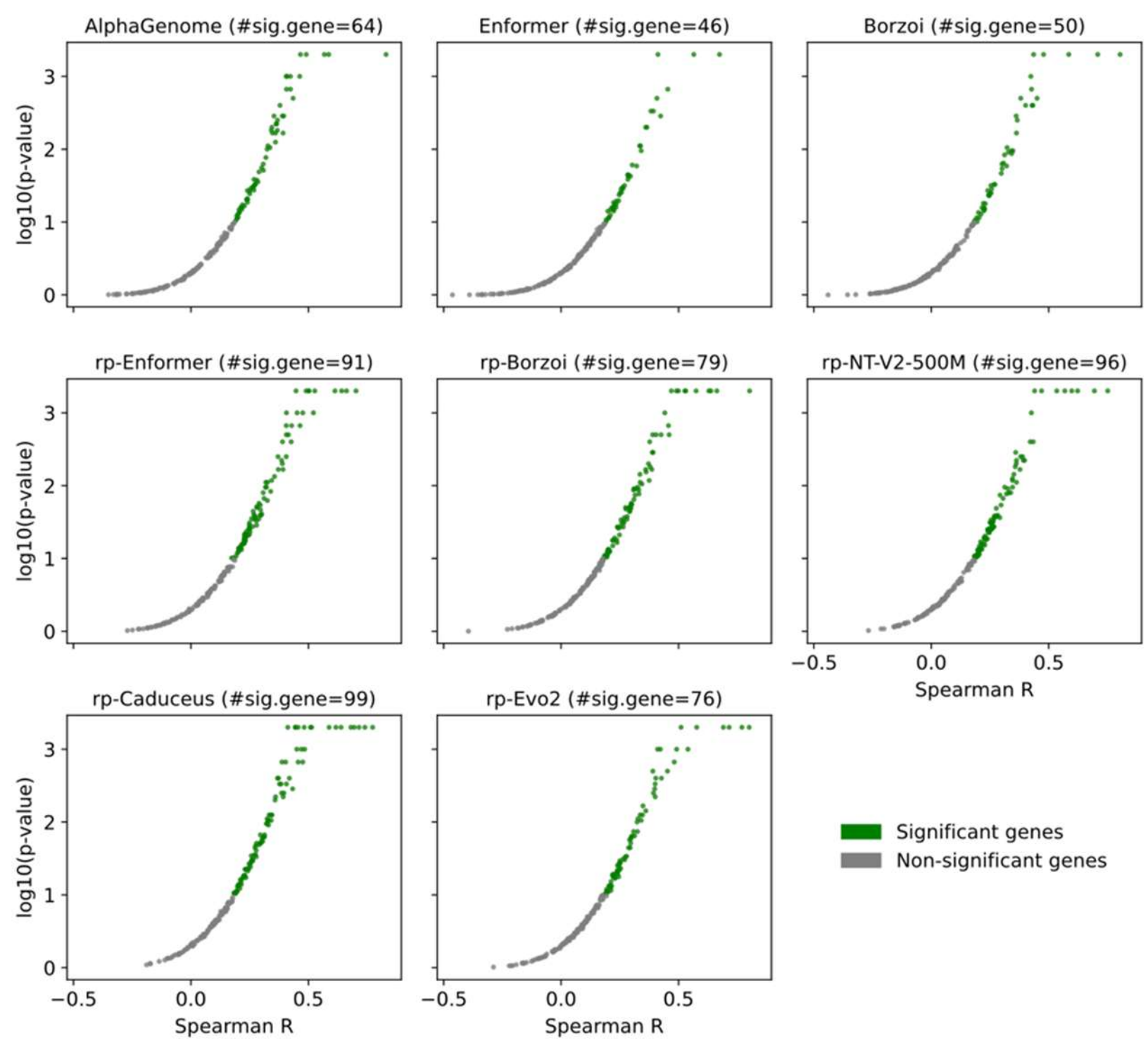

Figure 3 (added as Fig. S6). Permutation-based significance analysis of cross-unseen-individual correlation per seen gene. For each gene, the significance of the cross-unseen-individual Spearman correlation between predicted and observed expression was evaluated using a permutation test. The observed log-TPM values were permuted across testing individuals to generate a gene-specific null distribution (n=2000). A one-sided empirical *p*-value was computed from this null distribution, and genes with one-sided p < 0.1 were defined as significant.

#2 Second,the training strategy does not include a validation set and uses the final epoch model for evaluation. Given the relatively small training size (50 individuals and 200 genes), this raises concerns about potential overfitting and the robustness of reported improvements. Incorporating cross-validation or model selection based on held-out validation data would strengthen confidence in the conclusions.

Response: We thank the reviewer for this helpful suggestion. In the original submission, all evaluations were performed on a strictly chromosome-based held-out test set that was never used during training, so the reported results already reflect generalization to unseen data. We agree, however, that using a dedicated validation set or cross-validation would provide a more rigorous strategy for checkpoint selection, especially given the limited number of paired training samples.

In the main benchmark, we used the final checkpoint consistently across models to maintain a controlled comparison across many genes and backbone representations. Validation-based model selection at scale would have substantially increased the computational cost. To partially address this concern in a computationally tractable way, we performed an additional validation-based early-stopping experiment for the xDecoder rp-Caduceus model using an independent validation split of 50 individuals for HLA-DQA1 (tolerance = 8 epochs). Because checkpoint selection was based on a single validation gene, this experiment should be interpreted as a limited sanity check rather than a globally optimized selection strategy. In results, this validation-based checkpoint gave slightly lower but overall similar performance on the held-out test set relative to the final-epoch checkpoint (Task 1: 0.157 vs 0.194; Task 2: -0.005 vs 0; Task 3: 0.147 vs 0.176), indicating that checkpoint selection modestly affects absolute performance but does not materially alter the overall qualitative conclusions.

We updated the manuscript as:

(P9, lines 42-45): ***<u>”As an additional sanity check, we performed a validation-based early-stopping experiment for rp-Caduceus using an independent validation split of 50 individuals for HLA-DQA1 (tolerance = 8 epochs), which yielded slightly lower but overall similar performance to the final-epoch checkpoint (Fig. S24)”</u>***

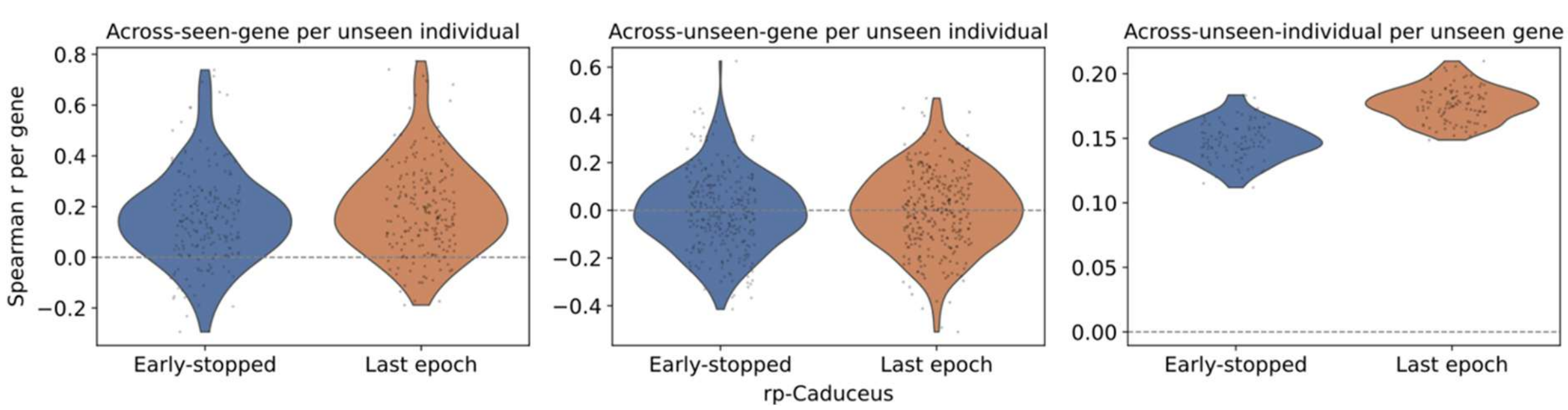

Figure 4 (added as Fig. S24). Effect of validation-based early stopping on xDecoder rp-Caduceus performance. Held-out test performance of xDecoder rp-Caduceus under two checkpoint-selection strategies: final-epoch checkpoint (40 epochs) and validation-based early stopping (tolerance = 8 epochs). Early stopping was determined using an independent validation split of 50 individuals for HLA-DQA1.

#3 Third, while the authors correctly report the failure of zero-shot prediction on unseen genes, the biological interpretation remains somewhat limited. The manuscript primarily attributes this to limitations in sequence-based modeling, yet alternative explanations rooted in systems biology should be considered. For example, gene expression variation is influenced not only by cis-regulatory variation but also by trans-acting factors, three-dimensional chromatin organization, and nonlinear multi-gene regulatory networks. If a substantial proportion of variance for the 287 unseen genes arises from trans or environmental effects, DNA-only models would be fundamentally constrained. In this regard, estimating the cis-heritability (h^2_cis) of the unseen genes would provide an important upper bound for achievable R^2. If the cis component explains only a modest fraction of expression variance (e.g., $h^2\_cis \approx 0.1$), then low predictive performance would reflect biological signal limitations rather than model generalization failure. This analysis would substantially sharpen the claim of a "fundamental limitation."

Response: We thank the reviewer for this constructive suggestion. To compare the zero-shot results with an estimate of cis-explainable signal, we computed a PrediXcan-style gene-specific linear predictor for each of the 287 unseen genes using all available training individuals (n = 362). We use the PrediXcan Spearman correlation as a cis-predictability indicator. We then stratified the 287 genes into four groups based on PrediXcan cis-predictability: low ($R < 0.10$), weak ($0.10 \le R < 0.25$), moderate ($0.25 \le R < 0.40$), and high ($R \ge 0.40$) (Fig. 5A). Across models, zero-shot performance increases with cis-predictability—most notably for AlphaGenome—yet remains substantially below the gene-specific supervised reference, even for moderate and high cis-predictability genes (Fig. 5BC), highlighting the difficulty of transferring cis-regulatory rules across loci. Conversely, genes with low/weak cis-predictability might have a larger contribution from trans-regulatory and context effects.

We have added this analysis and corresponding discussion to the revised manuscript：

(P5, lines 32-36): ***<u>“We further stratified the unseen genes by PrediXcan cis-predictability (Methods;Fig. S16A). It shows zero-shot performance tended to increase for genes with stronger cis signal—mostnotably for AlphaGenome—yet remained substantially below the gene-specific supervised PrediXcan reference even among moderately/highly cis-predictable genes (Fig. S16B, C), highlighting the difficulty of transferring cis-regulatory rules across loci. ”</u>***

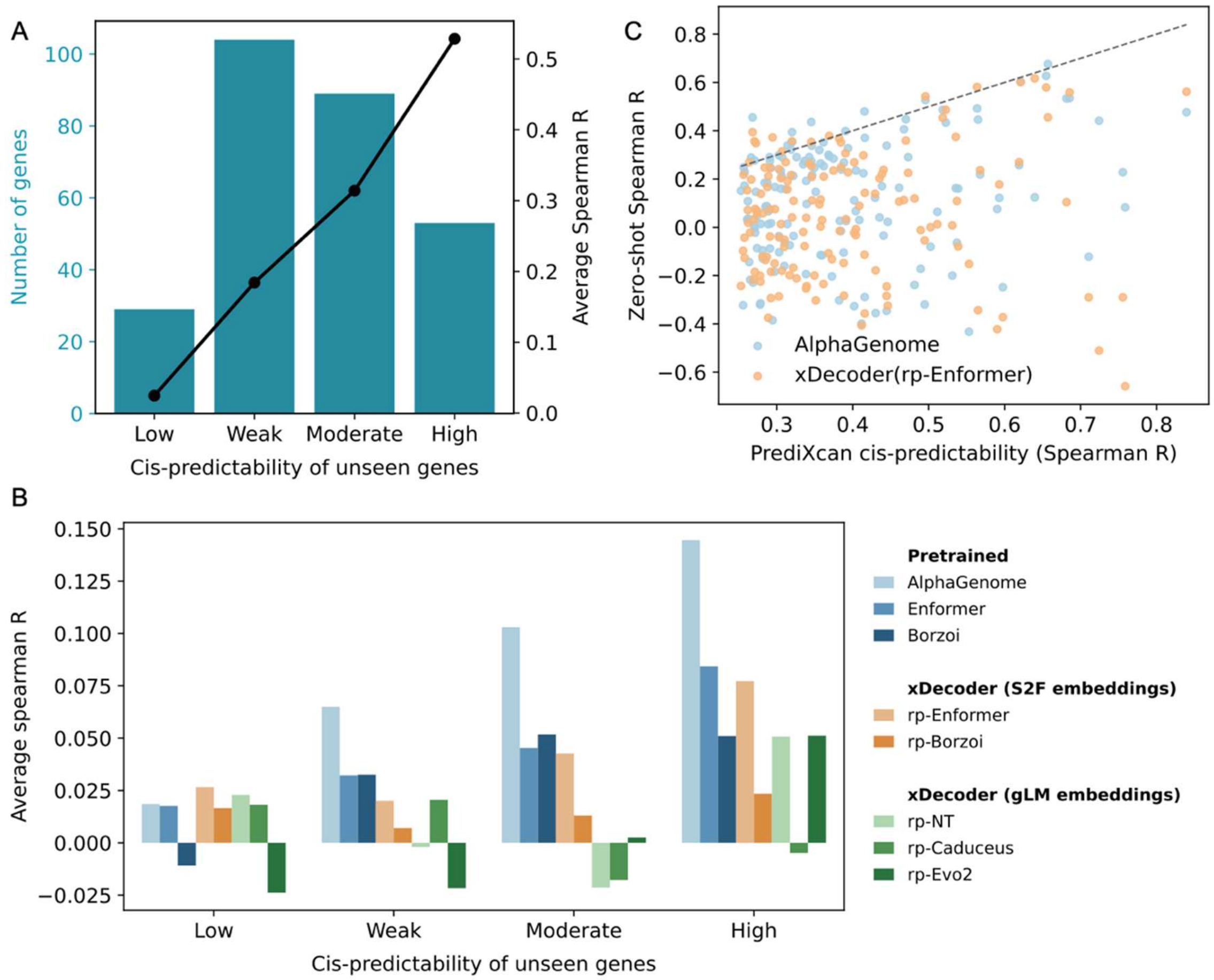

Figure 5 (Added as Fig.S16). Cis-predictability stratifies zero-shot performance on unseen genes. A, Distribution of unseen genes across PrediXcan cis-predictability groups and the mean PrediXcan performance within each group. PrediXcan cis-predictability was estimated using a gene-specific elastic net model trained on common cis variants (MAF ≥ 0.05) 131 kb centered on the TSS, using all available training individuals (n = 362) and evaluated 100 testing individuals. Groups are defined as low (R < 0.10), weak (0.10 ≤ R < 0.25), moderate (0.25 ≤ R < 0.40), and high (R ≥ 0.40). B, Mean zero-shot cross-individual Spearman correlation for each model within each cis-predictability group. C, Gene-level relationship between PrediXcan cis-predictability and model zero-shot performance (Spearman R) for unseen genes with moderate/high cis-predictability.

#4 Fourth, while the integration of ATAC-seq improves zero-shot performance, it changes the problem formulation from purely genotype-based prediction to multi-omic state inference. From a translational perspective, reliance on individual-level ATAC data may limit applicability in settings where only genotype data are available.

Response: We thank the reviewer for this thoughtful comment. We agree that incorporating individual-level ATAC-seq changes the problem formulation from purely genotype-based prediction to a form of multi-omic state inference, and therefore may reduce applicability in settings where only genotype data are available. That said, we believe this analysis remains informative because it helps clarify what current sequence-only models may be missing for personalized expression prediction, particularly in the zero-shot setting. In our study, the

improvement obtained with observed ATAC suggests that individual-specific regulatory-state information provides complementary signals beyond DNA sequence alone.

To further investigate this point, we added an analysis on unseen genes comparing gene-wise, across-individual concordance between AlphaGenome-predicted and observed gene-body ATAC signals, where ATAC signal was aggregated by summing signal within each gene body. This analysis showed only modest agreement overall between predicted and observed personal ATAC signals (mean Spearman r=0.168). We further compared, for the same genes, the concordance of AlphaGenome predictions for gene-body ATAC and gene expression, and found that genes with higher ATAC concordance also tended to show higher expression concordance. These results suggest that limited recovery of individual-specific chromatin variation may be one factor contributing to the weak performance of sequence-only models for personalized gene expression prediction, especially for unseen genes.

We therefore present the ATAC-integrated analysis not as a replacement for genotype-only prediction, but rather as an informative diagnostic experiment that highlights the importance of regulatory-state information and the current gap between sequence-based prediction and true individual-specific chromatin state. We have revised the manuscript as:

(P7, lines 29-33): <u>"At the same time, the poor performance of DNA-only models in this setting suggests that current sequence models do not yet reliably infer individual-specific chromatin states from DNA sequence alone. Consistently, AlphaGenome-predicted gene-level ATAC signals showed only modest concordance with observed ATAC signals across individuals (mean Spearman R = 0.168; Fig. S23A)"</u>

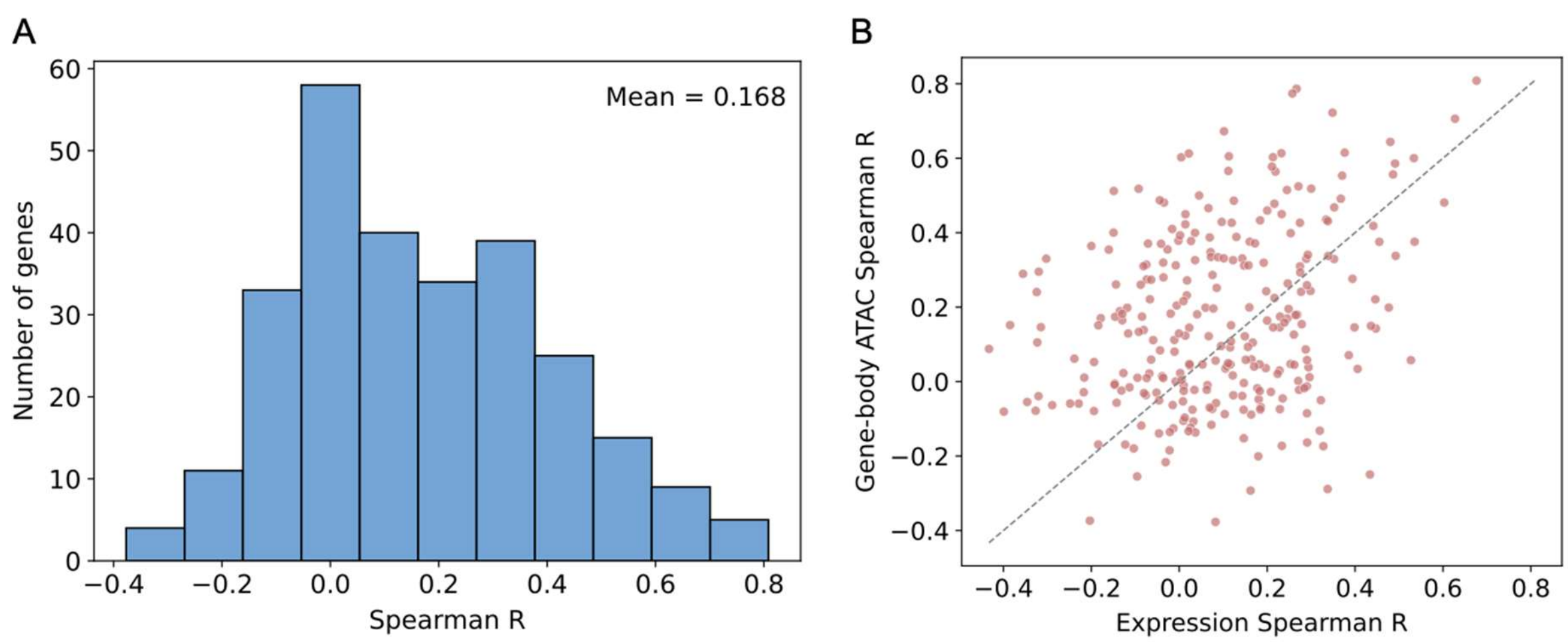

Figure 6 (added as Fig. S23). Comparison of AlphaGenome performance on gene expression and gene-body ATAC for unseen genes. AlphaGenome was evaluated on held-out unseen genes in a zero-shot setting (Test set 3 in Main Fig. B). For each gene, predicted and observed gene-body ATAC signals were obtained by summing ATAC signal within the gene body for each individual, through the Spearman correlation across individuals. Gene-wise Spearman correlations across individuals were also obtained for predicted versus observed gene expression. (A) Distribution of gene-wise gene-body ATAC correlations. (B) Scatter plot comparing gene-wise expression correlation and gene-wise gene-body ATAC correlation; each dot represents one gene.

#5 Fifth, the definition of the training and testing datasets requires further clarification. The authors state that they focused on 3,259 genes with significant cis-eQTLs in European individuals from the GEUVADIS study. Of these, 200 genes (excluding chromosomes 5 and 10) were randomly selected for individual-level training, 2,972 genes were used for reference-based training, and the remaining 287 genes located on chromosomes 5 and 10 were reserved for testing. It is unclear why chromosomes 5 and 10 were chosen as the held-out set. Was this selection arbitrary, or was there a specific rationale (e.g., chromosome size, gene density, or computational convenience)? A chromosome-based split may reduce sequence similarity between training and testing loci, but it could also introduce biases if those chromosomes differ in cis-heritability, regulatory architecture, or eQTL density. The robustness of the conclusions would be strengthened by demonstrating that results are consistent under alternative gene partitions (e.g., random gene-level splits repeated multiple times, or leave-one-chromosome-out cross-validation). Without such analysis, it is difficult to assess whether the observed zero-shot limitation reflects a generalizable phenomenon or is partially dependent on the specific gene split used in this study.

Response: We thank the reviewer for raising this point. Our use of a chromosome-based split was intended to provide a stricter test of cross-locus generalization by separating training and testing genes in genomic position, thereby reducing possible local sequence leakage. Chromosomes 5 and 10 were used as a fixed held-out test set.

To assess if such a partition would introduce chromosome-specific bias or not , we compared the held-out chromosomes versus the remaining chromosomes in two ways. First, we examined the distribution of gene-level cis-predictability categories derived from PrediXcan-based estimates (low, weak, moderate, high, also see response for comment 3). The held-out and non-held-out chromosomes showed highly similar distributions, with no obvious enrichment of unusually easy or unusually difficult genes in the held-out set. Second, we compared the number of significant cis-eQTL SNPs per gene and again observed a comparable distribution between held-out and remaining chromosomes. These analyses suggest that the chromosomes 5 and 10 test set is not obviously atypical with respect to cis-regulatory predictability or eQTL burden.

We agree that additional partitioning strategies, such as repeated random gene-level splits or leave-one-chromosome-out evaluation, would further strengthen the generalizability of the conclusions. However, these analyses would require substantially greater computational resources, and our current analyses did not indicate obvious differences between the held-out chromosomes and the remaining chromosomes. We have therefore clarified this point in the revised manuscript as:

(P2, lines 30-32): <u>"This chromosome-based split was designed to provide a stringent locus-disjoint evaluation, and the held-out genes showed comparable cis-predictability to genes on the remaining chromosomes (Fig. S1)"</u>

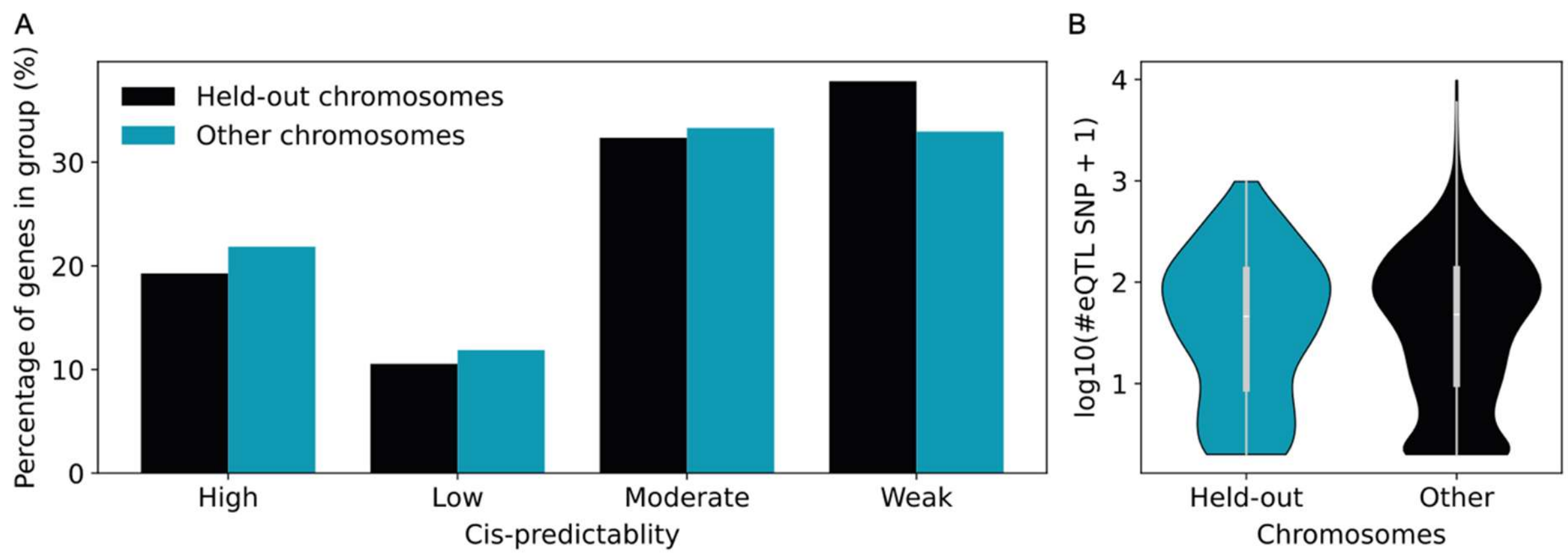

Figure 7 (added as Fig. S1). Held-out chromosomes show similar cis-regulatory characteristics to the remaining chromosomes. A, Percentage of genes in each cis-predictability category (low, weak, moderate, and high) for genes on the held-out chromosomes (chromosomes 5 and 10) and the remaining chromosomes. Cis-predictability was estimated using a PrediXcan-based linear model. B, Distribution of the number of significant cis-eQTL SNPs per gene, shown as log10(#eQTL SNP + 1), for genes on the held-out chromosomes and the remaining chromosomes.

#6 Finally, the comparison with AlphaGenome may not be entirely balanced, as AlphaGenome is evaluated without fine-tuning due to API constraints, whereas other models undergo individual-level fine-tuning. Clarifying this limitation and tempering direct performance comparisons would improve fairness.

Response: We thank the reviewer for pointing this out. AlphaGenome was evaluated without fine-tuning and is presented as an inference-only pretrained baseline as its pretrained weights were not publicly available at the time of our assessment. In contrast, Enformer and Borzoi are used both as pretrained baselines and as embedding providers for xDecoder, which is then fine-tuned with paired individual-level data. We have revised the manuscript to clarify it as:

(P3, lines 17-20): ***"****Fine-tuning at the individual level also corrected the sign of correlation for a subset of genes (Fig. S5) and increased the number of genes showing significant cross-individual correlation in permutation tests (e.g., 99 for rp-Caduceus versus 64* ***for reference-pretrained AlphaGenome****; Fig. S6)****"***

Point-by-point responses to the comments of Reviewer #2:

The manuscript introduces xDecoder, a unified decoding framework that leverages embeddings from large-scale Genomic Language Models and Sequence-to-Function models to predict personalized gene expression from paired genome-transcriptome data. The authors provide side-by-side benchmarking of several approaches including using Evo2, Caduceus, and Borzoi as the sequencing embeddings. The paper confirms previous findings that sequence based models perform poorly on predicting personal gene expression. The fine-tuned model xDecoder performs well on seen genes but does not generalize to unseen genes. However, with personal ATAC-seq data the model can generalize at zero-shot settings to unseen genes. While the case studies presented in the paper are interesting, I find the

novelty of the paper is still limited. The model needs ATAC to generalize to unseen genes and also suggest that it is still learning from correlative signals.

Response: We thank the reviewer for the careful evaluation of our work and for highlighting the central interpretability and utility questions.

Major:

#1 A primary concern is whether xDecoder is learning complex regulatory logic or simply acting as a proxy for measured epigenetic states.Chromatin accessibility is both a cause and a consequence of transcription. Simple methods (e.g., summing ATAC signals across a gene body as implemented for example in SnapATAC2 (snapatac2.pp.make_gene_matrix)) often serve as a strong proxy for RNA-seq without requiring deep learning. xDecoder should be compared to such methods to clarify how much the model learns versus simply proxying the RNA with ATAC.
The performance of an ATAC-only version of xDecoder should be presented, to better understand what benefit - if any - there is to incorporating the DNA once we have personalized epigenetic measurements.

Response: We thank the reviewer for this important comment. We agree that chromatin accessibility can serve as a strong proxy for gene expression, and that improvement after adding ATAC should not by itself be interpreted as evidence that the model has learned complex sequence-mediated regulatory logic. To address this point, we added ATAC-only control analyses and revised the manuscript to clarify the interpretation of the DNA+ATAC experiment.

First, in the seen-gene setting, we evaluated whether simple ATAC-based models can predict individual-level expression. We constructed ATAC-only linear models using ATAC peaks within the same 64 kb TSS-centered window used by rp-Caduceus (DNA+ATAC). For the ATAC linear model, we tested to merge the peaks into 1 or 5 meta peaks, using sum-up or average strategy within the meta peak (Table 1). ElasticNet models were trained separately for each gene using the same 50 training and 44 testing individuals as in the rp-Caduceus (DNA+ATAC) experiments. The results are comparable to each other, and we select 5 meta peaks with sum-up to compare with xDecoder as it yields most valid predictions. Among these 125 ATAC-predictable genes, rp-Caduceus (DNA+ATAC) achieved a mean Spearman correlation of 0.294, compared with 0.197 for the ATAC-only linear baseline (Fig. 8A). Conversely, among genes for which the ATAC-only linear model produced uninformative predictions, xDecoder with DNA-only input achieved a mean Spearman correlation of 0.212, compared with 0.186 for DNA+ATAC, indicating that the contribution of ATAC is gene-dependent and that some seen-gene predictions are better captured by sequence-derived signal than by the simple ATAC proxy (Fig. 8B).

Second, in the zero-shot unseen-gene setting, we implemented a simple ATAC-only baseline by summing ATAC signal across the same 64 kb genomic context around TSS and correlating this score with observed expression across held-out individuals. This baseline yielded valid predictions for 275 of 287 genes. Among these genes, the summed ATAC-only baseline achieved a mean Spearman correlation of 0.147, compared with 0.133 for rp-Caduceus (DNA+ATAC) on the same set (Fig. 8C). This result supports the point that ATAC can act as a strong proxy for expression, and indicates that the aggregate improvement of DNA+ATAC

over DNA-only in the unseen-gene setting is largely attributable to measured ATAC signal rather than to personalized DNA sequence alone.

We ask whether DNA provides any additional information once ATAC is available, we stratified genes by PrediXcan cis-predictability and compared rp-Caduceus (DNA+ATAC) with the ATAC-only baseline. We computed ΔR by the difference between rp-Caduceus (DNA+ATAC) and ATAC-only, and classified genes as improved, unchanged, or worsened using a threshold of ΔR>0.02, -0.02<=ΔR<=0.02 , ΔR<-0.02. This analysis showed that rp-Caduceus (DNA+ATAC) provided greater relative benefit for genes with stronger cis-predictability, whereas the ATAC-only baseline was more competitive for genes with weak cis signal (Fig. 8D). This suggests that personalized DNA contributes most when expression variation has a detectable cis-genetic component, while ATAC alone can be a strong expression proxy for genes with weak cis-genetic signals.

Based on these analyses, we revised the manuscript to make the interpretation of the DNA+ATAC experiment more cautious. To clarify, we do not present DNA+ATAC prediction as a practical replacement for RNA-seq, nor as definitive evidence that the model learns regulatory logic beyond measured chromatin accessibility. Instead, we interpret the ATAC experiment **as diagnostic**: the large improvement from adding measured ATAC indicates that DNA-only models currently fail to infer individual-specific regulatory states from sequence alone. This motivates future work in which multi-omic measurements such as ATAC-seq may be used as auxiliary supervision or intermediate labels to improve DNA-only models, rather than necessarily as required inputs at prediction time. We have revised the manuscript as:

(P7, lines 3-38): "To distinguish whether the improvement from adding ATAC reflects model learning beyond a simple chromatin-accessibility proxy, we added ATAC-only analyses tailored to the two evaluation settings. In the seen-gene setting, RNA labels for the target genes are available during training, so we constructed a supervised ATAC-only baseline by training per-gene ElasticNet models using ATAC features summarized within the same 64 kb TSS-centered window used by rp-Caduceus. Specifically, ATAC peaks within the context window were aggregated into five regional features and used to predict expression across individuals under the same training and testing split. This baseline asks whether a simple gene-specific mapping from measured accessibility to expression is sufficient. In this setting, rp-Caduceus (DNA+ATAC) outperformed the ATAC-only ElasticNet baseline (0.294 vs 0.197 for 125 ATAC predictable genes, Fig.S22A), indicating that the few-shot model is not merely reproducing a supervised accessibility–expression proxy. Rather, paired genome–transcriptome training appears to learn gene-specific individual-level variation beyond what is captured by this ATAC-only linear model. Specifically, for 75 ATAC-non predictable genes, rp-Caduceus (DNA-only) outperforms rp-Caduceus (DNA+ATAC), indicating some seen-gene predictions are better captured by sequence-derived signal than by the simple ATAC proxy (Fig. S22B).

In the unseen-gene setting, a supervised per-gene ATAC model is not applicable because RNA labels from the held-out genes are not available during training. We therefore used a simple unsupervised ATAC-only gene-activity baseline by summing ATAC signal across the same 64 kb genomic context and correlating this score with observed expression across held-out individuals. This summed-ATAC baseline was

competitive with rp-Caduceus (DNA+ATAC), supporting that measured ATAC can serve as a strong proxy for expression (Fig. S22C, Table 1). Stratification by cis-predictability further showed that the relative benefit of rp-Caduceus (DNA+ATAC) over ATAC-only was more evident for genes with stronger cis-genetic signal, whereas ATAC alone was more competitive for genes with weak cis-genetic signal (Fig. S22D). Nevertheless, both ATAC-only and DNA+ATAC models remained below the per-gene trained PrediXcan reference (Fig. S19B). Together, these results suggest that individual-level chromatin accessibility is critical for predicting personal gene expression at unseen genomic contexts. The competitive performance of the ATAC-only baseline indicates that measured ATAC provides regulatory-state information that is highly informative for expression variation. At the same time, the poor performance of DNA-only models in this setting suggests that current sequence models do not yet reliably infer individual-specific chromatin states from DNA sequence alone. Consistently, AlphaGenome-predicted gene-level ATAC signals showed only modest concordance with observed ATAC signals across individuals (mean Spearman R = 0.168; Fig. S23A), which may partly explain the limited zero-shot generalization of DNA-only models across loci (Fig.S23B). Therefore, rather than treating DNA+ATAC as a practical replacement for RNA-seq, we interpret this experiment as revealing a key bottleneck of current DNA-only models. A promising future direction is to use individual-level multi-omic data, including ATAC-seq, as auxiliary supervision or intermediate regulatory-state targets during training, with the goal of improving DNA-based prediction at unseen loci.”

Table 1. Performance of ATAC-only elasticNet baseline on 200 random genes. The ATAC peaks were aggregated by binning into meta peaks through summing-up or averaging.

#Meta peaks	Aggregation	#Predictable genes	Mean Spearman R
1	sum	112	0.155
1	mean	103	0.164
5	sum	125	0.197
5	mean	121	0.205

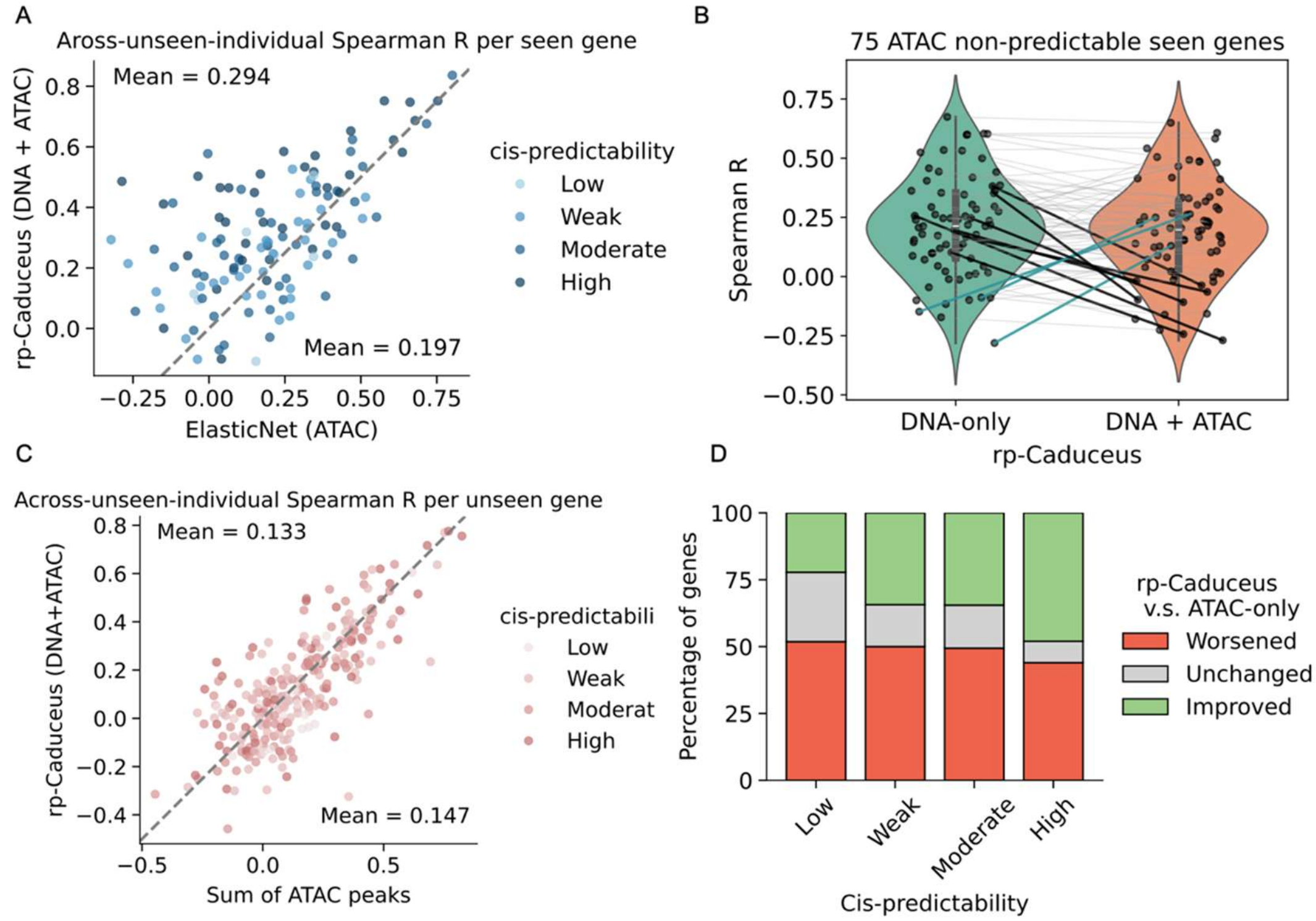

Figure 8 (added as Fig. S22). ATAC-only analyses in seen- and unseen-gene settings. A, Comparison between a supervised ATAC-only ElasticNet baseline and rp-Caduceus (DNA+ATAC) in the seen-gene setting. For each seen gene, the ATAC-only ElasticNet model was trained using ATAC features summarized within the same 64 kb TSS-centered context window used by rp-Caduceus. Noted that 125 out of 200 seen genes are valid for ATAC-only model. B, Performance of rp-Caduceus with DNA-only and DNA+ATAC inputs on 75 seen genes for which the ATAC-only ElasticNet baseline produced uninformative predictions. C, Comparison between an unsupervised ATAC-only baseline and rp-Caduceus (DNA+ATAC) in the unseen-gene setting. The ATAC-only score was computed by summing ATAC signal across the same 64 kb genomic context and correlating this score with observed expression across held-out individuals. Noted that 275 out of 287 unseen genes are valid for ATAC-only model. D, Stratification of unseen genes by cis-predictability. Genes were classified as improved, unchanged, or worsened according to the performance difference between rp-Caduceus (DNA+ATAC) and the ATAC-only baseline.

#2 ATAC-seq is often more expensive and difficult to collect than RNA-seq. If individual-level multi-omic data is already available, the need for a predictive model is diminished, as the target measurements (expression) are likely already accessible. What would need to be demonstrated is that the DNA + ATAC model reveals some insight about the underlying variants that other methods - such as PrediXcan - would not.

Response: We thank the reviewer for this important comment. Related to point 1, we agree that individual-level ATAC-seq is not generally easier to obtain than RNA-seq, and therefore the DNA+ATAC setting should not be interpreted as a practical replacement for transcriptome measurement when RNA-seq is already available. Our purpose in including ATAC-seq was to use chromatin accessibility as a regulatory-state intermediate to **diagnose the source of prediction failure**. The improvement observed after adding ATAC suggests that a

substantial limitation of DNA-only models is their incomplete ability to infer individual-specific regulatory states from sequence alone, rather than simply insufficient decoder capacity. One notable observation is that DNA plays a vital role in the few-shot setting (DNA+ATAC returns a big gain compared to ATAC-only; Fig. S22A), while in zero-shot learning, only ATAC contributes to the prediction and adding DNA did not make any contribution (Supp. Fig. S22C). Collectively, DNA and ATAC showed complementary roles in associating to gene expression (DNA: more informative but more complex, versus ATAC: moderately informative but straightforward to represent). We have further clarified this point in the revised manuscript (see the response to the above point 1).

#3 The authors write that: "Despite the limited power in a zero-shot prediction, xDecoder with DNA-only inputs showed promise in few-shot learning (Fig.2A), suggesting potential utility for applications involving rare or somatic variants where only a small number of fine-tuning individuals may be available" However, based on the results presented, few-shot learning from RNA seems to have the same problem as zero-shot learning with ATAC. Why not use PrediXcan instead? The authors mention that genes exist which PrediXcan gets wrong, but to justify a practical utility would require some additional analysis. Can we extract some insights about rare or somatic variants from these genes xDecoder gets right?

Response: We agree that PrediXcan is a strong and practical baseline when expression variation is well explained by common cis-eQTLs represented in the training cohort. Our goal is not to replace PrediXcan in this setting. Instead, xDecoder is motivated by a complementary regime. PrediXcan applies fixed gene-specific weights learned from variants observed or well tagged in a training panel, whereas a sequence-based model operates directly on DNA sequence and can, in principle, evaluate arbitrary sequence perturbations, including variants that are rare, novel, or absent from the eQTL training space. As shown in Fig. 2B, among genes for which PrediXcan provided no informative prediction, xDecoder-rp-Caduceus with DNA-only input recovered individual-level expression variation for 46 genes in the seen-gene setting. This result does not imply that xDecoder universally outperforms PrediXcan. Rather, it identifies a subset of genes where fixed eQTL weights are uninformative but sequence-based representations still contain predictive signals.

To further examine whether xDecoder can provide variant-level hypotheses in this setting, we performed an exploratory analysis of one representative gene, **AL160408.3**, from the 46 PrediXcan-unpredictable but xDecoder-predictable genes. For this gene, xDecoder-rp-Caduceus achieved a Spearman R = 0.385 between predicted and observed expression. We then evaluated all **1,893 known variants** within the xDecoder context window by in silico perturbation. Specifically, each variant allele was reverted to the reference allele while keeping the rest of the sequence unchanged, and the resulting change in predicted expression was used as the xDecoder perturbation score. The majority of variants had near-zero predicted effects, whereas a small subset showed larger effects. Several of these high-scoring variants overlapped known eQTL SNPs with large observed effect sizes, suggesting that xDecoder can nominate candidate cis-regulatory variants for genes where PrediXcan is uninformative. Importantly, this analysis is not restricted to variants assigned nonzero weights by a fixed eQTL model, but instead evaluates all observed sequence variants within the model context window. We emphasize that this analysis is exploratory and does not establish causal variant interpretation or direct rare/somatic variant utility, but it illustrates the complementary information that sequence-based modeling can provide beyond fixed eQTL-weight predictors such as PrediXcan.

We have revised the manuscript as:

(P4, line 10-P5 line 2): "*Notably, rp-Caduceus successfully modeled 46 genes that PrediXcan failed to predict—i.e., where PrediXcan predicted identical values across individuals (Fig. 2B).* ***This result identifies a subset of genes where fixed genotype weights are uninformative while sequence-based representations still contain predictive signal. As an exploratory follow-up, we performed variant-level perturbation analysis for one representative gene from this prediXcan unpredictable set and found that high-scoring xDecoder perturbations overlapped known eQTL SNPs with large observed effects (Fig. 2C), suggesting that xDecoder can generate variant-level hypotheses in such loci"***

(P5, lines 43-49): "*In summary, taking each gene as an independent output variable, we may view Test 1 as few-shot learning and Test 3 as zero-shot learning. Despite the limited power in a zero-shot prediction, xDecoder with DNA-only inputs showed promise in few-shot learning (Fig.2A).* ***Because sequence-based models operate directly on DNA sequence, they can in principle evaluate sequence perturbations outside the fixed variant-weight space of linear predictors, suggesting potential utility in settings where rare or novel variants weights are unavailable.*** *However, the inability of existing models to generalize across loci highlights a bottleneck of DNA–RNA fine-tuning alone.****"***

(P8, lines 4-10): "First, incorporating personal genome–expression pairs improves prediction for seen genes and can complement existing linear models such as PrediXcan. In particular, xDecoder identified a subset of genes for which PrediXcan produced no informative individual-level predictions, and exploratory perturbation analysis showed that the model can nominate candidate cis-regulatory variants within such loci (Fig. 2C). This suggests a potential path toward interpreting sequence perturbations beyond fixed common-variant eQTL weights, although direct validation on rare-variant and somatic-variant datasets remains necessary".

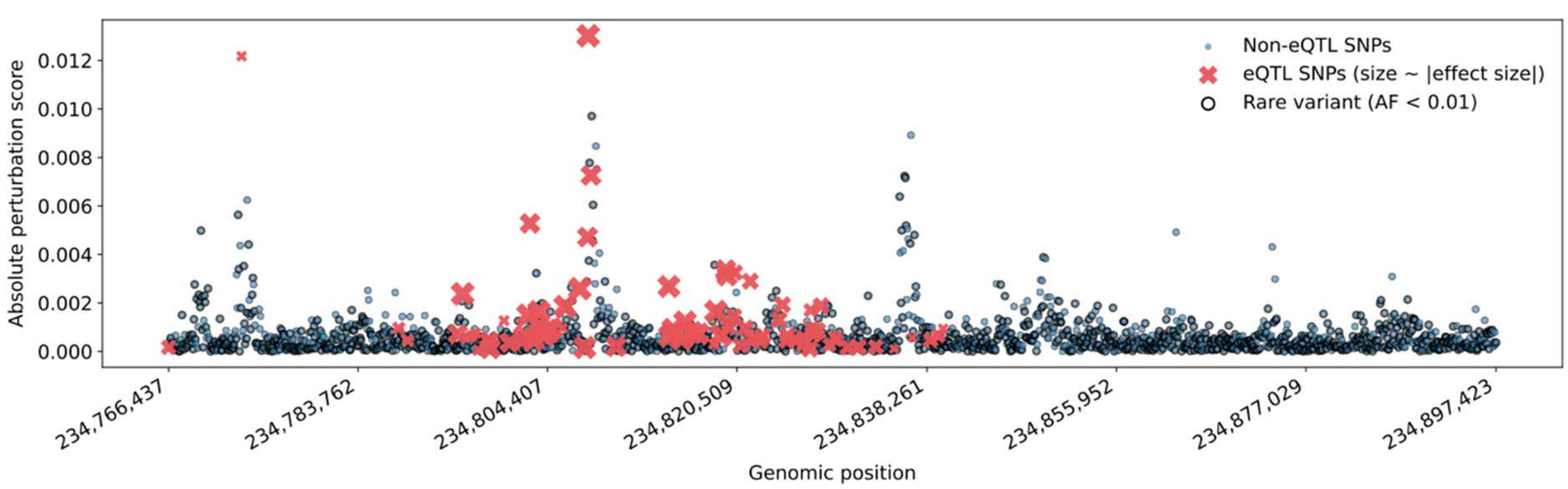

Figure 9 (added as Main. Fig. 2C). Variant-level perturbation analysis for a PrediXcan-uninformative gene AL160408.3.

#4 Fig 2A shows that DNA-LMs have essentially zero performance after reference training. Moreover, the performance of all three DNA-LM across unseen genes from seen individuals is very poor (Fig S1). However, the DNA-LMs then perform better than S2F models after

individual fine-tuning. It is difficult to make sense of these results. Do these DNA-LMs actually add anything to xDecoder besides providing a tokenization and (implicitly) a positional encoding? How does xDecoder perform if trained directly on one-hot sequence or another tokenization scheme (e.g. wordpiece or similar)?

Response: We thank the reviewer for this insightful question. First, Fig. S1 presents cross-gene correlation within individuals, which probes a different axis of generalization (gene variations) than Fig. 2A, where we evaluate cross-individual correlation per gene to assess personalized variation. Second, the stronger performance of S2F models under reference-only evaluation is expected: S2F encoders (Enformer, Borzoi, AlphaGenome) are trained with supervised functional targets in relevant cellular contexts, so they have been exposed to the gene expression labels of those genes during pretraining. By contrast, DNA language models are pretrained and self-supervised on sequence alone; although they “see” the same genomic sequence, they do not observe functional labels (e.g., gene expression), and thus are not expected to be competitive in this setting. However, **personalized prediction poses a different challenge**: the model must be sensitive to allelic variation and its regulatory consequences across individuals. In this setting, we find that xDecoder built on DNA-LM embeddings performs better after individual-level fine-tuning than xDecoder built on S2F embeddings. This result suggests that large-scale self-supervised DNA-LM representations contain useful information for modeling personal variation, and that this information can be more effectively adapted to variant-sensitive prediction through effective decoder. Admittedly, this paradox of capturing cross-gene versus cross-individual variability, while aligning with training strategies, remains unresolved, partly due to the different effects from sequence patterns and local variants.

As suggested, we additionally trained a vanilla xDecoder using one-hot encoded DNA sequence as input, with the same context length as Caduceus (131,070 bp) (Table 2 below). Overall, the one-hot model showed substantially weaker generalization than DNA-LM-based xDecoder in both unseen-gene and unseen-individual evaluations. This result indicates that the benefit of DNA-LMs is not limited to simple tokenization or positional encoding. Rather, their pretrained sequence representations provide a stronger starting point for learning personalized regulatory effects than raw one-hot sequence alone.

We have updated the main text as:

(P2, line 47 - P3, line 1): “In the cross-gene variability (row vector), xDecoder with S2F embeddings performs significantly better than gLM embedding (Fig. S2), as S2F encoders are trained with supervised functional targets (including expression-related readouts) at these loci in similar cellular contexts, whereas gLMs are pretrained self-supervised on sequence only without functional labels. Notably, xDecoder using one-hot embeddings showed substantially weaker generalization than gLM-based xDecoder. This suggests that the advantage of gLMs is driven less by the tokenization scheme itself and more by the sequence representations learned through self-supervised pretraining (Table S1).”

Table 2 (added as Table S1). Gene expression prediction performance (Mean Spearman R) across different training strategies for xDecoder with one-hot embeddings (context length: 131,070 bp). Task1: Across unseen individuals per seen gene (Test 1 in main Fig. 1B); Task2: Across unseen individuals per unseen gene (Test set 3 in main Fig. 1B); Task3: Across unseen genes per unseen individual (Test set 3 in main Fig. 1B).

Training strategy	Task 1	Task 2	Task 3
Ref-only	-0.016	0.0005	-0.015
Indiv-only	0.023	0.006	-0.047
Ref + indiv	0.003	0.006	0.007

#5 The study relies almost exclusively on Lymphoblastoid Cell Lines (LCLs) from the GEUVADIS dataset. Tissue-Specific Blindness: Most regulatory variation is context-dependent, with distinct enhancers driving expression in different tissues (e.g., heart vs. brain). A model benchmarked solely on immortalized LCLs avoids the core challenge of regulatory genomics: tissue-specific regulation.

Response: We thank the reviewer for raising this point. We agree that tissue-specific regulation is a central problem in regulatory genomics, but it is out of scope for the present study, which is designed to isolate a different axis of difficulty: personalized (cross-individual) expression variation within a fixed cellular context. Existing sequence-to-function models are primarily optimized for predicting population-average regulatory activity across diverse contexts, whereas our focus is on utilizing paired genome–transcriptome data to evaluate and model individual variation. We have revised the manuscript to clarify this scope and more importantly, we also would like to explore personalized, tissue-specific regulation for future work.

(P8, lines 29-31): "<u>Our current evaluation is limited to a single cellular context, lymphoblastoid cell lines, and extending this framework to multi-tissue settings and context-specific personalized regulation remains an important direction for future work.</u>"

#6 Borzoi and AlphaGenome both predict RNA-Seq expression directly; they are likely to perform better than CAGE for expression prediction. AlphaGenome also performs better with 1M basepair as input, you can crop for the target sequence length after getting the prediction.

Response: We thank the reviewer for pointing this out. We clarify that the AlphaGenome results reported in the main text use the model's full context length (1,048,576 bp). In addition, because AlphaGenome provides multiple output tracks in cell types closely related to our LCL setting (EFO:000572 and EFO:002784), and provided both RNA-seq and CAGE, we performed a comparison across settings. Specifically, we evaluated AlphaGenome using both a shorter context (131 kb), and compared CAGE- versus RNA-seq outputs around the TSS to select the most appropriate track/label for our benchmark. These results are presented in Table S1, and based on this analysis we report performance using the EFO:002784 track and the CAGE output in the manuscript.

Minor:

#7 The methods state that individual genomes were constructed by inserting SNPs in the reference genomes. Were individuals with indels in the intervals excluded or were those indels simply ignored? If so, is this not likely to depress performance if an indel is present in the individual but not seen by the model(s)? It would be good to know the frequency of such events in the GEUVADIS data and whether it impacts the presented metrics.

Response: We thank the reviewer for pointing it out. In response, we quantified the indels within 131,070 bp windows around the TSS for each of the 200 seen genes. We considered only those indels that were present in at least one individual within the 150 individuals (50 training and 100 testing individuals). As shown in Fig. 10A, most of the genomic sequences around the TSS contain fewer than 100 indels, which accounts for only ~0.07% of the total sequence variation. When we binned xDecoder (rp-Caduceus) performance by indel count (Fig. 10B), we observed no significant trend indicating that model performance is influenced by indel presence. We have updated the main text as:

(P5, lines 20-22): "Although we did not include INDELs in the personal sequence, we evaluated their potential impact and found that the number of INDELs is not related to the performance (Fig. S13)."

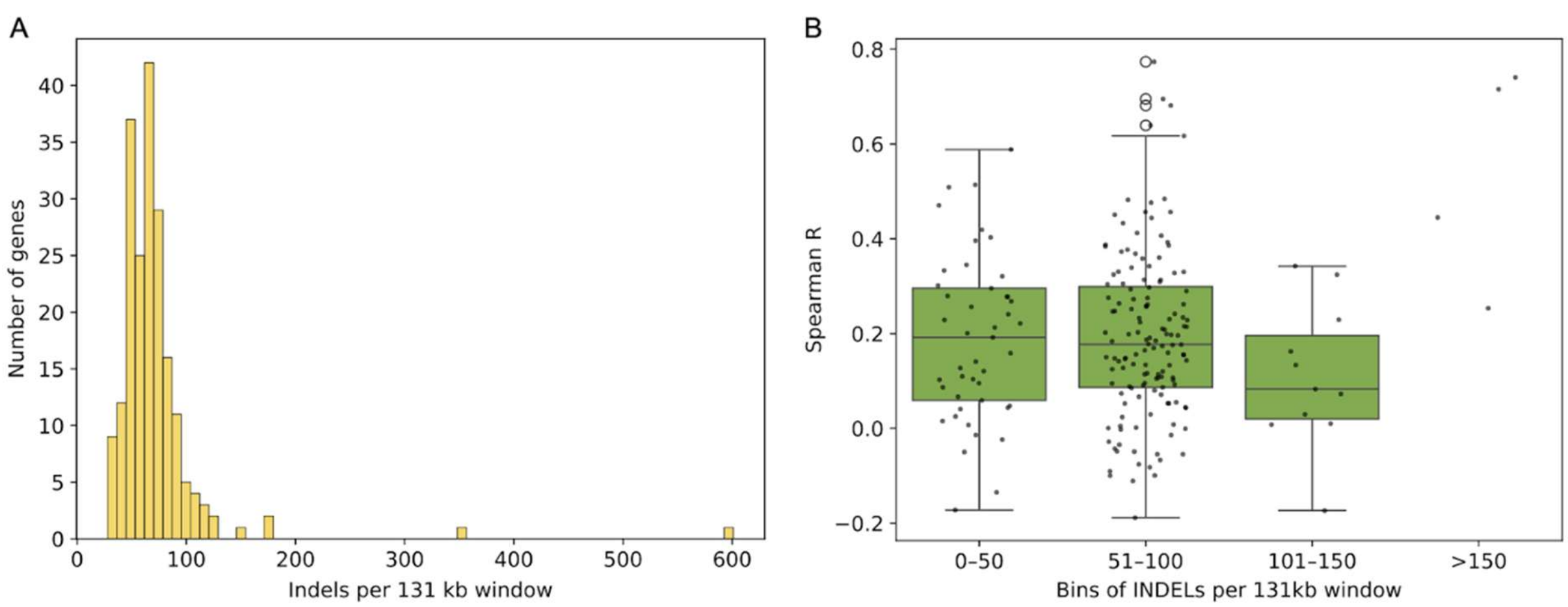

Figure 10 (added as Fig. S13). Impact of INDEL burden on model performance. (A) Distribution of the number of indels within 131,070 bp windows around the TSS for the 200 seen genes (Test 1, main Fig. 1B). (B) Performance of xDecoder (rp-Caduceus) binned by the number of indels in the 131,070 bp context around the TSS.

#8 "Genes with higher eQTL density tended to show improved correlation, and xDecoder (rp-Borzoi) achieved higher accuracy than the pretrained Borzoi model across all bins. These observations indicate longer receptive fields increase the probability of incorporating informative regulatory variants". This point is unclear. The methods indicate that Borzoi and rp-Borzoi have identical receptive fields, as the latter takes the embeddings of the former, so how does the performance increase of rp-Borzoi over Borzoi demonstrate anything about the receptive field?

Response: we thank the reviewer for pointing it out. To better clarify it, we have updated the main text as:

(P5, lines 15-18): *“Genes with higher eQTL density tended to show improved correlation,* ***consistent with the expectation that input context containing more informative cis-regulatory variants are easier to predict (Fig. 2D, Fig.S11).*** *In addition, xDecoder (rp-Borzoi) achieved higher accuracy than the pretrained Borzoi model across all bins.”*

#9 The placement of PrediXcan in figure 2D is confusing. I understand the intent, but since it does not correspond to either the x (where it is in between two categories) or y-axis (the scale goes to ~0.1, whereas the stated value is 0.216), it makes the figure hard to read.

Response: We agree with the reviewer. We removed PrediXcan from Fig. 2D to avoid confusion. And we now included the PrediXcan result in the main text as:

(P5, lines 31-32): “For context, PrediXcan trained separately for each gene achieves a mean Spearman correlation of 0.216.”

#10 There are four borzoi models. Could the authors please clarify if they used a particular of one of these, or averaged across all four.

Response: We thank the reviewer for pointing it out, we have updated the main texts as:

“(P10, line 12): We used the pretrained Enformer and Borzoi replicate-0 checkpoints from [22, 23], respectively. “

#11 Table 1: why is the number for ref-only, DNA+ATAC, task 2 in bold? It does not seem to be the highest in either the column or the row.

Response: We have updated it to avoid any misunderstandings.

#12 Fig S6: the caption mentions r-Caduceus and rp-Borzoi, but the legend has r-Caduceus and rp-Caduceus

Response: We have corrected it as “r-Caduceus and rp-Caduceus” in the caption.

#13 Flashzoi is cited twice, once the published version and once the bioRxiv. Is this intentional?

Response: We thank the reviewer for pointing out this error. We have deleted the redundant one.

Point-by-point responses to the comments of Reviewer #3:

The paper introduces xDecoder, a framework that uses existing DNA foundation models to predict individual-level gene expression differences. Using paired genome and RNA data from GEUVADIS (lymphoblastoid cell lines from 1000 Genomes individuals), the authors compare six pretrained models under three training setups: using the reference genome only, using individual genomes only, and pretraining followed by fine-tuning on individuals. The

main findings are: (1) Fine-tuning enables prediction of expression differences for genes seen during training, with more than a threefold improvement over pretrained models; (2) Predicting expression for completely unseen genes fails when using DNA alone; (3) Adding individual-level ATAC-seq data improves zero-shot performance. The main claim of the paper is that integrating additional molecular data may be necessary for robust personalized prediction, and that the bottleneck may lie less in model architecture than in the information content of DNA alone for cross-individual generalization.

The work has several strengths, including a sensible modeling strategy, a useful benchmarking framework applied consistently across six genomic foundation models, a clear demonstration of few-shot improvements, and careful validation analyses including cross-population evaluation and gene-level stratification. However, I have the following major concerns:

Response: We thank the reviewer for the thoughtful comments of our study and constructive suggestions.

#1 The improvement observed when integrating individual-level ATAC-seq is interesting, but it is unclear how much of this gain reflects true DNA×ATAC interaction learning versus simply conditioning on a measured regulatory phenotype that is already strongly correlated with expression. Because chromatin accessibility lies close to transcription in the regulatory cascade, the improvement could reflect access to an intermediate phenotype rather than enhanced genotype-aware modeling. To clarify attribution, the manuscript would benefit from including ATAC-only and reference-DNA+ATAC baselines in the unseen-gene setting. This would help disentangle whether the improvement stems primarily from chromatin accessibility itself or from genotype-aware modeling beyond accessibility.

Response: We thank the reviewer for this important point. We consider this point refer to the same with **Reviewer 2, major comment #1**. We agree that the improvement observed after adding individual-level ATAC-seq should not by itself be interpreted as evidence that the model has learned complex DNA×ATAC regulatory logic. Because chromatin accessibility is closely related to transcriptional state, ATAC may serve as a measured regulatory-state proxy for expression.

To address this point, we added ATAC-only control analyses. First, in the seen-gene setting, we evaluated whether simple ATAC-based models can predict individual-level expression. We constructed ATAC-only linear models using ATAC peaks within the same 64 kb TSS-centered window used by rp-Caduceus (DNA+ATAC). For the ATAC linear model, we tested to merge the peaks into 1 or 5 meta peaks, using sum-up or average strategy within the meta peak (Table 1). ElasticNet models were trained separately for each gene using the same 50 training and 44 testing individuals as in the rp-Caduceus (DNA+ATAC) experiments. The results are comparable to each other, and we select 5 meta peaks with sum-up to compare with xDecoder as it yields most valid predictions. Among these 125 ATAC-predictable genes, rp-Caduceus (DNA+ATAC) achieved a mean Spearman correlation of 0.294, compared with 0.197 for the ATAC-only linear baseline (Fig. 8A). Conversely, among genes for which the ATAC-only linear model produced uninformative predictions, xDecoder with DNA-only input achieved a mean Spearman correlation of 0.212, compared with 0.186 for DNA+ATAC, indicating that the contribution of ATAC is gene-dependent and that some seen-gene

predictions are better captured by sequence-derived signal than by the simple ATAC proxy (Fig. 8B).

Second, in the zero-shot unseen-gene setting, we implemented a simple ATAC-only baseline by summing ATAC signal across the same 64 kb genomic context around TSS and correlating this score with observed expression across held-out individuals. This baseline yielded valid predictions for 275 of 287 genes. Among these genes, the summed ATAC-only baseline achieved a mean Spearman correlation of 0.147, compared with 0.133 for rp-Caduceus (DNA+ATAC) on the same set (Fig. 8C). This result supports the point that ATAC can act as a strong proxy for expression, and indicates that the aggregate improvement of DNA+ATAC over DNA-only in the unseen-gene setting is largely attributable to measured ATAC signal rather than to personalized DNA sequence alone.

We ask whether DNA provides any additional information once ATAC is available, we stratified genes by PrediXcan cis-predictability and compared rp-Caduceus (DNA+ATAC) with the ATAC-only baseline. We computed ΔR by the difference between rp-Caduceus (DNA+ATAC) and ATAC-only, and classified genes as improved, unchanged, or worsened using a threshold of $\Delta R>0.02$, $-0.02<=\Delta R<=0.02$, $\Delta R<-0.02$. This analysis showed that rp-Caduceus (DNA+ATAC) provided greater relative benefit for genes with stronger cis-predictability, whereas the ATAC-only baseline was more competitive for genes with weak cis signal (Fig. 8D). This suggests that personalized DNA contributes most when expression variation has a detectable cis-genetic component, while ATAC alone can be a strong expression proxy for genes with weak cis-genetic signals.

Based on these analyses, we revised the manuscript to make the interpretation of the DNA+ATAC experiment more cautious. To clarify, we do not present DNA+ATAC prediction as a practical replacement for RNA-seq, nor as definitive evidence that the model learns regulatory logic beyond measured chromatin accessibility. Instead, we interpret the ATAC experiment as diagnostic: the large improvement from adding measured ATAC indicates that DNA-only models currently fail to infer individual-specific regulatory states from sequence alone. This motivates future work in which multi-omic measurements such as ATAC-seq may be used as auxiliary supervision or intermediate labels to improve DNA-only models, rather than necessarily as required inputs at prediction time. We have revised the manuscript as:

(P7, lines 3-38): ***<u>"To distinguish whether the improvement from adding ATAC reflects model learning beyond a simple chromatin-accessibility proxy, we added ATAC-only analyses tailored to the two evaluation settings. In the seen-gene setting, RNA labels for the target genes are available during training, so we constructed a supervised ATAC-only baseline by training per-gene ElasticNet models using ATAC features summarized within the same 64 kb TSS-centered window used by rp-Caduceus. Specifically, ATAC peaks within the context window were aggregated into five regional features and used to predict expression across individuals under the same training and testing split. This baseline asks whether a simple gene-specific mapping from measured accessibility to expression is sufficient. In this setting, rp-Caduceus (DNA+ATAC) outperformed the ATAC-only ElasticNet baseline （0.294 vs 0.197 for 125 ATAC predictable genes, Fig.S22A), indicating that the few-shot model is not merely reproducing a supervised accessibility–expression proxy. Rather, paired genome–transcriptome training appears to learn gene-specific individual-level variation beyond</u>***

what is captured by this ATAC-only linear model. Specifically, for 75 ATAC-non predictable genes, rp-Caduceus (DNA-only) outperforms rp-Caduceus (DNA+ATAC), indicating some seen-gene predictions are better captured by sequence-derived signal than by the simple ATAC proxy (Fig. S22B).

In the unseen-gene setting, a supervised per-gene ATAC model is not applicable because RNA labels from the held-out genes are not available during training. We therefore used a simple unsupervised ATAC-only gene-activity baseline by summing ATAC signal across the same 64 kb genomic context and correlating this score with observed expression across held-out individuals. This summed-ATAC baseline was competitive with rp-Caduceus (DNA+ATAC), supporting that measured ATAC can serve as a strong proxy for expression (Fig. S22C, Table 1). Stratification by cis-predictability further showed that the relative benefit of rp-Caduceus (DNA+ATAC) over ATAC-only was more evident for genes with stronger cis-genetic signal, whereas ATAC alone was more competitive for genes with weak cis-genetic signal (Fig. S22D). Nevertheless, both ATAC-only and DNA+ATAC models remained below the per-gene trained PrediXcan reference (Fig. S19B). Together, these results suggest that individual-level chromatin accessibility is critical for predicting personal gene expression at unseen genomic contexts. The competitive performance of the ATAC-only baseline indicates that measured ATAC provides regulatory-state information that is highly informative for expression variation. At the same time, the poor performance of DNA-only models in this setting suggests that current sequence models do not yet reliably infer individual-specific chromatin states from DNA sequence alone. Consistently, AlphaGenome-predicted gene-level ATAC signals showed only modest concordance with observed ATAC signals across individuals (mean Spearman R = 0.168; Fig. S23A), which may partly explain the limited zero-shot generalization of DNA-only models across loci (Fig.S23B). Therefore, rather than treating DNA+ATAC as a practical replacement for RNA-seq, we interpret this experiment as revealing a key bottleneck of current DNA-only models. A promising future direction is to use individual-level multi-omic data, including ATAC-seq, as auxiliary supervision or intermediate regulatory-state targets during training, with the goal of improving DNA-based prediction at unseen loci.”

#2 The manuscript clearly demonstrates that DNA-only models fail in the unseen-gene setting, but it does not examine whether this failure is uniform or gene-dependent. It would be valuable to understand whether zero-shot prediction breaks down systematically, or whether certain classes of genes (e.g., high cis-heritability, dense eQTL architecture, promoter-proximal regulation) show partial transfer. A simple stratified analysis by gene-level properties (e.g., eQTL density, regulatory complexity, or heritability proxies) could provide insight into when zero-shot prediction partially succeeds and what biological features limit generalization. This would move the work beyond documenting failure toward identifying mechanistic constraints on sequence-based generalization.

Response: We thank the reviewer for this constructive suggestion. We consider this point refer to the same with **Reviewer 1 comment #3**. We agree that the failure of DNA-only zero-shot prediction should not be treated as uniform across all genes, and that stratifying genes by cis-regulatory properties can provide insight into when partial transfer is possible.

To compare the zero-shot results with an estimate of cis-explainable signal, we computed a PrediXcan-style gene-specific linear predictor for each of the 287 unseen genes using all available training individuals (n = 362). We use the PrediXcan Spearman correlation as a cis-predictability indicator. We then stratified the 287 genes into four groups based on PrediXcan cis-predictability: low (R < 0.10), weak (0.10 ≤ R < 0.25), moderate (0.25 ≤ R < 0.40), and high (R ≥ 0.40) (Fig. 5A). Across models, zero-shot performance increases with cis-predictability—most notably for AlphaGenome—yet remains substantially below the gene-specific supervised reference, even for moderate and high cis-predictablity genes (Fig. 5BC), highlighting the difficulty of transferring cis-regulatory rules across loci. Conversely, genes with low/weak cis-predictability might have a larger contribution from trans-regulatory and context effects.

These results indicate that the zero-shot failure is gene-dependent rather than completely uniform. Genes with stronger cis-regulatory signal show partial transfer, whereas genes with weak cis-predictability may be more strongly influenced by trans effects, environmental factors, or regulatory mechanisms not captured by local DNA sequence. At the same time, the persistent gap relative to gene-specific supervised models suggests that current DNA-only foundation models still have limited ability to transfer cis-regulatory rules across loci. We have added this analysis and corresponding discussion to the revised manuscript：

(P5, lines 32-36): "We further stratified the unseen genes by PrediXcan cis-predictability (Methods;Fig. S16A). It shows zero-shot performance tended to increase for genes with stronger cis signal—mostnotably for AlphaGenome—yet remained substantially below the gene-specific supervised PrediXcan reference even among moderately/highly cis-predictable genes (Fig. S16B, C), highlighting the difficulty of transferring cis-regulatory rules across loci. "

#3 The reduced performance when training on European individuals and evaluating on Yoruba individuals suggests possible reliance on population-specific LD structure rather than learning ancestry-robust regulatory principles. This is an important result that should be analyzed more explicitly, particularly given the implications for portability. Even a limited diagnostic analysis-such as relating performance drop to allele frequency shifts, LD decay, or variant novelty rates, or clarifying whether mixed-ancestry training alters the outcome-would significantly strengthen interpretation.

Response: We thank the reviewer for this constructive suggestion and agree that the reduced performance should be further investigated. To address this point, we added a diagnostic analysis to examine whether the EUR-to-YRI performance drop is associated with allele-frequency differences, variant novelty, or LD-structure differences.

To make the comparison better aligned with the split-population experiment, we used the model trained on 50 European individuals and evaluated it on two test sets: the 89 YRI individuals used in the split-population setting (the results presented in main Fig.2A), and 90 European individuals extracted from the original mixed test set (Test 1, main Fig.1B) after excluding YRI samples. Thus, the trained model was kept fixed, which we adopted rp-Caduceus here, and only the ancestry composition of the evaluation samples was changed. As shown in Fig. 11A, the model achieved higher average cross-individual prediction

performance in European test individuals than in YRI test individuals, with mean Spearman correlations of 0.184 and 0.118, respectively.

We then examined three gene-level indicators that may contribute to the cross-ancestry performance gap. First, we calculated the novel variant rate per gene, defined as the fraction of variants that were common in one population, using MAF ≥ 0.05, but absent in the other population within the model input context of each gene. In this analysis, the input context was 131,070 bp around each gene. Second, we calculated the average allele-frequency shift per gene, defined as the mean absolute allele-frequency difference between European and YRI individuals across variants in the same input region. Third, we calculated LD-structure similarity between European and YRI individuals. Specifically, for each gene region, we separately identified pairs of variants in strong LD, defined as r2 >0.8, in the European and YRI populations, and then measured the overlap between the two sets of strong-LD variant pairs using a Jaccard index. A higher Jaccard index indicates more similar LD structure between the two populations, whereas a lower value indicates more ancestry-specific LD organization.

This analysis showed that the cross-ancestry performance gap could not be explained by a single factor. Genes with larger performance gaps tended to have higher novel variant rates and lower LD-structure similarity, suggesting that both unseen ancestry-specific variants and population-specific LD organization may contribute to reduced portability (Fig. 11B,D,E). In contrast, average allele-frequency shift showed a weaker association with the performance gap (Fig. 11C). One possible explanation is that averaging allele-frequency differences across all variants in the input region may dilute the contribution of a small number of functionally relevant regulatory variants. Consistently, when genes were grouped by LD-structure similarity, the median performance gap decreased in bins with higher LD similarity, further supporting ancestry-specific LD structure as one contributor to the reduced YRI performance.

We have revised the manuscript as:

(P5 lines 3-14): *“We next evaluated the cross-population generalization using a population-split experiment, training on 50 European individuals* ***and evaluating the same trained model on two held-out test sets: 89 Yoruba individuals from Ibadan (YRI) and 90 European individuals extracted from the original mixed test set after excluding YRI samples.*** *In this setting, the benefit of individual training decreased (Fig. 2A, Fig.S10A), reflecting the known challenge of cross-population transfer [5, 16–18].* ***To investigate potential factors to this performance gap, we examined three gene-level indicators within the 131,070 bp rp-Caduceus input context: novel variant rate, mean allele-frequency shift, and LD-structure similarity between European and YRI individuals (Methods). No single factor fully explained the cross-ancestry performance drop. However, genes with larger EUR-to-YRI performance gaps tended to have higher novel variant rates and lower LD-structure similarity, whereas average allele-frequency shift showed a weaker association (Fig. S10B-E). These results suggest that reduced cross-ancestry portability may arise from a combination of ancestry-specific variants and differences in local LD organization.”***

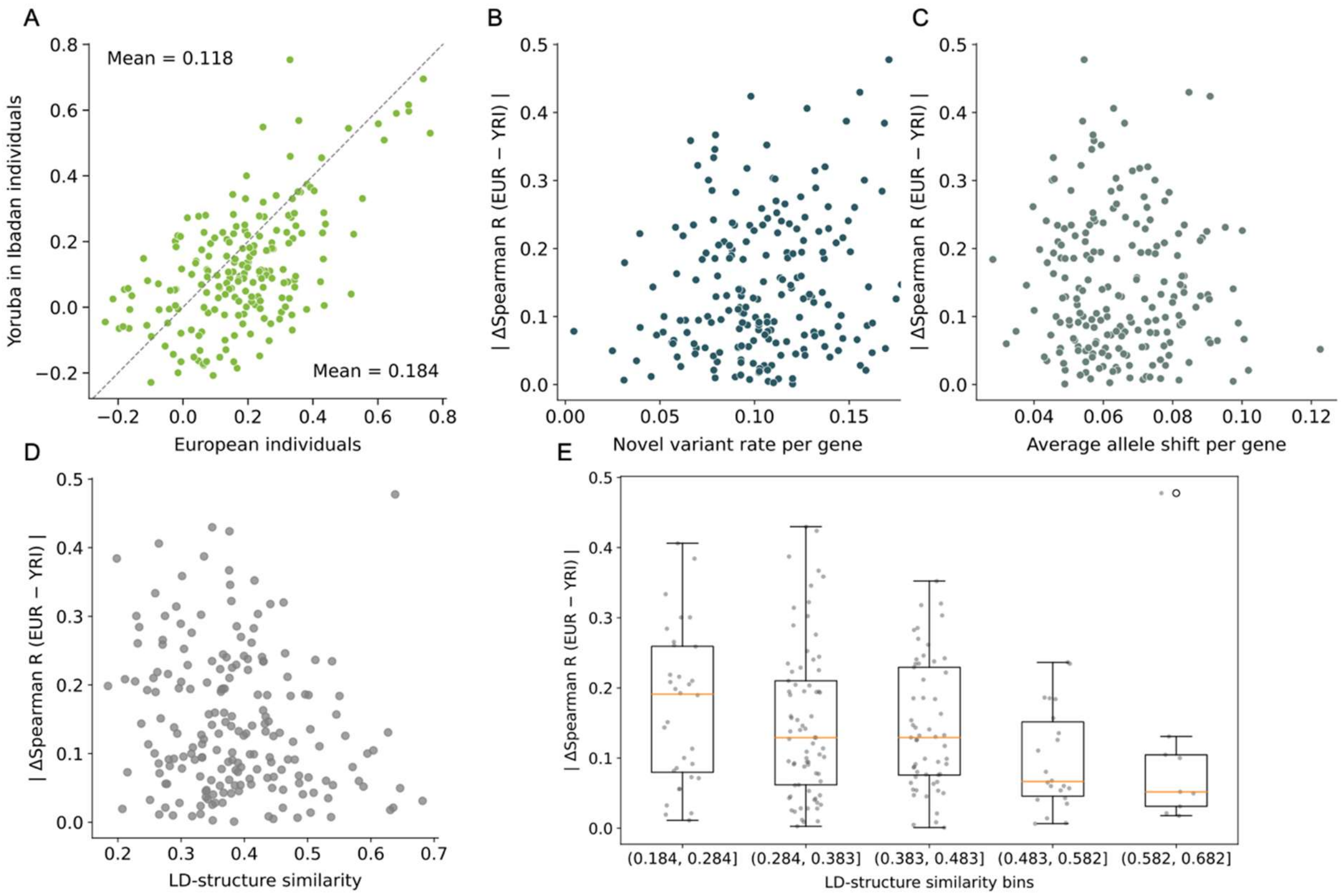

Figure 11 (added as Fig. S10). Analysis of cross-ancestry performance using the rp-Caduceus model. A, Gene-level prediction performance of the same European-trained model evaluated on European and YRI test individuals. B, Relationship between the absolute cross-ancestry performance gap and two variant-distribution indicators: B, novel variant rate per gene, defined as the fraction of variants with MAF ≥ 0.05 in one population but absent in the other population within the 131,070 bp around TSS; and C, average allele-frequency shift per gene, defined as the mean absolute allele-frequency difference between European and YRI individuals across variants in the same region. D, Relationship between the absolute performance gap and LD-structure similarity. LD-structure similarity was measured as the Jaccard index between strong-LD variant-pair sets identified separately in European and YRI populations obtained from ensembl 1KG panel, using R2>0.8. Higher values indicate more similar LD organization between populations. E, Distribution of the absolute performance gap after grouping genes by LD-structure similarity.

#4 Fine-tuning relies on 50 training individuals, but it is unclear whether performance has saturated at this scale. A simple learning curve analysis (e.g., training with 10, 20, 40, and 50 individuals) would help determine whether improvements plateau or remain data-limited. Even if additional data are unavailable, a brief discussion of expected scaling behavior would help contextualize the current results and clarify whether the limitation is architectural or statistical.

Response: We thank the reviewer for this suggestion. We have performed a learning-curve analysis by training with increasing numbers of individuals and report the results in Fig. S15. Specifically, we subsampled the training cohort to {50, 100, 150, 200} {50,100,150,200} individuals and evaluated performance under the three tasks in Fig. 1B. We observe that

increasing the number of training individuals yields consistent gains for few-shot prediction on seen genes (Fig. S15A) and improves generalization across unseen genes per unseen individual (Fig. S15B). In contrast, zero-shot prediction on unseen genes across unseen individuals remains near zero across all training sizes (Fig. S15C), indicating that the failure in this setting is not simply due to limited training sample size but reflects a more fundamental limitation in cross-gene transfer from DNA-only inputs. To better clarify it, we have clarified this in the revised manuscript as:

(P5, lines 25-31): "We further examined whether performance is limited by training size by increasing numbers of training individuals (n = 50, 100, 150, 200). Performance improves with larger training sets for seen-gene prediction across unseen individuals (Fig. S15A) and for cross-gene generalization within unseen individuals (Fig. S15B), with diminishing returns at higher n. In contrast, zero-shot prediction on unseen genes for unseen individuals remains near zero across all training sizes (Fig. S15C), indicating that the zero-shot bottleneck is not simply due to limited training individuals."

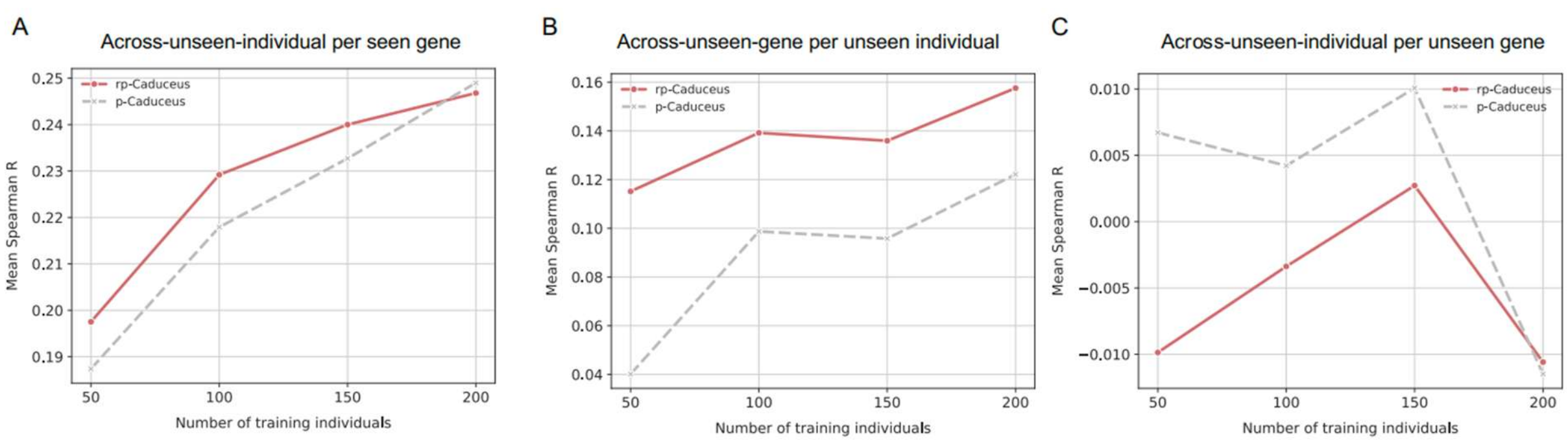

Figure 12 (As Fig. S15) Training-set scaling for xDecoder using Caduceus embeddings. Mean Spearman correlation as a function of the number of training individuals (n=50,100,150,200). Caduceus input length was capped at 64 kb for computational feasibility. (A), Across-individual correlation for seen genes. (B), Across-gene correlation for unseen individuals. (C), Across- individual correlation for unseen genes.

Minor points:
#5 The manuscript relies exclusively on lymphoblastoid cell lines (LCLs). While reasonable given data availability, the discussion should more explicitly acknowledge that conclusions may be cell-type specific and outline expectations for other tissues.

Response: We thank the reviewer for pointing it out. In response, we have updated the discussion as:

(P8, lines 29-31): "Our current evaluation is limited to a single cellular context, lymphoblastoid cell lines, and extending this framework to multi-tissue settings and context-specific personalized regulation remains an important direction for future work"

#6 The ATAC experiment uses 94 overlapping samples. The sample size should be clearly stated in the main text, and uncertainty estimates (e.g., confidence intervals) would help contextualize robustness.

Response: Thanks for this useful comment. We have computed the confidence intervals by bootstrapping and updated the main texts and Table 1 as:

(P6, lines 4-7): *"As a proof of concept, we incorporated individual-level ATAC-seq tracks into Caduceus embeddings by adding a base-resolution ATAC channel and trained the models using the same strategy as described above,* ***with 50 individuals for training and 44 for evaluation*** **and a 64kb sequence window centered on the TSS.** *(Methods, Table 1)."*

Table 1 Gene expression prediction performance (Mean Spearman R) across different training strategies and input modalities for Caduceus and ATAC-only. Values are reported as mean [95% CI], where confidence intervals were obtained by bootstrap resampling of genes (B = 10,000). Task1: Across unseen individuals per seen gene (Test set 1 in Fig. 1B); Task2: Across unseen individuals per unseen gene (Test set 3 in Fig. 1B); Task3: Across unseen genes per unseen individual (Test set 3 in Fig. 1B).

Training strategy	Input	Task 1	Task 2	Task 3
Ref-only	DNA+ATAC	0.066 [0.035, 0.097]	0.108 [0.082, 0.134]	0.343 [0.340, 0.347]
	DNA only	0.017 [-0.014, 0.047]	0.010 [-0.015, 0.035]	0.121 [0.118, 0.124]
Indiv-only	DNA+ATAC	0.222 [0.19, 0.254]	0.052 [0.030, 0.076]	0.242 [0.237, 0.246]
	DNA only	0.260 [0.231, 0.291]	-0.001 [-0.027, 0.025]	0.066 [0.062, 0.07]
Ref + Indiv	DNA+ATAC	**0.248** [0.218, 0.279]	0.129 [0.103, 0.155]	**0.365** [0.361, 0.370]
	DNA only	0.241 [0.209, 0.273]	-0.003 [-0.03, 0.023]	0.160 [0.156, 0.163]
Sum of ATAC	ATAC	0.123 [0.096, 0.151]	**0.140 [0.115, 0.166]**	NA

For comparability, ATAC-only predictions that were identical across individuals were assigned a Spearman R of 0.

#7 The paper "Flashzoi: an enhanced borzoi for accelerated genomic analysis" appears to be cited twice; the redundant citation should be removed.

Response: we thank the reviewer for pointing it out. The redundant citation was removed.

#8 Fig. 1A contains a typo ("Caudcues" instead of "Caduceus").

Response: We thank the reviewer for pointing it out and we have corrected the typo correspondingly.

24th Jun 2026

Manuscript Number: MSB-2025-13544R
Title: xDecoder unlocks the potential of genomic foundation models for few-shot personal gene expression prediction

Dear Dr. Huang,

Thank you for the submission of your revised manuscript to Molecular Systems Biology. Based on the reviewer feedback and additional advice from an expert advisor, I am pleased to inform you that we will be able to accept your manuscript pending the following final amendments and appropriate response to all remaining comments of the reviewers. Please note that to ensure accuracy of the responses to the reviewers' comments, we may have a reviewer or advisor look over your responses and revisions.

1) We require a complete account in our submission system for corresponding authors - currently the institutional address, ZIP code, phone number are missing for Dr. Huang.
2) Please note that we now mandate that all corresponding authors list an ORCID digital identifier. Currently an ORCID ID is missing for Dr. Ruibang Luo. An email with instructions to Dr. Luo was sent on May 15th, 2026. We encourage all authors to supply an ORCID identifier, which will be linked to their name for unambiguous name identification.
3) Please format the Data availability section according to the example below.
"The datasets and computer code produced in this study are available in the following databases:
- Chip-Seq data: Gene Expression Omnibus GSE46748 (https://www.ncbi.nlm.nih.gov/geo/query/acc.cgi?acc=GSE46748)
- Modeling computer scripts: GitHub (https://github.com/SysBioChalmers/GECKO/releases/tag/v1.0)
- [data type]: [full name of the resource] [accession number/identifier] ([doi or URL or identifiers.org/DATABASE:ACCESSION])"
4) Please note that the Data Availability section should only include data and code that have been generated in the course of the current study. For any publicly available data that have been reanalyzed, please move this information to the Methods and potentially include a data citation (see next point).
5) Our journal encourages inclusion of *data citations in the reference list* to directly cite datasets that were re-used and obtained from public databases. Data citations in the article text are distinct from normal bibliographical citations and should directly link to the database records from which the data can be accessed. In the main text, data citations are formatted as follows: "Data ref: Smith et al, 2001" or "Data ref: NCBI Sequence Read Archive PRJNA342805, 2017". In the Reference list, data citations must be labeled with "[DATASET]". A data reference must provide the database name, accession number/identifiers and a resolvable link to the landing page from which the data can be accessed at the end of the reference. Further instructions are available at: https://link.springer.com/journal/44320/submission-guidelines#cms-Reference-guidelines
6) Please rename "Competing Interests" to "Disclosure and competing interests statement" and ensure that this is a separate section of the manuscript. We updated our journal's competing interests policy in January 2022 and request authors to consider both actual and perceived competing interests. Please review the policy https://link.springer.com/partners/embo-press/editorial-policies#Competing%20interest%20disclosures and update your competing interests if necessary.
7) Please remove the author contributions from the manuscript and specify author contributions in our submission system. CRediT has replaced the traditional author contributions section because it offers a systematic machine-readable author contributions format that allows for more effective research assessment. You are encouraged to use the free text boxes beneath each contributing author's name to add specific details on the author's contribution. More information is available in our guide to authors: https://link.springer.com/journal/44320/submission-guidelines#cms-Manuscript-organisation-and-formatting
8) Please note that funding information should be given in an "Acknowledgements" section. Please ensure that all funding sources are entered into the manuscript submission system the same as in the manuscript.
9) Please remove the "Declarations" and "Supplementary information" sections of the manuscript. In addition, the "Background" section should be renamed to "Introduction".
10) Please correct the reference citation in the reference list to be alphabetical (not numerical). Where there are more than 10 authors on a paper, only the first 10 should be listed, followed by "et al.". DOIs should only be used for preprints and datasets that have not been published yet. Please check "Author Guidelines" for more information: https://link.springer.com/journal/44320/submission-guidelines#cms-Reference-guidelines
11) In the Methods, please take care of the following:
- The Materials and Methods section should be renamed to "Methods".
12) Please place individual sections of the manuscript in the following order: Title page - Abstract & Keywords - Introduction - Results - Discussion - Methods - Data Availability - Acknowledgements - Disclosure and Competing Interests Statement - References - Figure Legends - (Main Tables with legends if applicable) - Expanded View Figure Legends.
13) For the figures and figure legends, please take care of the following:
- Please remove all figures from main manuscript file and leave only main figure legends placed after the references. Main and EV figures should be uploaded as individual, high-resolution files. Please check "Author Guidelines" for more information: https://link.springer.com/journal/44320/submission-guidelines#cms-Expanded-View-data
- Please make sure to update the callouts of all figures in the main manuscript text such that they appeal sequentially. In addition, currently figure callouts are missing for the panels of the Appendix figures and Table S3.

- Please note that the exact p values are not provided in the legends of figures 2A, D
- Please note that the box plots need to be defined in terms of minima, maxima, centre, bounds of box and whiskers, and percentile in the legends of figures 2D, 3A
- Please note that information related to n is missing in the legend of figure 2D
- Please note that the measure of center for the error bars needs to be defined in the legend of figure 2A
14) The title page of the Appendix should contain "Appendix for + manuscript title" (author and affiliation list not necessary) and a table of contents with the page numbers for the listed items. The nomenclature should be Appendix Figure Sx throughout the manuscript and Appendix PDF.
15) Synopsis:
- Synopsis image: Please provide a graphic that summarises the main findings of the manuscript on a glance and upload it as a high-resolution jpeg file 550 pixels wide x 300 pixels high.
- Synopsis text: Please provide a separate word document including a short standfirst (maximum of 300 characters, including spaces) and up to 5 bullet points to summarise the key NEW findings. They should be designed to be complementary to the abstract - i.e. not repeat the same text. We encourage inclusion of key acronyms and quantitative information (maximum of 30 words / bullet point). Please use the passive voice.
- Please check your synopsis text and image before submission with your revised manuscript. Please be aware that in the proof stage minor corrections only are allowed (e.g., typos).
16) Source Data should be organized as a single source data file (zipped) per figure for main figures, with the panels clearly visible in the folder structure instead of a single excel file for all Source Data. e.g. all the Source data files for figure 1 need to be saved in a single folder and this needs to be zipped and then uploaded as "SD figure 1.zip" file.
17) As part of the EMBO Publications transparent editorial process initiative (see our policy here: https://www.embopress.org/transparent-process#Review_Process), Molecular Systems Biology will publish online a Peer Review File (PRF) to accompany accepted manuscripts. This file will be published in conjunction with your paper and will include the anonymous referee reports, your point-by-point response and all pertinent correspondence relating to the manuscript. Let us know whether you agree with the publication of the PRF and as here, if you want to remove or not any figures from it prior to publication. Please note that the Authors checklist will be published at the end of the PRF.
18) After your paper is published, we may promote it on social media. If you have any handles or hashtags for Bluesky you would like included, please let us know.
19) Please provide a point-by-point letter INCLUDING my comments as well as the reviewer's reports and your detailed responses (as Word file).

I look forward to reading a new revised version of your manuscript as soon as possible.

Yours sincerely,

Poonam Bheda, PhD
Scientific Editor
Molecular Systems Biology

Reviewer #1:

The authors have addressed my previous concerns well, and the revised manuscript more clearly demonstrates the advantage of xDecoder over existing sequence-based models. However, I remain somewhat concerned that the absolute predictive performance is still modest. In many settings, the reported Spearman correlations remain well below 0.5, and zero-shot generalization to unseen genes appears to remain very limited. For instance, the authors report improvements in the few-shot seen-gene setting, but the mean Spearman correlations are still modest, for example around 0.194 for rp-Caduceus versus 0.153 for rp-Borzoi in one main comparison. This raises questions about how reliable the model would be for practical personal gene expression prediction, especially at loci not included in training or in settings without matched regulatory data.

I therefore encourage the authors to discuss the limitations of xDecoder more explicitly. In particular, it would be useful to clarify that, although xDecoder improves upon current models, the current performance may still be insufficient for accurate gene-level prediction in many practical applications. The authors may also wish to discuss what levels of predictive accuracy would be needed for downstream biological interpretation, variant prioritization, or clinical relevance, and how future model development or additional multi-omic data might help close this gap.

Reviewer #2:

I appreciate the authors' transparency and the extensive new analyses provided in the revision. The addition of the ATAC-only baselines is very helpful in clarifying the model's behaviour. For 'seen' genes, the results demonstrate that combining DNA and ATAC yields slightly better predictions than ATAC alone. Whereas for 'unseen' genes (zero-shot learning), the results show that the summed ATAC-only baseline performs competitively with the DNA+ATAC model. This indicates that the DNA sequence does not currently provide additional predictive value beyond the ATAC data in a zero-shot context. For clarity, I think ATAC alone model should be shown in the main figure to highlight this limitation.
The rest of my concerns are addressed adequately.

Reviewer #3:

We thank the authors for their thorough and constructive revisions. The additional analyses address the core technical concerns, yet I feel the manuscript has not been consistently updated to reflect the implications of these new results, and thus requires further revision.
Below we assess each major point with particular attention to alignment between results and interpretation:
1) Concern #1 questioned whether the ATAC gain reflected genuine DNA×ATAC interaction learning versus conditioning on a regulatory proxy. The authors replied to this by adding supervised and unsupervised ATAC-only baselines, which show that the zero-shot improvement is largely attributable to measured ATAC rather than integrative modeling.

We appreciate the additional ATAC-only analyses. However, given that (i) in the seen-gene setting DNA+ATAC is not meaningfully different from DNA-only, and (ii) in the unseen-gene setting ATAC-only matches or exceeds DNA+ATAC, the current framing may still over-attribute the improvement to integrative modeling rather than to the inclusion of a measured regulatory phenotype. For example, the statement that "this bottleneck can be partially overcome by integrating individual-level chromatin accessibility" implies that integration itself overcomes a sequence-model limitation, whereas the ATAC-only controls indicate that the observed gain largely reflects the inclusion of a measured regulatory phenotype rather than integrative modeling. We therefore ask the authors either to revise the abstract and conclusions to explicitly align the claims with these results, or to provide a clear justification for why the current statements remain supported despite the ATAC-only controls.

More broadly, clarifying this point will help position the manuscript's true contribution (namely the few-shot seen-gene result and the diagnostic identification of the zero-shot bottleneck) without overstating the role of multi-omic integration.

2) Concern #2 questioned whether zero-shot failure was uniform or gene-dependent, and suggested stratifying by gene-level regulatory properties. The authors addressed this by stratifying unseen genes by PrediXcan-based cis-predictability and showing that zero-shot performance increases with stronger cis signal, although it remains below gene-specific supervised models.

We appreciate this analysis, which we think suggests that zero-shot performance is gene-dependent rather than uniformly absent. The monotonic improvement with cis-heritability indicates that the models capture biologically meaningful cis signal and that cross-locus transfer is not uniformly broken. In particular, pretrained S2F models (e.g., AlphaGenome) show measurable average signal in moderate/high cis strata. At the same time, the substantial gap relative to the supervised PrediXcan reference and the large within-bin variability indicate that prediction remains unreliable at the per-gene level.

We therefore suggest revising the framing to reflect both aspects: zero-shot performance is gene-dependent and partially recoverable for strongly cis-regulated genes, yet remains inconsistent and markedly inferior to gene-specific supervised models. The authors may of course disagree with this interpretation and are welcome to clarify why they consider the current framing of "failure" to be more appropriate in light of the stratified results.
3) Concern #3 questioned whether the cross-ancestry performance drop reflected reliance on population-specific LD structure and requested mechanistic diagnostics (e.g., allele frequency shifts, variant novelty, LD differences). The authors addressed this by adding gene-level analyses of novel variant rate, allele-frequency shift, and LD-structure similarity, showing that no single factor fully explains the EUR-to-YRI performance gap, although larger gaps are associated with higher variant novelty and lower LD similarity.

We appreciate the added diagnostic analyses. However, although the results are explicitly inconclusive, the current framing still places interpretive emphasis on LD-structure differences, which risks overstating the explanatory strength of the analysis.

We therefore suggest either (i) strengthening the analysis to more directly support a mechanistic claim regarding LD reliance (e.g., clearer quantification of effect sizes or multivariate modeling), or (ii) substantially reducing or removing this mechanistic interpretation from the main text and presenting it instead as a brief exploratory observation. Clarifying this distinction would better align the interpretation with the evidence presented.
4) Concern #4 questioned whether performance had saturated at 50 training individuals and requested a learning-curve analysis to distinguish data limitation from architectural limitation. The authors addressed this by adding learning curves across increasing training sizes (n = 50, 100, 150, 200), showing that seen-gene performance increases with diminishing returns, whereas zero-shot unseen-gene performance remains near zero across all sizes.
We appreciate this addition, which directly addresses the concern. The results demonstrate that (i) few-shot seen-gene

prediction remains data-limited rather than saturated at n = 50, and (ii) zero-shot failure is not rescued by increasing sample size, supporting the conclusion that cross-locus transfer is the main bottleneck.

Given its conceptual clarity, we suggest considering promotion of this learning-curve analysis from supplementary to a main figure, as it cleanly separates statistical limitation (within-gene scaling) from cross-gene generalization failure, which would sharpen the central message of the study.

Responses to
Comments of reviewers

xDecoder unlocks the potential of genomic foundation models for few-shot personal gene expression prediction

Shumin Li, Ruibang Luo, Yuanhua Huang

Manuscript Number: MSB-2025-13544

Point-by-point responses to the comments of the editor:

Thank you for the submission of your revised manuscript to Molecular Systems Biology. Based on the reviewer feedback and additional advice from an expert advisor, I am pleased to inform you that we will be able to accept your manuscript pending the following final amendments and appropriate response to all remaining comments of the reviewers. Please note that to ensure accuracy of the responses to the reviewers' comments, we may have a reviewer or advisor look over your responses and revisions.

1) We require a complete account in our submission system for corresponding authors - currently the institutional address, ZIP code, phone number are missing for Dr. Huang.

Response: Dr. Huang's account has been updated now.

2) Please note that we now mandate that all corresponding authors list an ORCID digital identifier. Currently an ORCID ID is missing for Dr. Ruibang Luo. An email with instructions to Dr. Luo was sent on May 15th, 2026. We encourage all authors to supply an ORCID identifier, which will be linked to their name for unambiguous name identification.

Response: Dr. Ruibang Luo's ORCID has been added now.

3) Please format the Data availability section according to the example below. "The datasets and computer code produced in this study are available in the following databases: - Chip-Seq data: Gene Expression Omnibus GSE46748 (https://www.ncbi.nlm.nih.gov/geo/query/acc.cgi?acc=GSE46748) - Modeling computer scripts: GitHub (https://github.com/SysBioChalmers/GECKO/releases/tag/v1.0) - [data type]: [full name of the resource] [accession number/identifier] ([doi or URL or identifiers.org/DATABASE:ACCESSION])"

Response: We have updated the manuscript accordingly.

4) Please note that the Data Availability section should only include data and code that have been generated in the course of the current study. For any publicly available data that have been reanalyzed, please move this information to the Methods and potentially include a data citation (see next point).

Response: We have updated the manuscript accordingly.

5) Our journal encourages inclusion of *data citations in the reference list* to directly cite datasets that were re-used and obtained from public databases. Data citations in the article text are distinct from normal bibliographical citations and should directly link to the database records from which the data can be accessed. In the main text, data citations are formatted as follows: "Data ref: Smith et al, 2001" or "Data ref: NCBI Sequence Read Archive PRJNA342805, 2017". In the Reference list, data citations must be labeled with "[DATASET]". A data reference must provide the database name, accession number/identifiers and a resolvable link to the landing page from which the data can be accessed at the end of the reference. Further instructions are available at: https://link.springer.com/journal/44320/submission-guidelines#cms-Reference-guidelines

Response: We have updated the manuscript accordingly.

6) Please rename "Competing Interests" to "Disclosure and competing interests statement" and ensure that this is a separate section of the manuscript. We updated our journal's competing interests policy in January 2022 and request authors to consider both actual and perceived competing interests. Please review the policy https://link.springer.com/partners/embo-press/editorial-policies#Competing%20interest%20disclosures and update your competing interests if necessary.

Response: The section named has been updated to "Disclosure and competing interests statement"

7) Please remove the author contributions from the manuscript and specify author contributions in our submission system. CRediT has replaced the traditional author contributions section because it offers a systematic machine-readable author contributions format that allows for more effective research assessment. You are encouraged to use the free text boxes beneath each contributing author's name to add specific details on the author's contribution. More information is available in our guide to authors: https://link.springer.com/journal/44320/submission-guidelines#cms-Manuscript-organisation-and-formatting

Response: we have removed the author contributions from the manuscript and specified the author contributions in the submission system.

8) Please note that funding information should be given in an "Acknowledgements" section. Please ensure that all funding sources are entered into the manuscript submission system the same as in the manuscript.

Response: we updated the funding sources in the "Acknowledgements" section and the submission system.

9) Please remove the "Declarations" and "Supplementary information" sections of the manuscript. In addition, the "Background" section should be renamed to "Introduction".

Response: we have updated the manuscript correspondingly.

10) Please correct the reference citation in the reference list to be alphabetical (not numerical). Where there are more than 10 authors on a paper, only the first 10 should be listed, followed by "et al.". DOIs should only be used for preprints and datasets that have not

been published yet. Please check "Author Guidelines" for more information: https://link.springer.com/journal/44320/submission-guidelines#cms-Reference-guidelines

Response: we have updated the references correspondingly.

11) In the Methods, please take care of the following: - The Materials and Methods section should be renamed to "Methods".

Response: we have updated the manuscript correspondingly.

12) Please place individual sections of the manuscript in the following order: Title page - Abstract & Keywords - Introduction - Results - Discussion - Methods - Data Availability - Acknowledgements - Disclosure and Competing Interests Statement - References - Figure Legends - (Main Tables with legends if applicable) - Expanded View Figure Legends.

Response: we have updated the manuscript correspondingly.

13) For the figures and figure legends, please take care of the following:
- Please remove all figures from main manuscript file and leave only main figure legends placed after the references. Main and EV figures should be uploaded as individual, high-resolution files. Please check "Author Guidelines" for more information: https://link.springer.com/journal/44320/submission-guidelines#cms-Expanded-View-data

Response: We have updated accordingly.

- Please make sure to update the callouts of all figures in the main manuscript text such that they appeal sequentially. In addition, currently figure callouts are missing for the panels of the Appendix figures and Table S3.

Response: We have updated the callouts correspondingly and added the callout of Table S3 in P9, line 6-7.

- Please note that the exact p values are not provided in the legends of figures 2A, D

Response: we have updated the legend as: "*Exact p-values for paired comparisons between Borzoi and xDecoder (rp-Borzoi) within each bin, from left to right, are 0.738, 2.40 × 10−5, 0.0374, 0.00408, 0.0234, and 0.0214 by two-sided paired t-test.*".

- Please note that the box plots need to be defined in terms of minima, maxima, centre, bounds of box and whiskers, and percentile in the legends of figures 2D, 3A

Response: we have updated the description as: "*The centre line indicates the median; box bounds indicate the 25th and 75th percentiles; whiskers extend to the lowest and highest values within 1.5× the interquartile range; Overlaid points represent individual genes and show the full distribution, including values outside the whiskers*."

- Please note that information related to n is missing in the legend of figure 2D

Response: we have updated the related n to the Fig. 2D's X-axis.

- Please note that the measure of center for the error bars needs to be defined in the legend of figure 2A

Response: we have updated the description as "*Error bars indicate 95% confidence intervals estimated for **the mean** from 1,000 bootstrap samples. *** indicates p < 0.001 by two-sided paired t-test; exact p-values for marked comparisons, from left to right, are 1.44 × 10−8, 7.57 × 10−9, 5.58 × 10−23, 1.43 × 10−15, and 1.33 × 10−24*".

14) The title page of the Appendix should contain "Appendix for + manuscript title" (author and affiliation list not necessary) and a table of contents with the page numbers for the listed items. The nomenclature should be Appendix Figure Sx throughout the manuscript and Appendix PDF.

Response: we have updated the manuscript accordingly.

15) Synopsis: - Synopsis image: Please provide a graphic that summarises the main findings of the manuscript on a glance and upload it as a high-resolution jpeg file 550 pixels wide x 300 pixels high.

Response: we have uploaded it correspondingly.

- Synopsis text: Please provide a separate word document including a short standfirst (maximum of 300 characters, including spaces) and up to 5 bullet points to summarise the key NEW findings. They should be designed to be complementary to the abstract - i.e. not repeat the same text. We encourage inclusion of key acronyms and quantitative information (maximum of 30 words / bullet point). Please use the passive voice.

Response: we have uploaded it correspondingly.

- Please check your synopsis text and image before submission with your revised manuscript. Please be aware that in the proof stage minor corrections only are allowed (e.g., typos).

Response: noted with thanks.

16) Source Data should be organized as a single source data file (zipped) per figure for main figures, with the panels clearly visible in the folder structure instead of a single excel file for all Source Data. e.g. all the Source data files for figure 1 need to be saved in a single folder and this needs to be zipped and then uploaded as "SD figure 1.zip" file.

Response: we have updated it accordingly.

17) As part of the EMBO Publications transparent editorial process initiative (see our policy here: https://www.embopress.org/transparent-process#Review_Process), Molecular Systems Biology will publish online a Peer Review File (PRF) to accompany accepted manuscripts. This file will be published in conjunction with your paper and will include the anonymous referee reports, your point-by-point response and all pertinent correspondence relating to the manuscript. Let us know whether you agree with the publication of the PRF and as here, if you want to remove or not any figures from it prior to publication. Please note that the Authors checklist will be published at the end of the PRF.

Response: we agree with the publication of the PRF and do not request removal of any figures.

18) After your paper is published, we may promote it on social media. If you have any handles or hashtags for Bluesky you would like included, please let us know.

Response: thanks; you may add @YuanhuaHuang (same for Bluesky and X).

19) Please provide a point-by-point letter INCLUDING my comments as well as the reviewer's reports and your detailed responses (as Word file). I look forward to reading a new revised version of your manuscript as soon as possible.

Response: thank you for your reviewing and the point-by-point letter attached below.

Yours sincerely,
Poonam Bheda,
PhD Scientific Editor
Molecular Systems Biology

Point-by-point responses to the comments of Reviewer #1:

#1:The authors have addressed my previous concerns well, and the revised manuscript more clearly demonstrates the advantage of xDecoder over existing sequence-based models. However, I remain somewhat concerned that the absolute predictive performance is still modest. In many settings, the reported Spearman correlations remain well below 0.5, and zero-shot generalization to unseen genes appears to remain very limited. For instance, the authors report improvements in the few-shot seen-gene setting, but the mean Spearman correlations are still modest, for example around 0.194 for rp-Caduceus versus 0.153 for rp-Borzoi in one main comparison. This raises questions about how reliable the model would be for practical personal gene expression prediction, especially at loci not included in training or in settings without matched regulatory data. I therefore encourage the authors to discuss the limitations of xDecoder more explicitly. In particular, it would be useful to clarify that, although xDecoder improves upon current models, the current performance may still be insufficient for accurate gene-level prediction in many practical applications. The authors may also wish to discuss what levels of predictive accuracy would be needed for downstream biological interpretation, variant prioritization, or clinical relevance, and how future model development or additional multi-omic data might help close this gap.

Response: we thank the reviewer's valuable suggestion and agree that it is vital to explicitly state the limitations of xDecoder, in response, we have updated the conclusion as:

(P8, line 3-7): "***Together, these results outline a roadmap for personalized regulatory modeling: combining pretrained sequence encoders with variant-aware modules and using individual-level multi-omic profiles as auxiliary supervision or intermediate regulatory-state targets to improve DNA-based generalization to unseen loci***."

(P8, line 9-20): "***Several limitations should also be considered. Although xDecoder improves few-shot personal expression prediction over existing sequence-based models, its absolute accuracy remains modest, and should not yet be interpreted as***

sufficient for reliable gene-level prediction from personal genomes. Future work should establish application-specific benchmarks for downstream use. For variant prioritization, an important next step is to systematically evaluate whether model-derived perturbation scores can prioritize eQTL-supported regulatory variants over matched background variants, using eQTL-based AUPRC as a primary metric. Any use in clinical interpretation would require substantially stronger calibration and independent validation. Finally, the present evaluation is limited to lymphoblastoid cell lines, and extending this framework to larger cohorts, multi-tissue settings, and context-specific personalized regulation remains an important direction."

Point-by-point responses to the comments of Reviewer #2:

#1: I appreciate the authors' transparency and the extensive new analyses provided in the revision. The addition of the ATAC-only baselines is very helpful in clarifying the model's behaviour. For 'seen' genes, the results demonstrate that combining DNA and ATAC yields slightly better predictions than ATAC alone. Whereas for 'unseen' genes (zero-shot learning), the results show that the summed ATAC-only baseline performs competitively with the DNA+ATAC model. This indicates that the DNA sequence does not currently provide additional predictive value beyond the ATAC data in a zero-shot context. For clarity, I think ATAC alone model should be shown in the main figure to highlight this limitation. The rest of my concerns are addressed adequately.

Response: We thank the reviewer for this constructive comment. We agree that including the ATAC-only baseline is important for clarifying the interpretation of the zero-shot results. In response, we have updated Fig. 3A to include ATAC-only results alongside DNA-only and DNA+ATAC models for better clarifying the conclusions.

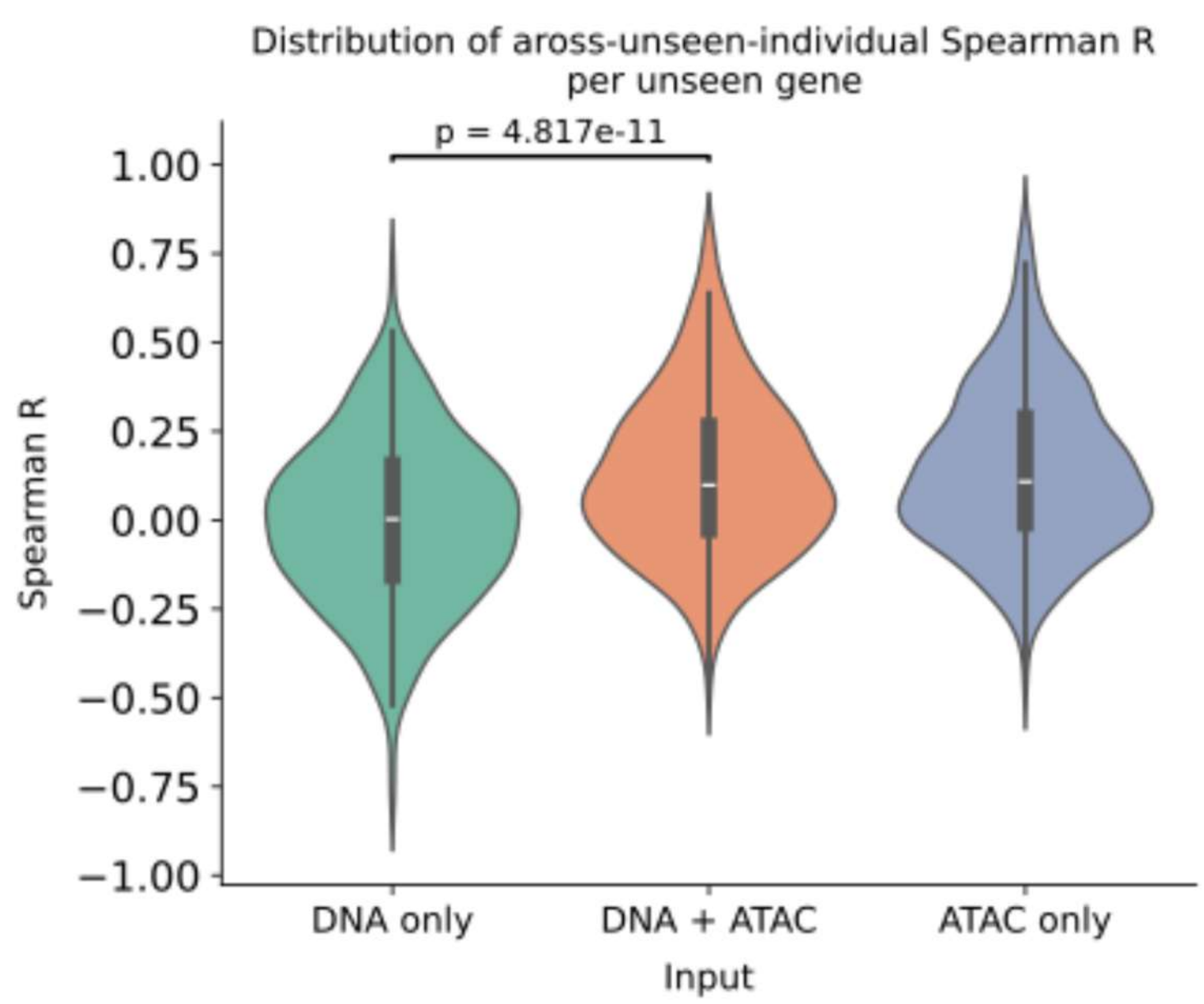

Figure R1 (updated in Main. Fig. 3A). **Distribution of Spearman correlations across unseen individuals for unseen genes using rp-Caduceus with DNA-only input, rp-Caduceus with DNA+ATAC input, and a simple ATAC-only baseline computed by summing ATAC-seq peak signals within the same TSS-centered window**. Genes with identical predictions across individuals were assigned a Spearman R of 0. Significance was assessed between DNA-only and DNA+ATAC using a two-sided paired t-test.

Point-by-point responses to the comments of Reviewer #3:

We thank the authors for their thorough and constructive revisions. The additional analyses address the core technical concerns, yet I feel the manuscript has not been consistently updated to reflect the implications of these new results, and thus requires further revision. Below we assess each major point with particular attention to alignment between results and interpretation:

Response: We thank the reviewer's insightful comments, in response, we have updated the main texts to better reflect the implications of the new results.

#1 Concern #1 questioned whether the ATAC gain reflected genuine DNA×ATAC interaction learning versus conditioning on a regulatory proxy. The authors replied to this by adding supervised and unsupervised ATAC-only baselines, which show that the zero-shot improvement is largely attributable to measured ATAC rather than integrative modeling. We appreciate the additional ATAC-only analyses. However, given that (i) in the seen-gene setting DNA+ATAC is not meaningfully different from DNA-only, and (ii) in the unseen-gene setting ATAC-only matches or exceeds DNA+ATAC, the current framing may still over-attribute the improvement to integrative modeling rather than to the inclusion of a measured regulatory phenotype. For example, the statement that "this bottleneck can be partially overcome by integrating individual-level chromatin accessibility" implies that integration itself overcomes a sequence-model limitation, whereas the ATAC-only controls indicate that the observed gain largely reflects the inclusion of a measured regulatory phenotype rather than integrative modeling. We therefore ask the authors either to revise the abstract and conclusions to explicitly align the claims with these results, or to provide a clear justification for why the current statements remain supported despite the ATAC-only controls. More broadly, clarifying this point will help position the manuscript's true contribution (namely the few-shot seen-gene result and the diagnostic identification of the zero-shot bottleneck) without overstating the role of multi-omic integration.

Response: we thank the reviewer's constructive suggestion and agree that update the statement of abstract and conclusion would help position the manuscript's true contribution. In response, we have updated the manuscript as:

(P1, abstract): "***Experiments incorporating individual-level chromatin accessibility suggested that regulatory-state information important for unseen-locus prediction is not fully captured by current DNA-only models.***"

(P7, line 39-P8, line7): "***Third, incorporating matched individual-level ATAC-seq improves prediction at unseen loci. However, ATAC-only and DNA+ATAC models perform similarly in this setting, suggesting that the improvement is primarily driven by measured chromatin accessibility. Consistently, AlphaGenome-predicted ATAC signals showed only modest agreement with observed ATAC signals (Appendix Fig. S18), suggesting that current DNA-only models may not fully recover individual chromatin-accessibility states that are informative for expression prediction at unseen loci. By contrast, in the seen-gene setting, xDecoder with DNA+ATAC outperforms ATAC-only linear baselines, indicating that sequence-derived information can provide additional signal when paired DNA–RNA training data are available. Together, these results outline a roadmap for personalized regulatory modeling: combining pretrained sequence encoders with variant-aware modules and using individual-level multi-omic profiles as auxiliary supervision or intermediate regulatory-state targets to improve DNA-based generalization to unseen loci."***

#2 Concern #2 questioned whether zero-shot failure was uniform or gene-dependent, and suggested stratifying by gene-level regulatory properties. The authors addressed this by stratifying unseen genes by PrediXcan-based cis-predictability and showing that zero-shot performance increases with stronger cis signal, although it remains below gene-specific supervised models. We appreciate this analysis, which we think suggests that zero-shot performance is gene-dependent rather than uniformly absent. The monotonic improvement with cis-heritability indicates that the models capture biologically meaningful cis signal and that cross-locus transfer is not uniformly broken. In particular, pretrained S2F models (e.g., AlphaGenome) show measurable average signal in moderate/high cis strata. At the same time, the substantial gap relative to the supervised PrediXcan reference and the large within-bin variability indicate that prediction remains unreliable at the per-gene level. We therefore suggest revising the framing to reflect both aspects: zero-shot performance is gene-dependent and partially recoverable for strongly cis-regulated genes, yet remains inconsistent and markedly inferior to gene-specific supervised models. The authors may of course disagree with this interpretation and are welcome to clarify why they consider the current framing of "failure" to be more appropriate in light of the stratified results.

Response: We thank the reviewer for this insightful comment. We agree that the stratified analysis indicates that zero-shot performance is gene-dependent rather than uniformly absent. To make this point clearer, we have promoted the cis-predictability analysis (shown in Fig. 2) to the main figure as Fig. 2F and have also revised the manuscript text accordingly.

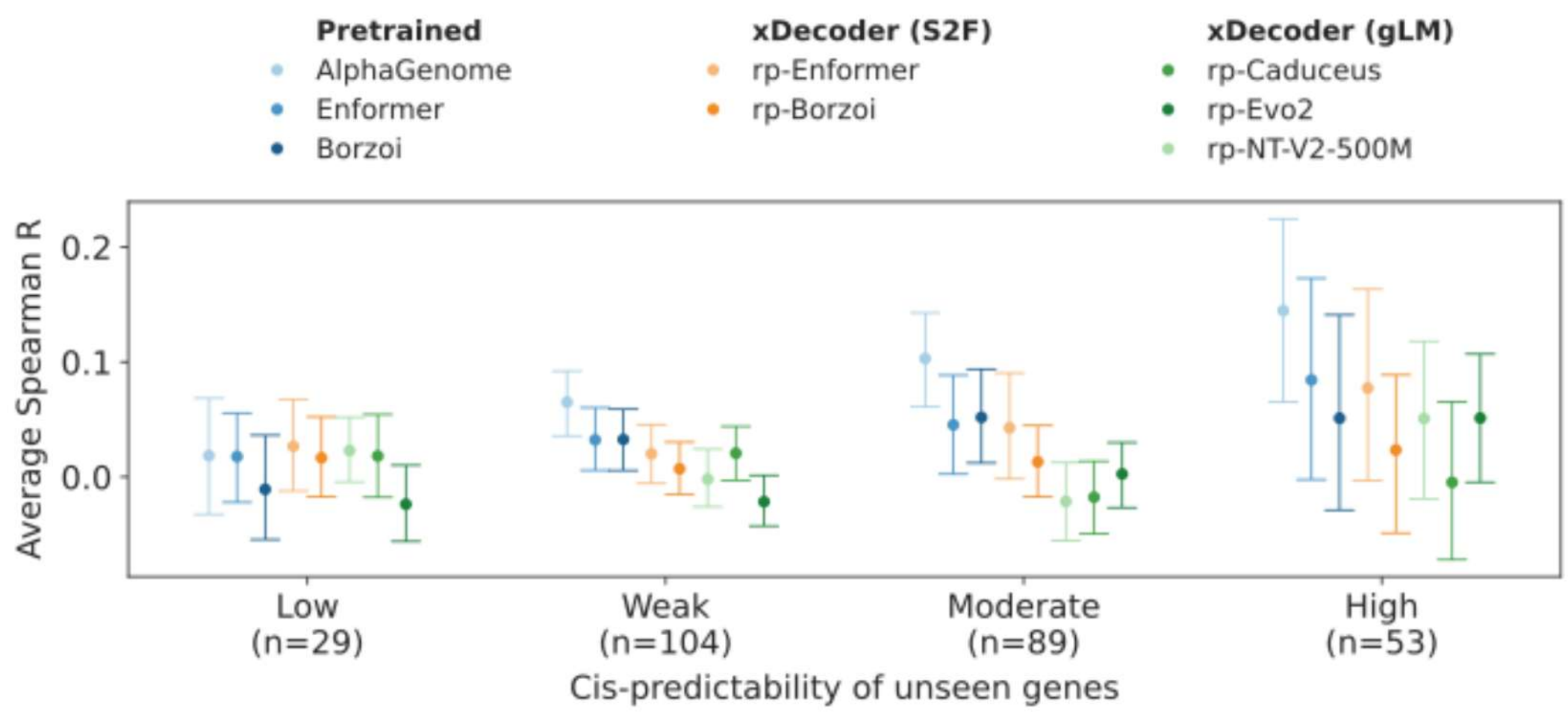

Figure R2 (added as Main. Fig. 2F). Mean zero-shot cross-individual Spearman R for unseen genes stratified by PrediXcan-based cis-predictability using gene-specific ElasticNet models trained on common cis variants (Methods).

(P5, line 27-29): ***"These findings indicate that zero-shot generalization is gene-dependent and partially recoverable for more strongly cis-predictable genes, but remains inconsistent and substantially below gene-specific supervised models, leaving reliable per-gene prediction at unseen loci unresolved."***

#3 Concern #3 questioned whether the cross-ancestry performance drop reflected reliance on population-specific LD structure and requested mechanistic diagnostics (e.g., allele frequency shifts, variant novelty, LD differences). The authors addressed this by adding gene-level analyses of novel variant rate, allele-frequency shift, and LD-structure similarity, showing that no single factor fully explains the EUR-to-YRI performance gap, although larger gaps are associated with higher variant novelty and lower LD similarity. We appreciate the added diagnostic analyses. However, although the results are explicitly inconclusive, the current framing still places interpretive emphasis on LD-structure differences, which risks overstating the explanatory strength of the analysis. We therefore suggest either (i) strengthening the analysis to more directly support a mechanistic claim regarding LD reliance (e.g., clearer quantification of effect sizes or multivariate modeling), or (ii) substantially reducing or removing this mechanistic interpretation from the main text and presenting it instead as a brief exploratory observation. Clarifying this distinction would better align the interpretation with the evidence presented.

Response: We thank the reviewer for this helpful suggestion. We agree that the cross-ancestry diagnostic analyses should be presented as exploratory observations rather than as evidence for a specific LD-based mechanism. In response, we have revised the main text as:

(P5, line 5-7): "***These results suggest that ancestry-specific variation and local LD differences may be associated with reduced transferability, but they do not establish a single mechanistic explanation for the cross-ancestry gap***."

#4 Concern #4 questioned whether performance had saturated at 50 training individuals and requested a learning-curve analysis to distinguish data limitation from architectural limitation. The authors addressed this by adding learning curves across increasing training sizes (n = 50, 100, 150, 200), showing that seen-gene performance increases with diminishing returns, whereas zero-shot unseen-gene performance remains near zero across all sizes. We appreciate this addition, which directly addresses the concern. The results demonstrate that (i) few-shot seen-gene prediction remains data-limited rather than saturated at n = 50, and (ii) zero-shot failure is not rescued by increasing sample size, supporting the conclusion that cross-locus transfer is the main bottleneck. Given its conceptual clarity, we suggest considering promotion of this learning-curve analysis from supplementary to a main figure, as it cleanly separates statistical limitation (within-gene scaling) from cross-gene generalization failure, which would sharpen the central message of the study.

Response: We agree with the reviewer's helpful suggestion that learning-curve analysis is important to clarify our conclusion that increasing training individual sample size does not help for zero-shot generalization. In response, we have added the following Fig. R3 to the Main. Fig. 2G and updated the manuscript correspondingly.

(P5, line 32-37): ***"Seen-gene prediction across unseen individuals improved with more training individuals, with diminishing returns at higher n (Fig. 2G), indicating that few-shot prediction had not saturated at n = 50. In contrast, zero-shot prediction for unseen genes remained close to zero across all training sizes (Fig. 2G), suggesting that this limitation is not simply due to the number of training individuals, but reflects a persistent cross-locus generalization bottleneck."***

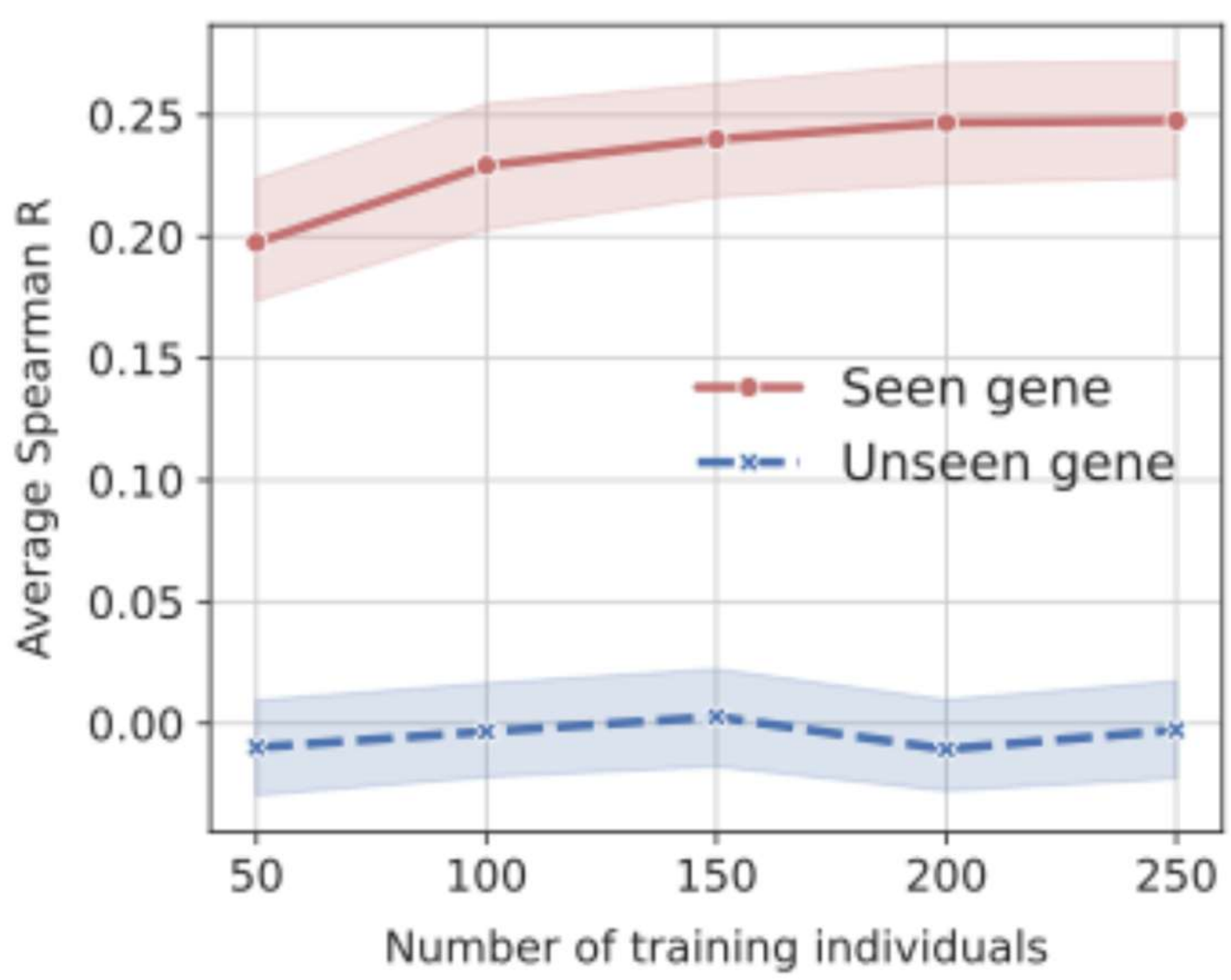

Figure R3 (added as Main. Fig. 2G). Learning curves for seen- and unseen-gene prediction using rp-Caduceus with a 64-kb TSS-centered input.

14th Jul 2026
Manuscript number: MSB-2025-13544RR
Title: xDecoder unlocks the potential of genomic foundation models for few-shot personal gene expression prediction

Dear Prof Huang,

Thank you again for sending us your revised manuscript. We are now satisfied with the modifications made and I am pleased to inform you that your paper has been accepted for publication.

Your manuscript will be processed for publication by EMBO Press. It will be copy edited and you will receive page proofs prior to publication. Please note that you will be contacted by Springer Nature Author Services to complete licensing and payment information.

You may qualify for financial assistance for your publication charges - either via a Springer Nature fully open access agreement or an EMBO initiative. Check your eligibility: https://link.springer.com/journal/44320/how-to-publish-with-us

Should you be planning a Press Release on your article, please notify the production team via this form in order to coordinate publication and release dates: https://forms.cloud.microsoft/e/eH4A8JVP6g

If you have any questions, please do not hesitate to contact the Editorial Office. Thank you for your contribution to Molecular Systems Biology.

Yours sincerely,

Sincerely,

Poonam Bheda, PhD
Scientific Editor
Molecular Systems Biology

>>> Please note that it is Molecular Systems Biology policy for the transcript of the editorial process (containing referee reports and your response letter) to be published as an online supplement to each paper. If you do NOT want this, you will need to inform the Editorial Office via email immediately. More information is available here: https://link.springer.com/partners/embo-press/editorial-policies#Peer%20review

EMBO Press Author Checklist

Corresponding Author Name: Yuanhua Huang
Journal Submitted to: Molecular Systems Biology
Manuscript Number: MSB-2025-13544

USEFUL LINKS FOR COMPLETING THIS FORM
The EMBO Journal - Author Guidelines
EMBO Reports - Author Guidelines
Molecular Systems Biology - Author Guidelines
EMBO Molecular Medicine - Author Guidelines

Reporting Checklist for Life Science Articles (updated January 2022)
This checklist is adapted from Materials Design Analysis Reporting (MDAR) Checklist for Authors. MDAR establishes a minimum set of requirements in transparent reporting in the life sciences (see Statement of Task: 10.31222/osf.io/9sm4x). Please follow the journal's guidelines in preparing your manuscript.
Please note that a copy of this checklist will be published alongside your article.

Abridged guidelines for figures
1. Data
The data shown in figures should satisfy the following conditions:
- the data were obtained and processed according to the field's best practice and are presented to reflect the results of the experiments in an accurate and unbiased manner.
- ideally, figure panels should include only measurements that are directly comparable to each other and obtained with the same assay.
- plots include clearly labeled error bars for independent experiments and sample sizes. Unless justified, error bars should not be shown for technical
- if n<5, the individual data points from each experiment should be plotted. Any statistical test employed should be justified.
- Source Data should be included to report the data underlying figures according to the guidelines set out in the authorship guidelines on Data Presentation.

2. Captions
Each figure caption should contain the following information, for each panel where they are relevant:
- a specification of the experimental system investigated (eg cell line, species name).
- the assay(s) and method(s) used to carry out the reported observations and measurements.
- an explicit mention of the biological and chemical entity(ies) that are being measured.
- an explicit mention of the biological and chemical entity(ies) that are altered/varied/perturbed in a controlled manner.
- the exact sample size (n) for each experimental group/condition, given as a number, not a range;
- a description of the sample collection allowing the reader to understand whether the samples represent technical or biological replicates (including how many animals, litters, cultures, etc.).
- a statement of how many times the experiment shown was independently replicated in the laboratory.
- definitions of statistical methods and measures:
  - common tests, such as t-test (please specify whether paired vs. unpaired), simple χ2 tests, Wilcoxon and Mann-Whitney tests, can be unambiguously identified by name only, but more complex techniques should be described in the methods section;
  - are tests one-sided or two-sided?
  - are there adjustments for multiple comparisons?
  - exact statistical test results, e.g., P values = x but not P values < x;
  - definition of 'center values' as median or average;
  - definition of error bars as s.d. or s.e.m.

Please complete ALL of the questions below.
Select "Not Applicable" only when the requested information is not relevant for your study.

Materials

Newly Created Materials	Information included in the manuscript?	In which section is the information available? (Reagents and Tools Table, Materials and Methods, Figures, Data Availability Section)
New materials and reagents need to be available; do any restrictions apply?	Not Applicable	

Antibodies	Information included in the manuscript?	In which section is the information available? (Reagents and Tools Table, Materials and Methods, Figures, Data Availability Section)
For **antibodies** provide the following information:<br>- Commercial antibodies: RRID (if possible) or supplier name, catalogue number and or/clone number<br>- Non-commercial: RRID or citation	Not Applicable	

DNA and RNA sequences	Information included in the manuscript?	In which section is the information available? (Reagents and Tools Table, Materials and Methods, Figures, Data Availability Section)
**Short novel DNA or RNA including primers, probes**: provide the sequences.	Not Applicable	

Cell materials	Information included in the manuscript?	In which section is the information available? (Reagents and Tools Table, Materials and Methods, Figures, Data Availability Section)
**Cell lines:** Provide species information, strain. Provide accession number in repository **OR** supplier name, catalog number, clone number, and/**OR** RRID.	Not Applicable	
**Primary cultures:** Provide species, strain, sex of origin, genetic modification status.	Not Applicable	
Report if the cell lines were recently **authenticated** (e.g., by STR profiling) and tested for mycoplasma contamination.	Not Applicable	

Experimental animals	Information included in the manuscript?	In which section is the information available? (Reagents and Tools Table, Materials and Methods, Figures, Data Availability Section)
**Laboratory animals or Model organisms:** Provide species, strain, sex, age, genetic modification status. Provide accession number in repository **OR** supplier name, catalog number, clone number, **OR** RRID.	Not Applicable	
**Animal observed in or captured from the field:** Provide species, sex, and age where possible.	Not Applicable	
Please detail **housing and husbandry conditions**.	Not Applicable	

Plants and microbes	Information included in the manuscript?	In which section is the information available? (Reagents and Tools Table, Materials and Methods, Figures, Data Availability Section)
**Plants:** provide species and strain, ecotype and cultivar where relevant, unique accession number if available, and source (including location for collected wild specimens).	Not Applicable	
**Microbes:** provide species and strain, unique accession number if available, and source.	Not Applicable	

Human research participants	Information included in the manuscript?	In which section is the information available? (Reagents and Tools Table, Materials and Methods, Figures, Data Availability Section)
If collected and within the bounds of privacy constraints report on age, sex and gender or ethnicity for all study participants.	Not Applicable	

Core facilities	Information included in the manuscript?	In which section is the information available? (Reagents and Tools Table, Materials and Methods, Figures, Data Availability Section)
If your work benefited from core facilities, was their service mentioned in the acknowledgments section?	Not Applicable	

Design

Study protocol	Information included in the manuscript?	In which section is the information available? (Reagents and Tools Table, Materials and Methods, Figures, Data Availability Section)

If study protocol has been **pre-registered, provide DOI in the manuscript**. For clinical trials, provide the trial registration number **OR** cite DOI.	Not Applicable	
Report the **clinical trial registration number** (at ClinicalTrials.gov or equivalent), where applicable.	Not Applicable	

Laboratory protocol	Information included in the manuscript?	In which section is the information available? (Reagents and Tools Table, Materials and Methods, Figures, Data Availability Section)
Provide DOI OR other citation details if **external detailed step-by-step protocols** are available.	Not Applicable	

Experimental study design and statistics	Information included in the manuscript?	In which section is the information available? (Reagents and Tools Table, Materials and Methods, Figures, Data Availability Section)
Include a statement about **sample size** estimate even if no statistical methods were used.	Not Applicable	
Were any steps taken to minimize the effects of subjective bias when allocating animals/samples to treatment (e.g. **randomization procedure**)? If yes, have they been described?	Not Applicable	
Include a statement about **blinding** even if no blinding was done.	Not Applicable	
Describe **inclusion/exclusion criteria** if samples or animals were excluded from the analysis. Were the criteria pre-established?<br><br>If sample or data points were omitted from analysis, report if this was due to attrition or intentional exclusion and provide justification.	Not Applicable	
For every figure, are **statistical tests** justified as appropriate? Do the data meet the assumptions of the tests (e.g., normal distribution)? Describe any methods used to assess it. Is there an estimate of variation within each group of data? Is the variance similar between the groups that are being statistically compared?	Yes	Figures 2 and 3 captions and main text

Sample definition and in-laboratory replication	Information included in the manuscript?	In which section is the information available? (Reagents and Tools Table, Materials and Methods, Figures, Data Availability Section)
In the figure legends: state number of times the experiment was **replicated** in laboratory.	Not Applicable	
In the figure legends: define whether data describe **technical or biological replicates**.	Not Applicable	

Ethics

Ethics	Information included in the manuscript?	In which section is the information available? (Reagents and Tools Table, Materials and Methods, Figures, Data Availability Section)
Studies involving **human participants**: State details of **authority granting ethics approval** (IRB or equivalent committee(s), provide reference number for approval.	Not Applicable	
Studies involving **human participants**: Include a statement confirming that **informed consent** was obtained from all subjects and that the experiments conformed to the principles set out in the WMA Declaration of Helsinki and the Department of Health and Human Services Belmont Report.	Not Applicable	
Studies involving **human participants:** For publication of **patient photos**, include a statement confirming that consent to publish was obtained.	Not Applicable	
Studies involving experimental **animals**: State details of **authority granting ethics approval** (IRB or equivalent committee(s), provide reference number for approval. Include a statement of compliance with ethical regulations.	Not Applicable	
Studies involving **specimen and field samples:** State if relevant **permits** obtained, provide details of authority approving study; if none were required, explain why.	Not Applicable	

Dual Use Research of Concern (DURC)	Information included in the manuscript?	In which section is the information available? (Reagents and Tools Table, Materials and Methods, Figures, Data Availability Section)
Could your study fall under dual use research restrictions? Please check biosecurity documents and list of **select agents and toxins** (CDC): https://www.selectagents.gov/sat/list.htm	Not Applicable	
If you used a select agent, is the security level of the lab appropriate and reported in the manuscript?	Not Applicable	
If a study is subject to dual use research of concern regulations, is the name of the **authority granting approval and reference number** for the regulatory approval provided in the manuscript?	Not Applicable	

Reporting

The MDAR framework recommends adoption of discipline-specific guidelines, established and endorsed through community initiatives. Journals have their own policy about requiring specific guidelines and recommendations to complement MDAR.

Adherence to community standards	Information included in the manuscript?	In which section is the information available? (Reagents and Tools Table, Materials and Methods, Figures, Data Availability Section)
State if relevant guidelines or checklists (e.g., **ICMJE, MIBBI, ARRIVE, PRISMA**) have been followed or provided.	Not Applicable	
For **tumor marker prognostic studies,** we recommend that you follow the **REMARK** reporting guidelines (see link list at top right). See author guidelines, under 'Reporting Guidelines'. Please confirm you have followed these guidelines.	Not Applicable	
For **phase II and III randomized controlled trials**, please refer to the **CONSORT** flow diagram (see link list at top right) and submit the CONSORT checklist (see link list at top right) with your submission. See author guidelines, under 'Reporting Guidelines'. Please confirm you have submitted this list.	Not Applicable	

Data Availability

Data availability	Information included in the manuscript?	In which section is the information available? (Reagents and Tools Table, Materials and Methods, Figures, Data Availability Section)
Have **primary datasets** been deposited according to the journal's guidelines (see 'Data Deposition' section) and the respective accession numbers provided in the Data Availability Section?	Not Applicable	
Were **human clinical and genomic datasets** deposited in a public access-controlled repository in accordance to ethical obligations to the patients and to the applicable consent agreement?	Not Applicable	
Are **computational models** that are central and integral to a study available without restrictions in a machine-readable form? Were the relevant accession numbers or links provided?	Yes	We provided this information in the Data Availability section. The repository link is: https://github.com/StatBiomed/xDecoder/
If publicly available data were reused, provide the respective **data citations in the reference list**.	Yes	Relevant data citations are provided as: BioStudies (2013). RNA-sequencing of 465 lymphoblastoid cell lines from the 1000 Genomes. https://www.ebi.ac.uk/biostudies/arrayexpress/studies/E-GEUV-1.10 [DATASET]; Github (2024). Processed geuvadis log-tpm expression matrix and gene annotations. https://github.com/ni-lab/finetuning-enformer/tree/main/process_geuvadis_data. [DATASET];Zenodo (2019). Processed ATAC-seq data for 100 LCLs. https://zenodo.org/records/1405945. [DATASET]
