# Supplementary material for: xDecoder unlocks the potential of genomic foundation models for few-shot personal gene expression prediction
Source: Mol Syst Biol. 2026 Jul 28;22(11):1672–87. doi: 10.1038/s44320-026-00238-1 (PMC13728230; doi:10.1038/s44320-026-00238-1)
Supplement: Supplementary file 3 [file 44320_2026_238_MOESM6_ESM.pdf]

Expanded View Figures

Figure EV1. Individual fine-tuning improves few-shot prediction for seen genes.

(**A**) Paired comparison of across-unseen-individual Spearman correlations between reference-trained and individually fine-tuned xDecoder models across seen genes. Each point represents one gene, and colors indicate PrediXcan-based *cis*-predictability groups. (**B**) Permutation-based significance analysis of cross-individual prediction performance. For each gene, observed expression values were permuted across testing individuals to generate a gene-specific null distribution ($n = 2000$), and genes with one-sided empirical $P < 0.1$ were defined as significant.

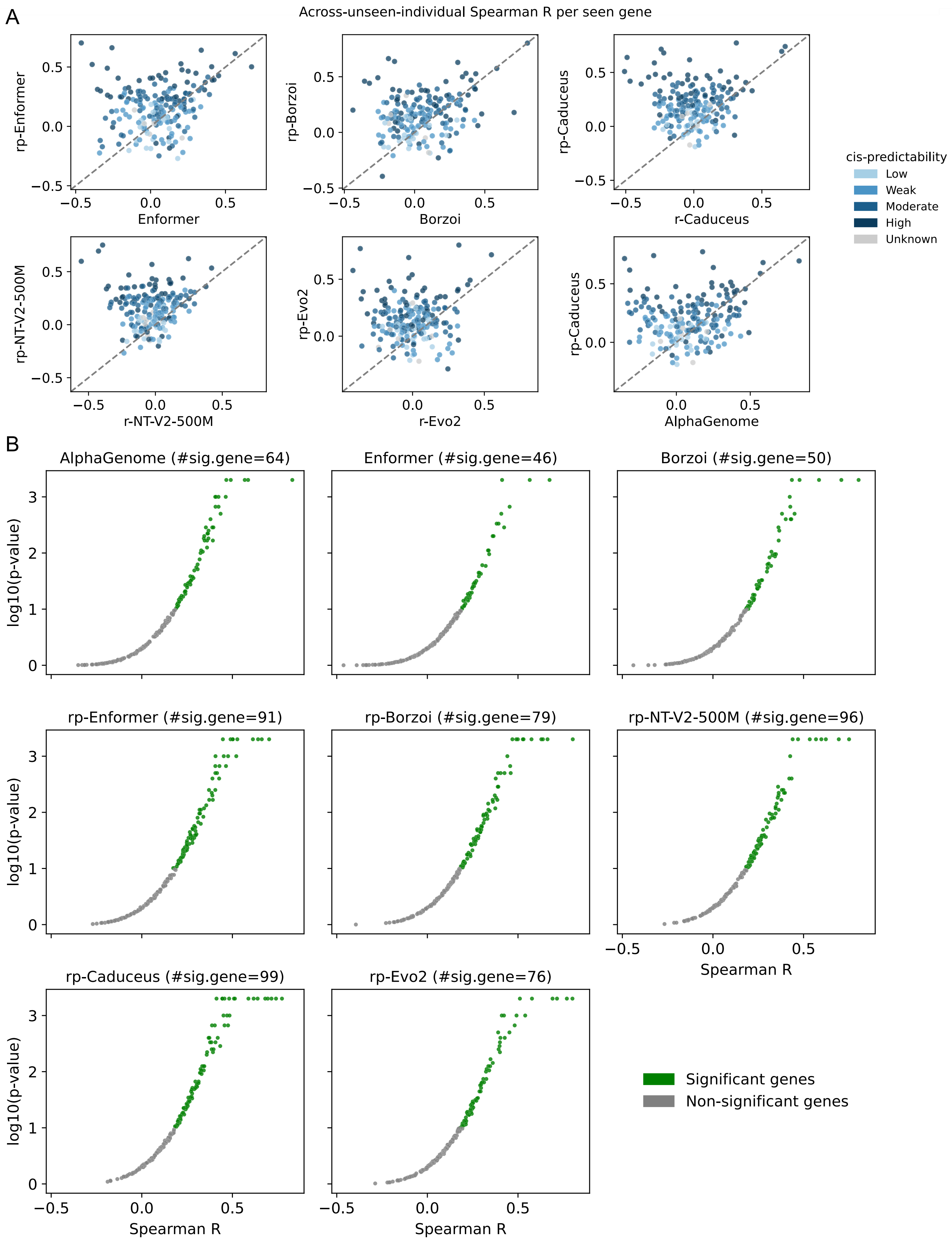

A
Across-unseen-individual Spearman R per seen gene
rp-Enformer
Enformer
rp-Borzoi
Borzoi
rp-Caduceus
r-Caduceus
rp-NT-V2-500M
r-NT-V2-500M
rp-Evo2
r-Evo2
rp-Caduceus
AlphaGenome
cis-predictability
Low
Weak
Moderate
High
Unknown
B
AlphaGenome (#sig.gene=64)
Enformer (#sig.gene=46)
Borzoi (#sig.gene=50)
rp-Enformer (#sig.gene=91)
rp-Borzoi (#sig.gene=79)
rp-NT-V2-500M (#sig.gene=96)
rp-Caduceus (#sig.gene=99)
rp-Evo2 (#sig.gene=76)
log10(p-value)
Spearman R
Significant genes
Non-significant genes

 © The Author(s)

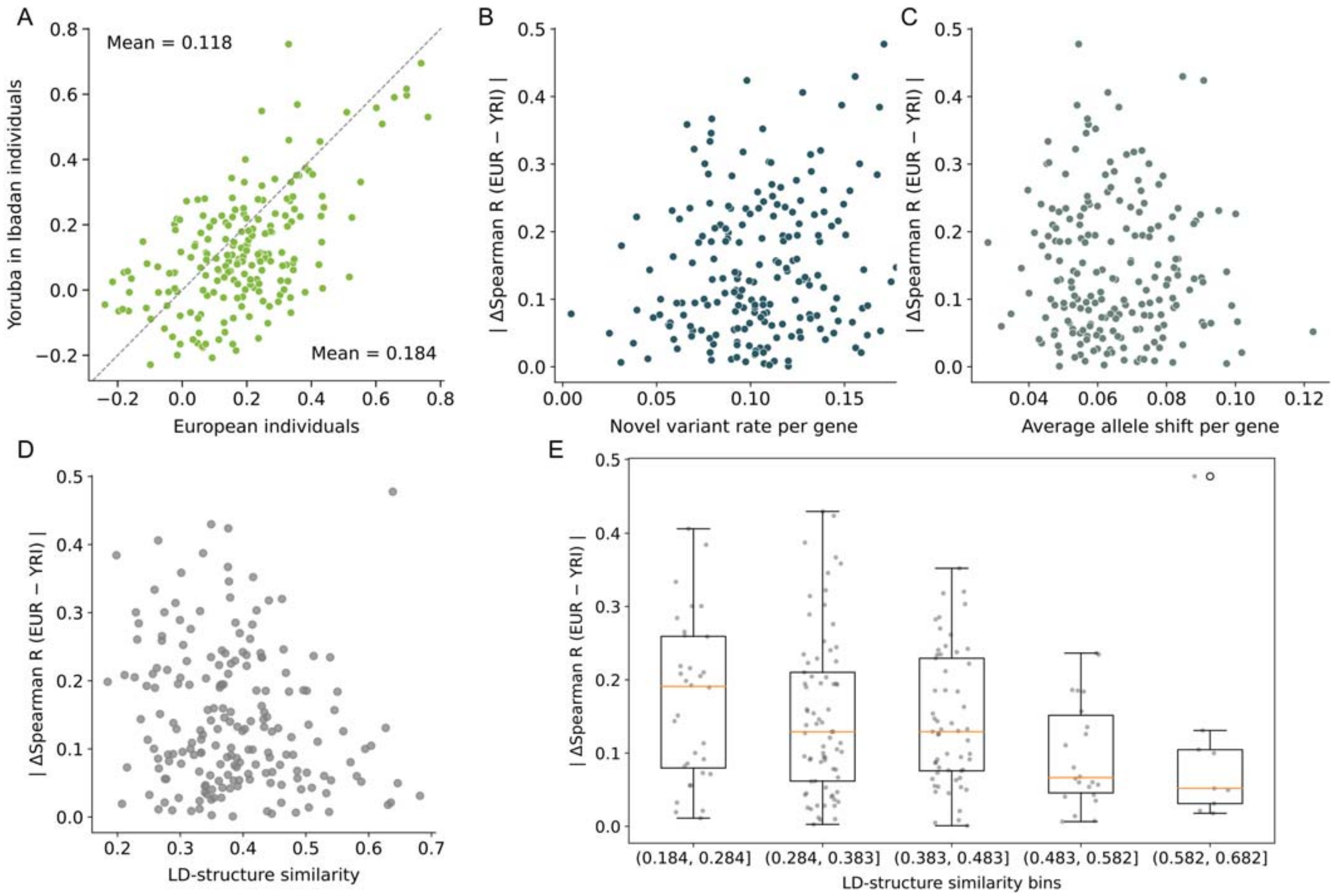

Figure EV2. Analysis of cross-ancestry performance using the rp-Caduceus model.

(**A**) Gene-level prediction performance of the same European-trained model evaluated on European and YRI test individuals. (**B**) Relationship between the absolute EUR-to-YRI performance gap and the novel variant rate per gene, defined as the fraction of common variants (MAF ≥0.05) present in one population but absent in the other within the 131,070 bp TSS-centered context. (**C**) Relationship between the absolute performance gap and mean allele-frequency shift per gene, defined as the average absolute allele-frequency difference between European and YRI individuals across variants in the same region. (**D**) Relationship between the absolute performance gap and LD-structure similarity. LD-structure similarity was measured as the Jaccard index between strong-LD variant-pair sets identified separately in European and YRI populations from the Ensembl 1000 Genomes panel, using $R^2 > 0.8$. Higher values indicate a more similar local LD organization between populations. (**E**) Distribution of the absolute performance gap after grouping genes by LD-structure similarity.

© The Author(s)

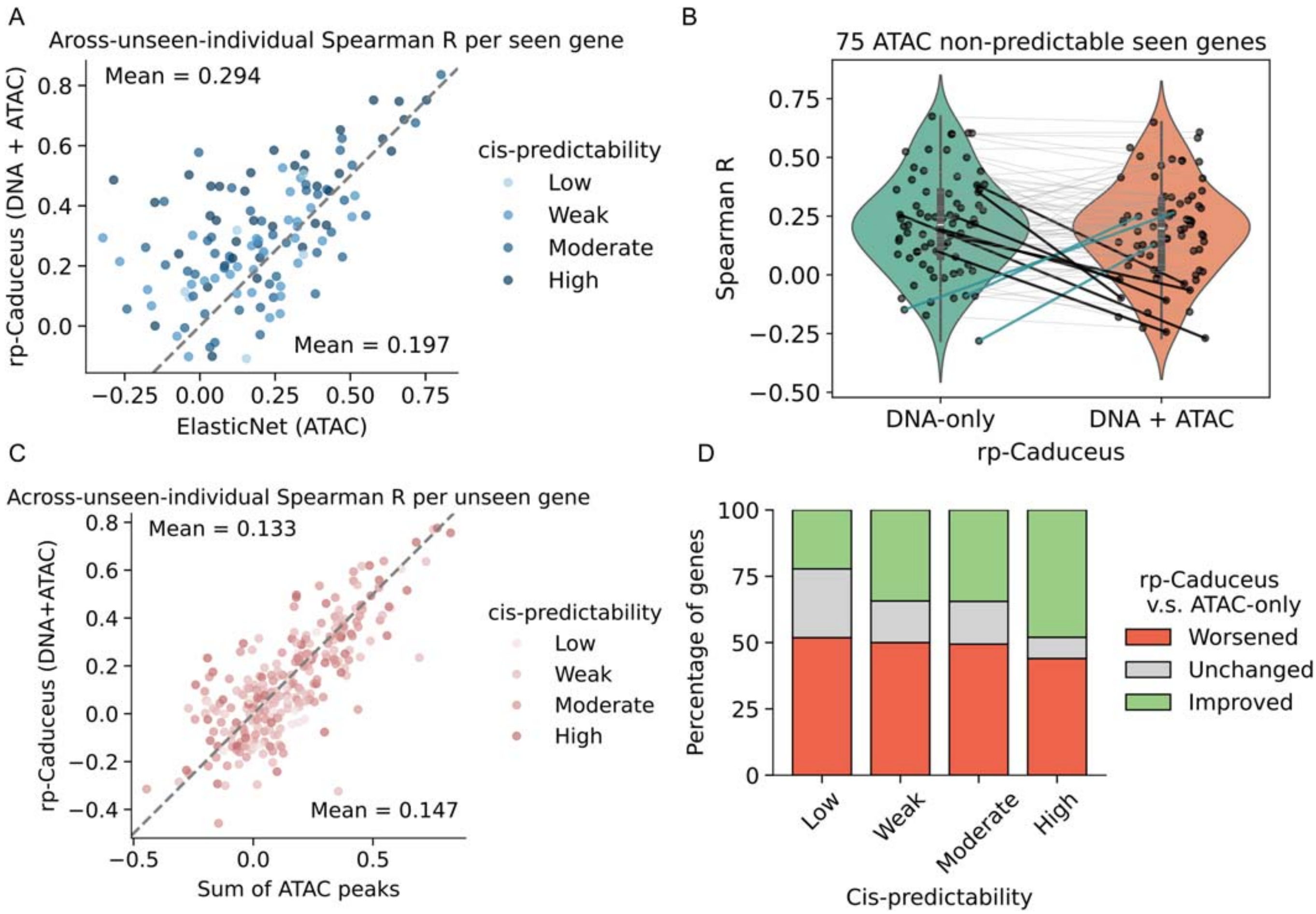

Figure EV3. ATAC-only analyses in seen- and unseen-gene settings.

(**A**) Comparison between a supervised ATAC-only ElasticNet baseline and rp-Caduceus (DNA+ATAC) in the seen-gene setting. For each seen gene, the ATAC-only ElasticNet model was trained using ATAC features summarized within the same 64 kb TSS-centered context window used by rp-Caduceus. Noted that 125 out of 200 seen genes are valid for the ATAC-only model. The center line indicates the median, box bounds indicate the 25th and 75th percentiles, and whiskers extend to the lowest and highest values within 1.5 × the interquartile range. (**B**) Performance of rp-Caduceus with DNA-only and DNA+ATAC inputs on 75 seen genes for which the ATAC-only ElasticNet baseline produced uninformative predictions. (**C**) Comparison between an unsupervised ATAC-only baseline and rp-Caduceus (DNA+ATAC) in the unseen-gene setting. The ATAC-only score was computed by summing the ATAC signal across the same 64 kb genomic context and correlating this score with observed expression across held-out individuals. Noted that 275 out of 287 unseen genes are valid for the ATAC-only model. (**D**) Stratification of unseen genes by *cis*-predictability. Genes were classified as improved, unchanged, or worsened according to the performance difference between rp-Caduceus (DNA+ATAC) and the ATAC-only baseline.

 © The Author(s)
